# Predicting the pathogenicity of bacterial genomes using widely spread protein families

**DOI:** 10.1186/s12859-022-04777-w

**Published:** 2022-06-24

**Authors:** Shaked Naor-Hoffmann, Dina Svetlitsky, Neta Sal-Man, Yaron Orenstein, Michal Ziv-Ukelson

**Affiliations:** 1 Department of Computer Science, Ben-Gurion University of the Negev, Be’er Sheva, Israel; 2 The Shraga Segal Department of Microbiology, Immunology and Genetics, Faculty of Health Sciences, Ben-Gurion University of the Negev, Be’er Sheva, Israel; 3 School of Electrical and Computer Engineering, Ben-Gurion University of the Negev, Be’er Sheva, Israel

**Keywords:** Comparative genomics, Pathogenic bacteria, Commensal bacteria, Opportunistic bacteria, Random forest, Protein families

## Abstract

**Background:**

The human body is inhabited by a diverse community of commensal non-pathogenic bacteria, many of which are essential for our health. By contrast, pathogenic bacteria have the ability to invade their hosts and cause a disease. Characterizing the differences between pathogenic and commensal non-pathogenic bacteria is important for the detection of emerging pathogens and for the development of new treatments. Previous methods for classification of bacteria as pathogenic or non-pathogenic used either raw genomic reads or protein families as features. Using protein families instead of reads provided a better interpretability of the resulting model. However, the accuracy of protein-families-based classifiers can still be improved.

**Results:**

We developed a wide scope pathogenicity classifier (WSPC), a new protein-content-based machine-learning classification model. We trained WSPC on a newly curated dataset of 641 bacterial genomes, where each genome belongs to a different species. A comparative analysis we conducted shows that WSPC outperforms existing models on two benchmark test sets. We observed that the most discriminative protein-family features in WSPC are widely spread among bacterial species. These features correspond to proteins that are involved in the ability of bacteria to survive and replicate during an infection, rather than proteins that are directly involved in damaging or invading the host.

## Introduction

Throughout history, infectious diseases have caused high mortality and morbidity [1]. Despite medical advances and prevention efforts in the last 100 years, infectious diseases remain a significant threat to humanity [2]. Since the 1970s, at least 26 major emerging and reemerging infectious diseases of a bacterial origin have been reported, where most of them originated from the environment [3]. As globalization and environmental changes increase human exposure to diverse bacteria, in the upcoming years we expect to discover new pathogenic bacterial strains, species, or even genera [3].

Recent advances in next-generation-sequencing (NGS) technologies have made bacterial genome sequencing fast and accessible [4]. As a result, public databases contain large numbers of whole genome sequences of diverse bacterial genomes [4], usually along with information that can be used as a proxy to automatically label a genome as pathogenic or non-pathogenic. This information can be then utilized within machine-learning frameworks to predict the pathogenicity of bacterial genomes. Moreover, a systematic genomic comparative analysis across different bacterial genera and phyla can advance our understanding of bacterial pathogenicity mechanisms on a global level.

Bacteria that colonize the human body can be separated into three groups according to their lifestyle: exclusive pathogens, opportunistic pathogens, and commensal non-pathogenic bacteria [5]. A diverse community of trillions of commensal bacteria, many of which are essential for our health, inhabit the human body [6]. By contrast, exclusive or opportunistic pathogens have the ability to invade their hosts and cause disease. Opportunistic pathogens are normally present in the environment or as part of the commensal bacterial population of a host, but may cause a disease following a medical perturbation to the host [7]. Due to the complexity of differentiating exclusive pathogens from opportunistic bacteria in datasets of clinical samples, in this study any strain isolated from an infection is considered to be a pathogen.

With the development of molecular biological techniques, it has become possible to identify genes that contribute to bacterial pathogenesis, denoted as virulence genes [8]. However, virulence genes can also be identified in non-pathogenic strains [5, 9]; thus, a simple possession of some virulence genes does not necessarily indicate that a strain is pathogenic. In addition, relying on known virulence genes for pathogenicity classification can be limiting. A more general approach is to consider all available genes, associated with pathogenic as well as non-pathogenic bacteria, in a given dataset of bacterial genomes.

In recent years, several models were proposed for the classification of a bacterial genome as pathogenic to humans (HP) or non-pathogenic to humans (NHP) [10–15]. These models can help predict the pathogenicity of novel bacterial species, and additionally contribute to our general understanding of the pathogenic lifestyle by analysing important classification features. Previous computational methods for pathogenicity classification can be divided into two main categories: Read-based methods [14–16] and protein-content-based methods [10–13] (reviewed in detail under Additional file 1: Section S1). In a nutshell, read-based classification approaches use short genomic reads as input, while protein-content-based methods characterize a bacterial genome by the presence or absence of protein family members. The advantage of read-based classification approaches over protein content-based ones is that assembly and annotation of reads to protein-coding sequences are not required; thus, they may provide a faster analysis of metagenomic samples. However, read-based methods are more difficult to interpret than protein-content-based methods since they consider only short local patterns as features, disregarding a wider genomic context. In contrast to read-based methods, protein-content-based methods can help in the identification of proteins associated with a pathogenic phenotype [10–13]. Furthermore, some of these methods can even discover novel unannotated proteins related to pathogenicity [12, 13].

In this work, we propose a protein-content-based method for classifying a bacterial genome as pathogenic to humans or not. Our method does not rely on prior knowledge of the taxonomic association of the genome to be classified. In order to avoid the species distribution bias experienced by previous protein-content-based works (Additional file 1: Section S1) and increase the generalization of the model to new species, the classifier is trained on a balanced dataset that includes one strain per species. Additionally, to overcome the runtime bottleneck imposed by clustering proteins into protein families (Additional file 1: Section S1), we propose to harness a feature set composed of Global Protein Families (PGFams) [17], which are available through the PATRIC database [18]. In contrast to Iraola et al. [11], which relied on previously annotated virulence genes, we consider hundreds of thousands of PGFams regardless of their function. The proposed approach, denoted Wide Scope Pathogen Classifier (WSPC), applies a Random Forest (RF) classifier to a dataset of bacterial genomes that belong to a wide range of taxa. Finally, in order to avoid overfitting [19] and enable the generalization of the model to unseen genomes, we apply a feature selection stage that reduces the number of features from $\sim$400,000 to 250 widely spread protein families (“Feature selection of WSPC” Section).

The resulting WSPC classifier is highly accurate. A comparative analysis on a benchmark dataset shows that WSPC outperforms existing protein-content-based and read-based classifiers, achieving a higher balanced accuracy (BACC) value (“Prediction performance comparison on the BacPaCS test set” section). Furthermore, WSPC achieves highly accurate classification results on a large novel test set that consists of a wide range of genera and species, including a subset of genomes belonging to species that were not part of the training set (“Prediction evaluation on the WSPC test set” Section).

An interesting result of our analysis is that our method reveals widely spread protein families associated with pathogenicity. The application of a feature selection procedure that selects highly distributed protein families in combination with a phylogenetically diverse training set, exposes genes involved in the processes of respiration and energy production, DNA repair, metabolism, and stress tolerance. Thus, a unique property of our model is that the most discriminative features consist of genes that allow quick adjustment and survival of the pathogens under challenging conditions, such as during infection, rather than “classical virulence genes” that are directly involved in causing a disease.

## Methods

### Dataset

**Fig. 1:**
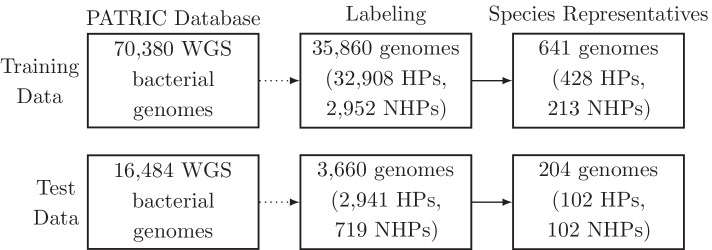
Dataset pre-processing overview. We report the number of genomes in the WSPC training and test data, after each pre-processing step (“Dataset” section). Note that genomes that could not be labeled were removed. WGS: Whole-genome sequences

An overview of the pre-processing steps we performed to create the WSPC dataset is shown in Fig. 1.

#### Collection and pre-processing of the data

The data used in this study was extracted from the PAThosystems Resource Integration Center (PATRIC) [18]. PATRIC collects microbial genomes from GenBank [20] and other sources, and consistently annotates them using the RAST pipeline [21]. In addition, PATRIC provides Global Protein Family (PGFam) annotations [17] that enable comparison between genes across different genomes. Whole-genome sequences (WGS), including both chromosomes and plasmids, of 86,864 human-colonizing bacterial genomes, identified by the phrase “Human, Homo sapiens” or “Homo sapiens” in the field “Host Name”, were downloaded from PATRIC on July 9, 2020. Genomes with “Date Inserted” earlier than November 1, 2019, were used for training, and the rest were used as a held-out test set.

In order to identify human pathogen (HP) and non-human pathogen (NHP) bacteria in our dataset we followed the annotation-based pathogenicity classification method described by Barash et al. [13] with a few modifications we made to improve the annotation. Briefly, this method detects relevant keywords in the metadata fields associated with each genome. We labeled a genome as HP if it was isolated from an infection, and as NHP if it was isolated from a healthy individual or a probiotic supplement (the annotation method is described in detail in Additional file 1: Section S2.1). In addition, we filtered out all genomes with “poor” quality in the “Genome Quality” field.

The labeling and filtering procedure resulted in 35,860 genomes in the training set and 3,660 genomes in the test set that were labeled as HP or NHP, while the remaining genomes were annotated as inconclusive and were therefore removed. The number of genomes per species in our dataset varies greatly: between one to thousands of genomes per species. An over-representation of a single species can cause a sample selection bias and prevent the model from generalizing to new species that were not present in the training set. In addition, the evaluation of a classifier on an unevenly sampled set could be misleading. Therefore, the training and test sets were randomly sub-sampled to include exactly one genome per species, as was previously done by Bartoszewicz et al. [15]. The species of each genome was determined using the NCBI taxonomy database [22]. Note that some genomes are unclassified at the species level, and are typically classified at the genus or at a higher taxonomic level. As these genomes could potentially represent novel species, they were grouped according to their lowest shared taxonomic group instead.

#### Training set

Initially, the training set consisted of 35,860 genomes, where 32,908 ($\sim$92%) were labeled as HP and 2952 ($\sim$8%) were labeled as NHP. To increase the chances of the classifier to uncover highly pathogenic or highly non-pathogenic genomic properties, we aimed to select from each species a genome that represents the tendency of this specific species to be HP or NHP. In addition, we included only species that were mostly pathogenic or non-pathogenic, according to the labels of the genomes belonging to these species. For each species, we defined its *label ratio* as the number of genomes annotated with the minority label divided by the number of genomes annotated with the majority label, where all the genomes across the training and test sets were considered for the label ratio computation. 38 species with a label ratio larger or equal to 0.1 likely represent opportunistic bacteria and were therefore removed from the training set. This group included known opportunistic bacterial species, such as *Bacillus cereus* [23], *Bacteroides fragilis* [24], and *Staphylococcus epidermidis* [25]. For 29 out of the remaining 536 species in the training set, the label ratio was between 0 and 0.1. For these species, the minority labeled genomes were removed from the training set. Additional 105 taxonomic groups in the training set represent genomes that are unclassified at the species level, thus the label ratio is irrelevant for these groups. Finally, we randomly selected one genome from each species in the remaining training set (or from each taxonomic group in the case where the species was not classified). This resulted in training set that contains 213 NHP genomes and 428 HP genomes belonging to 641 different taxonomic groups.

#### WSPC test set

Initially, the test set consisted of 3660 genomes, where 2941 were labeled as HP and 719 were labeled as NHP. First, we grouped the genomes according to their species or according to a higher taxonomic level in cases where the species was unclassified. Then, one genome from each taxonomic group was randomly selected. This resulted in a set of 206 genomes, where 170 of these genomes belong to classified species. Next, all the genomes in the test set were inspected manually to ensure that their labels are correct by reviewing the associated PATRIC metadata. A genome was verified as HP if the isolation source was a diseased individual, and verified as NHP if the isolation source was a healthy individual or a probiotic supplement. Additionally, a literature curation was performed to confirm the corresponding label. Two strains were mislabeled by the automatic annotation and therefore their labels were corrected manually, and two other strains, that could not be validated as HP or NHP, were removed from the test set (further details can be found in Additional file 1: Section S2.2). This process resulted in a non-redundant final test set consisting of 102 HP bacterial genomes and 102 NHP bacterial genomes, belonging altogether to 93 genera (Additional file 1: Table S3). To get a better estimation of model performance on novel species, we created a subset of the test set, which consists only of species that were not part of the training set and hence are new to the classifier. This subset includes only genomes that were classified to known species, resulting in a set of 32 HP genomes and 31 NHP genomes.

### BacPaCS test set

We manually curated the 100 genomes included in the BacPaCS test set using the metadata associated with each genome and the literature. We verified the genome label as HP if it was isolated from a diseased host (based on the PARTIC database entry), and if there was also evidence in the literature that the corresponding species or strain is pathogenic. We verified a genome label as NHP if it was isolated from a healthy host, and if the corresponding species or strain was also described in the literature as commensal or probiotic. We changed the labels of 18 strains from NHP to HP, as these strains were isolated from clinical samples or described in the literature as well-known pathogenic strains. We could not verify the labels of six other strains as HP or NHP. Therefore, these strains were removed from the test set. Further details on the relevant strains and the verification process are described in Additional file 1: Section S2.3. In total, we derived two benchmark test sets:

1. *Benchmark Test 1* The process described above resulted in a set of 94 genomes (78 HP and 16 NHP) whose pathogenicity label we could manually verify.
2. *Benchmark Test 2* To create a species-wise balanced test set, we randomly selected one genome per species from the 94 genomes of Benchmark Test 1. This process resulted in a new balanced subset, which consists of 40 manually labeled genomes (25 HP and 15 NHP). 

A list of all the genomes included in the original BacPaCS test set along with their verified labels, references to relevant studies, as well as an indication of whether each genome was included in Benchmark Test 2, is given in Additional file 1: Table S2.

### Comparative analysis with extant classifiers

The following test sets were used for comparative analysis with extant classifiers.

*BacPaCS test set* The binary predictions of the classifiers BacPaCS, PathogenFinder, and PaPrBaG on the original BacPaCS test set were provided in the supplementary materials of Barash et al. [13]. We used the relevant predictions according to the genomes in Benchmark Test 1 and Benchmark Test 2. We computed the predictions of DeePaC’s two published models, *sensitive LSTM* and *rapid CNN*, according to the description in their paper using simulated read pairs of the BacPaCS test set genomes (published as part of the supplementary materials of DeePaC [15]). As DeePaC predicts a value between 0 and 1 for each read, the prediction is averaged for each read pair, and then averaged again over all read pair predictions for each input genome. A genome with a prediction value greater than 0.5 was predicted as HP, otherwise it was predicted as NHP. Although the models of DeePaC gave different raw prediction values (the mean prediction value of *sensitive LSTM* was 0.84 while the mean prediction value of *rapid CNN* was 0.65), the binary predictions of the two models produced the same sensitivity and specificity scores. PaPrBaG [14] provides 5 models created in 5-fold cross validation, therefore we averaged the results (specificity, sensitivity, and BACC) of the 5 models.

*WSPC test set* We obtained the binary predictions of BacPaCS and PathogenFinder [12] using their published trained models, which take as input a set of proteins for each of the corresponding genomes. We used the whole-data model, which was trained on all the bacteria in their training set, for PathogenFinder.

### Training procedure

**Fig. 2:**
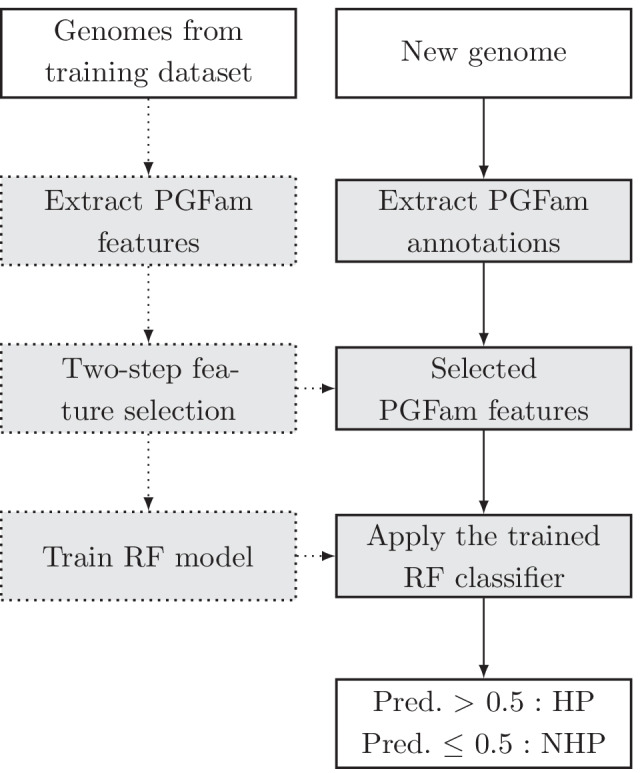
An overview of the classification workflow. Rectangles with dashed lines represent training steps, and rectangles with continuous lines represent prediction steps. Input and output cells are colored in white. PGFam: PATRIC Global Protein Family, RF: Random Forest, Pred.: Predicted Probability

An overview of the workflow of our training procedure and classification approach is illustrated in Fig. 2.

#### Extracting PGFam features

We represent each genome in our dataset as a sequence of PGFam identifiers [17]. PGFams in the PATRIC database were defined by first binning the proteins encoded by the respective genes according to their function, then clustering the proteins in each bin into local genus-level families, and finally by clustering local genus-level families into global families. The PATRIC database provides PGFam annotations for the genes of each bacterial genome in it, and an annotation service for a new genome uploaded by the user. Therefore, each genome in our dataset is represented by a binary feature vector, where each element in the vector corresponds to a PGFam that appears in the training set genomes. An element is set to 1 if the corresponding PGFam is present in the genome, and 0 otherwise.

#### Generating and evaluating a classification model

The RF machine-learning method is widely used for data analysis in bioinformatics [26, 27], as it provides a combination of high prediction performance and model interpretability [28]. RF-based methods construct prediction rules for a classification problem and provide feature importance measures that are automatically computed for each input feature. In this work, we used the RF classifier in the feature selection process (“Feature selection of WSPC” Section) and as the final model. The final RF classifier was trained using feature vectors that contain 250 binary features, each appearing in at least 35 of the 641 genomes of the training set (“Training set” section), where each genome has a binary label of 0 (NHP) or 1 (HP). The RF training procedure was implemented through python module sklearn.ensemble.RandomForestClassifier (n_estimators = 100, min_samples_split = 2, criterion = “gini”) [29].

For classification evaluation, we used Sensitivity (true positive rate), Specificity (true negative rate), and Balanced Accuracy (BACC), which is the mean of the sensitivity and specificity [30]. For ranking evaluation of WSPC, we used the areas under the precision recall (AUPR) [31], and the receiver operation characteristic (AUROC) [32] curves. AUROC was also used for the feature selection parameter tuning (further details can be found in Additional file 1: Section S3.1).

#### Feature selection of WSPC

There are 393,042 PGFam features in our training set (“Training set” section), many of them appearing in only one or a few genomes. Using all available PGFams as features may cause overfitting [19] and prevent the generalization of the model to unseen genomes. In order to select the most discriminative features that appear in a wide range of taxa and that are highly correlated with the target label, we performed a feature selection process using a validation set.

First, the training set genomes (“Training set” section) were sorted by their insertion date to PATRIC. The first 80% of the genomes were assigned to the training set, and the last 20% of the genomes were assigned to the validation set, which consisted of 89 HP genomes and 39 NHP genomes. The partition according to the date of insertion simulates the application in the real-world by training the classifier on genomes that are available in public databases at a certain time point, and then using the classifier to classify newly sequenced genomes. In addition, as the genomes in the training and validation sets belong to different species, this reduces the chance of overfitting, as it enables the assessment of classifier performance on species that are not part of the training set.

The feature selection process consists of two consecutive steps, which are detailed below. First, the *k* most discriminative features are selected according to the Chi-square ($\chi ^2$) score [33] between each feature and the target labels (HP and NHP). Second, features are clustered according to their pairwise correlations, and the most discriminative feature is chosen from each cluster.

*Selecting the Most Discriminative Features.* The $\chi ^2$ score is commonly applied for the selection of the most discriminative features of a classification problem [34–36]. This score is used to determine if there is any association between a categorical feature and the categorical target variable, in our case between a presence or absence of a PGFam and a binary pathogenicity label. A large $\chi ^2$ value indicates a greater probability for dependency between the occurrence of the feature and the pathogenicity label. The *k* most discriminative features were selected based on the top $\chi ^2$ scores between each feature and the target labels (HP and NHP) of the training set genomes. The selection of the *k* features with the highest $\chi ^2$ scores was performed using the class sklearn.feature_selection.SelectKBest of the scikit-learn library [29], where the $\chi ^2$ score function was used.

We tested multiple values of *k* for WSPC, starting from $k=50$ and increasing by 50 until the training set size is reached. For each value of *k*, we trained an RF classifier on the training set and evaluated it on the validation set. The final value of $k=450$ was selected according to the maximum AUROC score achieved by the classifier on the validation set (Additional file 1: Fig. S1.A).

**Fig. 3:**
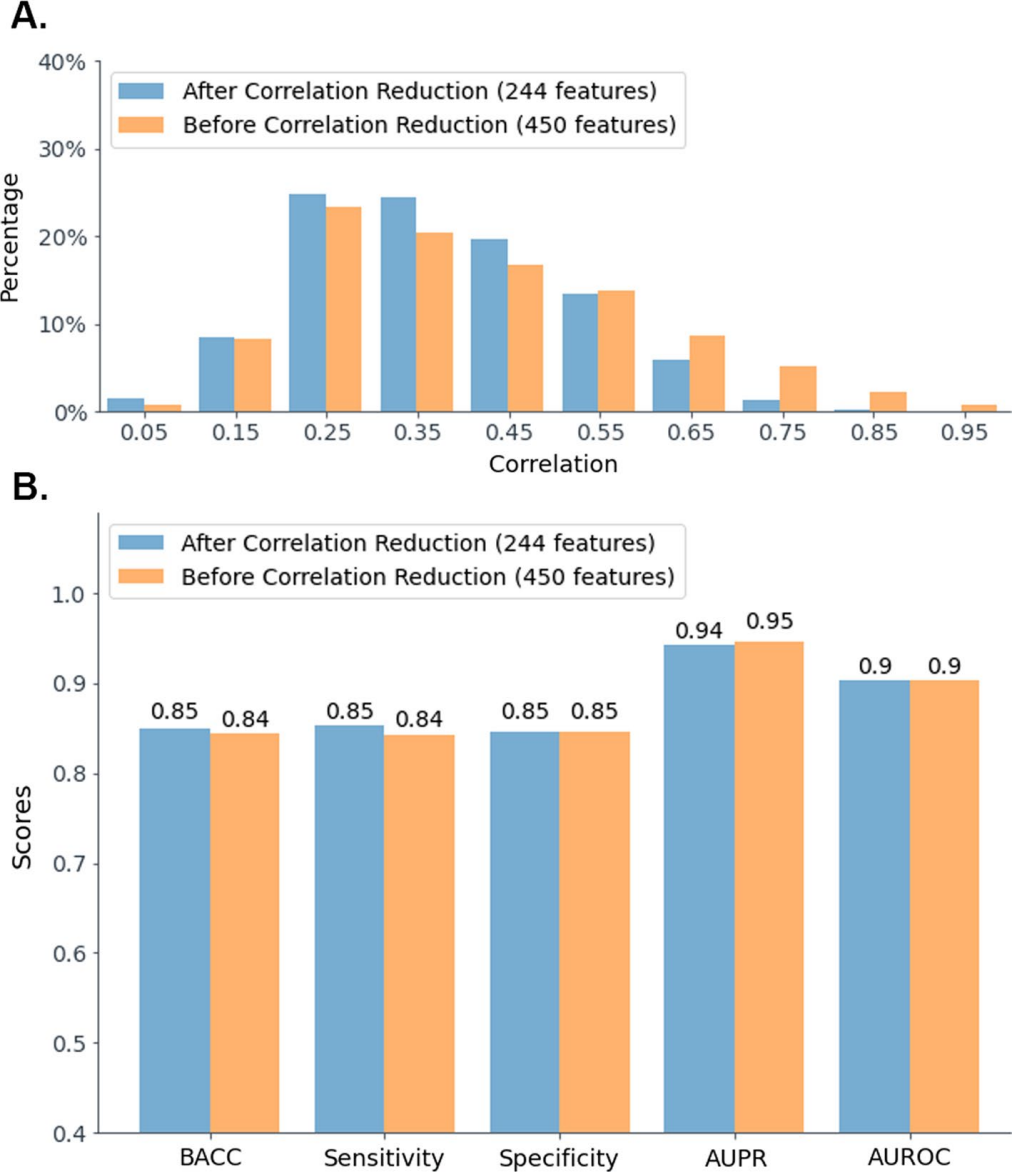
Prediction performance before and after removing highly correlated features from the training set (excluding the validation set). **A** The percentage of pairs of features that have a correlation within a specific range, for different ranges. The labels on the ’x’ axis represent the middle of the relevant range, where each range width is 0.1. **B** Validation set results of the RF classifier trained using the 450 features selected in the first step, and the RF classifier trained using the set of 244 features obtained after removing highly correlated features in the second feature selection step

*Removing Correlated Features.* Functionally related bacterial genes are often organized in gene clusters [37] or operate in other forms of co-regulated genes [38], which leads to a correlation between PGFam features. If a PGFam is part of a gene cluster and achieves a high $\chi ^2$ score, there is a high probability that other PGFams from the same gene cluster will also achieve high $\chi ^2$ scores. Indeed, computation of pairwise correlation scores between the $k=450$ features, which were selected from the training set (excluding the validation set), revealed many correlated pairs (Fig. 3A).

Although an RF-based model can deal successfully with highly correlated features, such correlations can reduce the stability of the model, and may induce a biased variable importance ranking [39]. One way to deal with this issue is to remove highly correlated and redundant features as a part of the feature selection process by clustering correlated features and selecting a representative from each cluster [40–42]. We applied a hierarchical clustering based on a correlation measure between all pairs of features, and then selected from each cluster a feature that has the highest association with the labels of the training set genomes according to the feature’s $\chi ^2$ score (similar to the method used in [42]). The correlation between each pair of features was calculated using the Phi coefficient, a measure of association of two binary variables that range from 0 to 1 [43]. The correlation values were then converted to distance values by subtracting each value from 1. The clustering was performed using the package scipy.cluster.hierarchy of the SciPy library [44], where we chose “average” linkage method.

We selected the number of clusters using parameter *t*, which represents the maximum allowed inter-cluster distance. For each value of *t*, we trained an RF classifier on the training set, and evaluated it on the validation set. Multiple *t* values were examined, starting from $t=0$ (representing 450 clusters with a single feature in each cluster) to $t=0.84$ (representing one cluster that includes all features), increasing by increments of 0.06. We selected the final value $t=0.18$ according to the maximum AUROC score achieved by the classifier (0.903), which was equal to the AUROC score before removing correlated features (Additional file 1: Fig. S1.B). This process resulted in a subset of 244 features with lower correlations between each pair of features (Fig. 3A).

In summary, the corresponding RF classifier, which uses a set of 244 features that were selected from the training set (excluding the validation set) according to parameters $k=450$ and $t=0.18$, achieved sensitivity and specificity values of 0.85, an AUPR value of 0.94, and an AUROC value of 0.9 on the validation set (Fig. 3B). As the genomes in the training set and the validation set belong to distinct species, the obtained classification results may be a good approximation of the expected performance of the classifier on novel species that were not seen during the training process, given that the novel species are not entirely different from the training species.

Note that these validation results are on par with the results achieved by the classifier before removing highly correlated features (Fig. 3B). Therefore, to reduce the risk of overfitting, the smaller set of features is preferred. Applying the feature selection process on the entire training set (training and validation sets combined) using the selected *k* value (450) and the selected *t* value (0.18) resulted in a final set of 250 features.

### Computing the mean decrease impurity for feature importance

During the construction of an RF tree, a subset of features is examined in each split, and the feature that induces the largest decrease of impurity is chosen. In this work, we used the Gini impurity measure for the WSPC RF tree construction, and the Mean Decrease Impurity (MDI) importance measure [27, 45] for computing the importance of each feature (the MDI measure is described in detail in Additional file 1: Section S3.3). To evaluate the feature importance of each PGFam feature in the final set of features, we computed its average MDI value using 100 RF classifiers with different random seeds (seeds 0–99) trained on the combined training and validation sets. The MDI importance measure of a feature of interest was computed through the Scikit-learn python package [29].

## Results

### Prediction performance comparison on the BacPaCS test set

The BacPaCS test set [13], which was used in the two most recent studies [13, 15], is currently the commonly used benchmark for comparing bacterial pathogenicity classifiers. However, our manual inspection of the pathogenicity labels of the genomes in the BacPaCS test set revealed that some of the labels were incorrect, while other labels could not be verified. Therefore, we constructed a correctly labeled BacPaCS test set, which includes 94 genomes (78 HP and 16 NHP) with manually verified labels, denoted Benchmark Test 1 (“BacPaCS test set” Section). Another drawback of the BacPaCS test set, which was observed by Bartoszewicz et al. [15], is that the number of genomes per species is imbalanced. Performance comparison on an imbalanced test set is unfair, since it gives an advantage to a classifier that correctly predicts the labels of species that are over-represented in the test set. For example, 22 out of 78 HP genomes in the BacPaCS test set belong to the species *Bordetella pertussis*. As a consequence, the genomes of this species affect 28% of the sensitivity score. Hence, in order to reduce species redundancy, we generated a balanced version of Benchmark Test 1, denoted Benchmark Test 2, which consists of exactly one randomly selected genome per species (25 HP and 15 NHP, “BacPaCS test set” Section). Both Benchmark Test 2 and Benchmark Test 1 were used for the performance comparison. We compared the predictions of WSPC with the predictions of the following extant pathogenicity classifiers: the protein-content-based classifiers PathogenFinder [12] and BacPaCS [13], and the read-based classifiers PaPrBaG [14] and DeePaC [15]. The binary predictions of all classifiers were compared using the measures of sensitivity, specificity, and BACC (see “Generating and evaluating a classification model” Section).

**Fig. 4:**
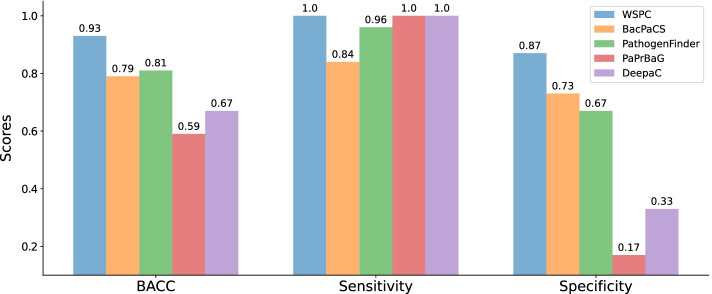
Classification performance comparison between WSPC and extant classifiers on a subset of the BacPaCS test set, containing one genome per species (Benchmark Test 2)

WSPC outperformed extant classifiers on both Benchmark Test 1 (Additional file 1: Fig. S2) and Benchmark Test 2 (Fig. 4), achieving a greater BACC value than any of the other classifiers. As Benchmark Test 2 contains almost all NHP genomes from Benchmark Test 1, and only 25 out of 78 HP genomes, it is mainly the sensitivity scores that were expected to differ between the two benchmark test sets. For these benchmarks, the main differences were obtained for BacPaCS and PathogenFinder, where BacPaCS achieved greater sensitivity than PathogenFinder on Benchmark Test 1, and vice versa on Benchmark Test 2. The sensitivity scores of the other classifiers, including WSPC, were similar across the two benchmarks. The BACC obtained by WSPC on Benchmark Test 1 was 8% higher than the BACC achieved by the second-ranked classifier BacPaCS, and the BACC obtained by WSPC on Benchmark Test 2 was 12% higher than the BACC achieved by the second-ranked classifier PathogenFinder. Observing the results on Benchmark Test 2, we note that while all classifiers obtained high sensitivity scores ($\ge$ 0.84), which represent correctly classified HPs, on this data, their specificity scores, which represent correctly classified NHPs, were at least 10% lower than their sensitivity scores. This difference could be due to the imbalance between the HP and NHP genomes in the training sets of some of these classifiers (BacPaCS—5:1, PaPrBaG and DeePaC—7:1). In particular, the read-based classifiers achieved perfect sensitivity scores, but very low specificity scores, on this benchmark.

### Prediction evaluation on the WSPC test set

Following the training procedure, WSPC was evaluated on a test set consisting of genomes that were inserted into the PATRIC database later than the training set genomes (“WSPC test set” Section). The test set is relatively large. It includes 204 genomes (102 HP and 102 NHP) from 93 genera, where each genome (strain) belongs to a different species. Some of the strains belong to species that are also included in the training set, while other strains belong to species with no representative in the training set, denoted *novel species*. To estimate the results of the classifier on novel species, we also evaluated the classifier on a subset of the test set that includes 63 bacterial genomes (32 HP and 31 NHP) that belong to novel species. Note that since the phylogenetic dependency between species is not completely reduced, we expect improved prediction performance on novel species that are more closely related to the species present in the training set, rather than on more distant novel species.

**Fig. 5:**
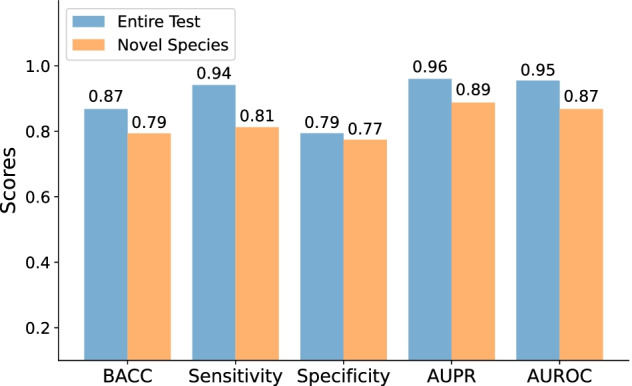
WSPC classification performance on the entire test set and on a subset of it containing only novel species

**Fig. 6:**
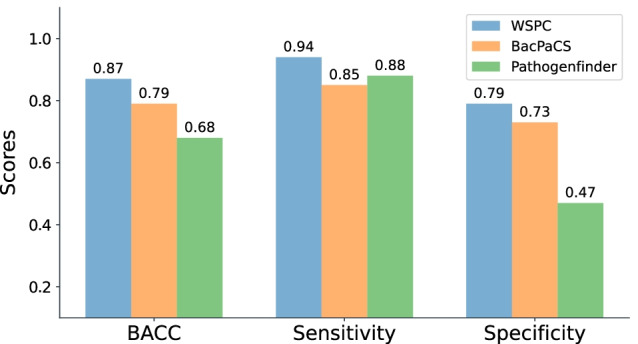
Classification performance comparison of WSPC, BacPaCS, and PathogenFinder (protein-content-based classifiers) on the WSPC test set

WSPC achieved high scores for each of the evaluated metrics on the entire test set, correctly predicting the pathogenicity label of 96 out of 102 HP bacteria, and 81 out of 102 NHP bacteria (Fig. 5). As expected, the evaluation results of the classifier on the novel species subset were lower than on the entire test, implying that it is more difficult to predict the pathogenicity of novel species (Fig. 5). Nevertheless, WSPC correctly predicted 77% of the HPs and 81% of the NHPs in this subset of novel species. In addition, we evaluated the performance of WSPC and two extant protein-content-based classifiers, PathogenFinder and BacPaCS, on the WSPC test set (Fig. 6). WSPC achieved higher sensitivity and specificity scores, which further validates the ability of the WSPC classifier to correctly predict the pathogenicity of a large and diverse group of genomes.

### WSPC model interpretation

We assessed the importance of the 250 PGFams that serve as features in WSPC using the MDI importance measure (“Computing the mean decrease impurity for feature importance” Section). The MDI measure is widely used for assessing the ability of an RF input feature to predict the target variable [27, 45]. The higher the MDI of a PGFam feature, the higher the importance of this PGFam in splitting the training set into the different pathogenicity classes.

#### PGFams related to pathogenic bacteria

Table 1 presents the top 15 PGFams, which are more abundant in HP versus NHP genomes (HP PGFams), sorted according to their average MDI ranks. These PGFams, which represent widely spread genes that are common to many pathogenic species and genera, probably serve as important features in our model for the following two reasons. First, PGFams that are present in a large number of HP genomes and in a small number of NHP genomes have high $\chi ^2$ association values with the pathogenicity label and therefore are more likely to be selected in the first step of the feature selection process (“Feature selection of the WSPC classifier” section). The 450 PGFams with the highest $\chi ^2$ scores, which were selected in the first step of the feature selection process, had very high $\chi ^2$ values ($\ge$ 68.4, corresponding to $\chi ^2$ test *p* values $\le 10^{-16}$), where selected HP PGFams appear in at least 139 of 428 HP genomes. Second, PGFams that are present in a large number of HP genomes and in a small number of NHP genomes yield a high decrease of Gini impurity when selected for a tree node in the RF classifier. Thus, their importance in separating the training set into the two different pathogenicity classes is high (“Computing the mean decrease impurity for feature importance” Section).

**Table 1:** The top HP PGFams that serve as features of WSPC according to their average MDI rank, along with the number of HP and NHP genomes in the training set that contain the respective PGFams

	PGFam ID	Gene function	General function	MDI (SD$^1$)	HPs	NHPs	P-ratio$^2$	# Genera$^3$
1	04139053	Uroporphyrinogen III decarboxylase	Energy production	0.038 (0.012)	362	27	6.47	109
2	01915472	Dihydrolipoamide acetyltransferase component of PDC$^{*}$	Aerobic respiration	0.035 (0.01)	385	48	3.93	120
3	07629184	Cytosol aminopeptidase PepA	Regulation	0.03 (0.009)	366	39	4.58	115
4	07157721	Heme O synthase, protoheme IX farnesyltransferase, COX10-CtaB	Aerobic respiration	0.022 (0.007)	312	14	10.41	89
5	00022550	Molybdopterin synthase catalytic subunit MoaE	Respiration, energy conversion	0.013 (0.005)	303	17	8.42	89
6	01033770	Class 2 Dihydroorotate dehydrogenase (DHODase)	Amino acid biosynthesis	0.011 (0.005)	333	35	4.63	99
7	00006100	tRNA-modifying protein YgfZ	Synthesis and repair	0.011 (0.006)	305	17	8.48	93
8	07941512	23S rRNA methyltransferase	Methylation	0.01 (0.003)	324	37	4.27	89
9	00405499	YpfJ protein, zinc metalloprotease superfamily	Protein cleavage	0.009 (0.003)	273	13	9.76	87
10	06757295	Threonine dehydratase	Amino acid biosynthesis	0.008 (0.004)	323	34	4.62	95
11	08199696	Glutathione reductase	Stress tolerance	0.008 (0.002)	220	12	8.48	66
12	03081665	Cell division integral membrane protein, YggT	Stress tolerance	0.007 (0.003)	352	56	3.09	109
13	07854425	Superoxide dismutase [Cu–Zn] precursor	Stress tolerance	0.006 (0.001)	252	21	5.74	75
14	01668012	Sulfur carrier protein FdhD	Stress tolerance	0.006 (0.003)	300	26	5.56	98
15	01147190	Deoxyribodipyrimidine photolyase	DNA repair	0.006 (0.003)	281	17	7.82	88

The average MDI rank of a PGFam is the average value of the feature’s MDI values computed using 100 random forest classifiers with different random seeds trained on the training set. $^1$Standard Deviation. $^2$The ratio between the proportion of HPs with the corresponding PGFam and the proportion of NHPs with the corresponding PGFam. To avoid zero division, add-one smoothing was performed. $^3$ The number of different genera to which the genomes that contain the respective PGFams belong. PDC: Pyruvate dehydrogenase complex

Looking into the functions of the PGFams in Table 1 revealed that most of them represent genes that allow the bacterial pathogen to survive and grow during infection, rather than genes that are directly involved in causing host damage. This finding suggests that virulence genes are probably common among pathogens that infect similar tissues and environmental niches and much less among pathogens in general. For example, various pathogens that infect the lungs would likely utilize similar virulence genes to colonize this niche; however, these will differ from the virulence genes required for intestinal pathogens. Therefore, in our dataset, which contains one representative per species, the genes that had the highest potential to separate between HPs and NHPs were related to rapid metabolism and tolerance to stress conditions. These genes are required for the pathogenic lifestyle of bacteria, in general, as they supply nutrients during colonization, improve competition with other microbes, and provide a proper micro-environment [5], and therefore their relative weight in the model is high. More specifically, all PGFams in Table 1 are involved in the processes of respiration and energy production, DNA repair, amino acid metabolism, heme biosynthesis, and stress tolerance. A detailed description of each of the PGFams in Table 1 can be found in Additional file 1: Section S5.1.

Interestingly, 4 out of the 15 PGFams in Table 1 (PGFams 1, 2, 4, and 5) have a role in bacterial respiration and energy production. Many important human pathogens are facultative anaerobes, i.e., bacteria that can grow in both the presence or absence of oxygen, and therefore can adapt to a vast array of oxygen concentrations [46]. These facultative anaerobes include all major pathogens of the human lower gastrointestinal tract (enteropathogens). These pathogens are exposed to fluctuating oxygen conditions, and multiple respiratory pathways contribute to their survival in the intestine [46]. Moreover, several enteropathogens induce intestinal inflammation through their virulence genes. The inflamed intestine has a higher concentration of oxygen due to high blood flow and hemoglobin levels [47]. This aerobic environment gives an advantage to bacteria that can utilize oxygen for growth, including pathogens such as *Salmonella*, *Escherichia coli*, *Klebsiella*, and *Shigella*. In contrast, the resident microbiota rely mainly on anaerobic fermentation of carbohydrates [48].

A critical factor for bacterial survival in any environment is their ability to sense and respond properly to stress factors. In particular, human pathogens must survive under two entirely different conditions: the environment, and within their hosts [49]. This may explain the high percentage (4 out of 15 PGFams in Table 1—PGFams 12–14) of genes that are involved in conferring tolerance to different environmental stresses: oxidative, osmostic, UV, and low pH, which the bacterium can get exposed to during its host colonization.

#### PGFams related to non-pathogenic bacteria

Additional file 1: Table S1 shows the top 15 PGFams, which are enriched in NHP versus HP genomes (NHP PGFams), sorted according to their average MDI ranks. These PGFams represent proteins participating in processes such as nucleotide metabolism, RNA processing, fermentation of L-glutamate, and carbon metabolism. These PGFams may be common to NHP genomes due their prevalence in commensal gut bacteria. For example, the intestinal microbiome is dominated by anaerobes [50], and therefore it is not surprising that the electron transport complex protein RnfB can be found in the top NHP PGFams, as this protein is usually found in anaerobic bacteria [51]. Another example is found in two of the PGFams in Additional file 1: Table S1 that encode rubrerythrin variants, a protein with an unknown physiological role that was found to be abundant in gut bacteria [52].

## Conclusions

In this work, we developed WSPC, a novel machine-learning-based approach for classifying a bacterial genome as pathogenic or non-pathogenic to humans based on its protein content and without prior knowledge of its taxonomic association. WSPC uses protein families as features, and it overcomes the running time overhead of clustering genes into protein families by using the readily available PATRIC PGFams. The resulting classifier is highly accurate, outperforming existing read-based and protein-content-based classifiers on a benchmark test containing 40 species of 30 genera.

WSPC is publicly available and can be used for the pathogenicity prediction of existing or novel bacterial species. Furthermore, the analysis of genes that are highest ranking in terms of their importance for the classification by WSPC suggests that when examining a broad range of pathogens, the most important genes are linked to rapid metabolism and high tolerance to various stress conditions, rather than to classical virulence genes. These results propose that future interpretation of the results of a pathogenicity classifier should be done in consideration of the tissue or the infected organ. Such an interpretation is likely to highlight specific virulence genes, which are essential for pathogens that colonize a specific niche/environment.

For future works targeting the specific objective of seeking virulence genes, rather than a general pathogenicity classifier, one could consider narrowing the width of the bacterial genome sampling to niche-specific or taxa-specific datasets. In addition, the two-step feature selection approach we utilized in our model, which leads to selecting the most discriminative features and then removing correlated features, may suffer from low robustness. Therefore, for future work, we suggest testing other feature selection approaches, such as Boruta [53].

In this study, the bias due to the phylogenetic dependency between genomes was removed by selecting one genome per species. Evidently, this method yielded a competitive pathogenicity classifier where the top-ranking features are gene families that are common to many bacterial genera. However, although this selection process removes redundancy, it also removes a large amount of the training data. Therefore, future works should consider using more sophisticated methods for redundancy removal. For example, redundancy may be removed by adjusting the weight of each sampled genome in the training set by calculating its phylogenetic similarity to other samples in the set.

We hope to see our proposed method applied to the prediction of other bacterial phenotypes. For example, predicting the environmental niche from which the bacterial strain was collected (e.g., host type, soil, water). Analyzing the features of the resulting models may reveal protein families involved in bacterial adaptation to these niches.

## Supplementary Information

**Additional file 1.** Supplementary text, figures and tables.

## Data Availability

The code for WSPC as well as all the data curated through this study are publicly available on https://github.com/shakedna1/wspc_rep.

## Abbreviations

WSPC: Wide scope pathogenicity classifier
HP: Human pathogen
NHP: Non-human pathogen
EHP: Exclusive human pathogen
ENHP: Exclusive non-human pathogen
OHP: Opportunistic human pathogen
ONHP: Opportunistic non-human pathogen
RF: Random forest
BACC: Balanced accuracy
AUROC: Area under the receiver operation characteristic curve
AUPR: Area under the precision recall curve
MDI: Mean decrease impurity
PGFAM: PATRIC global protein family
