# Supplementary material for: Predicting the pathogenicity of bacterial genomes using widely spread protein families
Source: BMC Bioinformatics. 2022 Jun 24;23:253. doi: 10.1186/s12859-022-04777-w (PMC9233384; doi:10.1186/s12859-022-04777-w)
Supplement: Supplementary file 1 — Additional file 1. Supplementary text, figures and tables. [file 12859_2022_4777_MOESM1_ESM.pdf]

Supplementary Materials

Shaked Naor-Hoffmann† [1], Dina Svetlitsky†[1], Neta Sal-Man[2], Yaron Orenstein[3], and Michal Ziv-Ukelson[1]

[1]Department of Computer Science, Ben Gurion University of the Negev, Be'er Sheva, Israel
[2]The Shraga Segal Department of Microbiology, Immunology and Genetics, Faculty of Health Sciences, Ben-Gurion University of the Negev, Be'er-Sheva, Israel
[3]School of Electrical and Computer Engineering, Ben-Gurion University of the Negev, Be'er Sheva, Israel

Contents

S1 Previous Works 3

S2 Dataset Creation Process 4
S2.1 Labeling Method 4
S2.2 Manual Inspection of the WSPC Test Set Genomes 5
S2.3 Manual Verification of the BacPaCS Test Set Genomes 6

S3 Methods 7
S3.1 Evaluation Metrics 7
S3.2 Feature Selection of the WSPC Classifier - Parameter Tuning 8
S3.3 Mean Decrease Impurity Measure Computation for Feature Importance 9

S4 Classification Performance Comparison on Benchmark Test 1 9

S5 Biological Interpretation 9
S5.1 A Detailed Description of Each of the Top HP PGFams 9
S5.2 Top NHP Features 12

S6 BacPaCS Test Genomes 14

S7 WSPC Test Genomes 16

List of Figures

S1 Selecting the most discriminative features and removing highly correlated features . . 8
S2 Classification performance comparison between WSPC and extant classifiers on 94 out of 100 genomes of the original BacPaCS test set 9

†S.N.H and D.S. are equal contributors

List of Tables

S1 Top NHP PGFams of the WSPC classifier 13
S2 A list of genomes included in the BacPaCS test set 16
S3 A list of genomes included in the WSPC test set 23

S1 Previous Works

In this section, we review previous computational methods for pathogenicity classification. The methods can be divided into two main categories: Read-based methods and protein-content-based methods.

Read-based Methods. Read-based classification approaches use short genomic reads as raw input, and hence do not depend on assembled genomes or on coding sequences. Several tools were proposed for the detection of pathogens in metagenomic reads based on mapping the reads to a species, genus, or phylum of reference genomes, and/or based on their sequence composition homology [1]. However, these methods were mainly designed for taxonomic assignment using known species that are present in a reference database, rather than for pathogenicity predictions. In addition to the taxonomy-dependent methods, several taxonomy-agnostic read-based methods were designed specifically for pathogenicity predictions [2, 3]. In 2017, Deneke *et al.* published PaPrBaG [2], an RF approach that uses classification features of two types: k-mer occurrence-based features and peptide features. PaPrBaG assigns pathogenicity prediction to each read in a genome sample, and computes the final prediction of the genome by averaging over all of its read-based prediction probabilities. A more recent read-based taxonomy-agnostic classification tool is DeePaC [3], which applies reverse-complement convolutional and recurrent neural networks to the classification task.

Protein-content-based Methods. Protein-content-based methods are classification approaches that require the availability of assembled and annotated genomes as they characterize a bacterial genome by the presence or absence of protein family members [4, 5, 6, 7].

Iraola *et al.* [5] constructed an SVM-based model to classify a bacteria, based on its genome, as a human pathogen or a non-pathogen using protein families annotated as virulence genes. Thus, their method has the disadvantage of not taking into account other protein families that could be associated with either HP or NHP phenotypes. Other protein-content-based tools constructed new protein families instead of relying on existing ones [4, 6, 7]. Andreatta *et al.* [4] developed a method to predict human pathogenicity in γ-Proteobacteria. They clustered new protein families using BLAST, and identified protein families that distinguish between the two phenotypes by their enrichment in either pathogens or non-pathogens. The pathogenicity of a new bacterium was determined by the presence or absence of proteins that belong to these distinguishing protein families in its genome. Building on the former method, Cosentino *et al.* [6] developed PathogenFinder, a similar method extended to handle a variety of bacterial taxonomic groups. In order to reduce the method's runtime, CD-HIT was used instead of BLAST in the protein family clustering step of the algorithm, which took four weeks for a dataset of 885 genomes.

Recently, Barash *et al.* [7] developed BacPaCS, a sparse-SVM-based method for bacterial pathogenicity classification. Barash *et al.* also used CD-HIT for the construction of protein families, but in order to reduce the runtime of the clustering phase and to scale up the method to larger training data, only the top 10% of the longest gene sequences in the training set were used for the construction of protein families. Although this selection reduced the estimated runtime from 8 months to 12 days (for 21,155 genomes), the clustering step still remained the most computationally expensive step of the

training in all the aforementioned methods. Furthermore, when examining the top-scoring features of the BacPaCS classifier, as reported in the paper, it is evident that many of the top-ranking features appear in only a few genomes, which may hamper the classifier's ability to contribute to the general understanding of pathogenic lifestyle, or to predict the pathogenicity of novel bacterial species.

The number of available sequenced strains per bacterial species varies greatly, mainly due to a bias towards pathogen studies [2]. Thus, the distribution of strains per species in a given database may not represent their distribution in the real world or in future applications. Consequently, this may result in a training dataset and a classifier that are biased towards specific species. Existing read-based methods addressed this issue by selecting one strain per species for their training sets [2, 3]. However, previous protein-content-based methods [4, 6, 7] did not properly address this normalization issue, and thus their generality may be at fault.

S2 Dataset Creation Process

S2.1 Labeling Method

To identify the HP and NHP bacteria in our dataset, we initially followed Barash *et al.* [7] annotation-based pathogenicity classification method, as described below:

1. Genomes are labeled as HP if they satisfy any of the following criteria:
   (a) The 'Disease' field is not empty, and does not contain a commensal term, as defined below.
   (b) One of the fields 'Isolation Source', 'Host Health', or 'Comments' includes an HP term. In addition, the same fields cannot include any of the NHP terms (the terms used for HP and NHP are presented below).
   (c) A genome was manually verified as HP, by reviewing it in the literature.
2. Excluding the generated HP list, genomes are labeled as NHP if they satisfy any of the following criteria:
   (a) One of the fields 'Isolation Source', 'Host Health', or 'Comments' includes an NHP term.
   (b) One of the fields 'Isolation Source', 'Host Health', or 'Comments' includes a weaker NHP term.
3. The following term lists were used for the criteria above:
   - HP terms: virulence, disease, superbug, patient, diarrhea, waterborne, foodborne, toxin, clinical, intensive, outbreak, infection, pathogen, water borne, food borne.
   - NHP terms: healthy, probiotic, commensal.
   - Weaker NHP terms: 'comparative', 'reference'.
   - Commensal terms: 'healthy', 'Healthy', 'Commensal', 'Commensal (plant)', 'Periodontally healthy'.

A manual examination of a random sample from the training set revealed that some of the genomes were mislabeled. These labeling mistakes were caused by using keywords that can both describe HP and NHP genomes. In addition, a manual examination of a random sample from the group of genomes that were labeled as inconclusive revealed that some genomes could be labeled by utilizing additional keywords and fields. Therefore, the following modifications to the annotation process were made:

- 'Other Clinical' and 'Isolation Comments' were added to the list of relevant fields in 1(b) and 2(a).
- The word 'intensive' was removed from the HP terms.
- The words 'microbiome' and 'microbiota' were added to the NHP terms.
- All the weaker NHP terms were removed.

This rule-based labeling process correctly identifies HP genomes due to the pathogenic sample collection process. First, the PATRIC database utilizes information from standard medical practice in which clinical samples are taken from diseased individuals based on the illness symptoms. Thus, the decision of which sample to take (e.g., stool, urine, blood, nasal swab, sputum swab) is illness-related. Second, the samples are processed based on the suspected pathogens, i.e., cultured using specific conditions that will allow the growth of the suspected pathogen. Therefore, in the case of an identification of a bacterial strain, it can be medically referred to as the infectious agent.

S2.2 Manual Inspection of the WSPC Test Set Genomes

We manually inspected all the genomes in the WSPC test set (Section 2.1.3, main text) to ensure that their labels are correct by reviewing the associated PATRIC metadata. We verified a genome as HP if the isolation source is a diseased individual, and as NHP if the isolation source is a healthy individual or a probiotic supplement. Additionally, we performed a literature search to confirm the corresponding label.

The following strains could not be verified as HP or NHP:

1. The PATRIC metadata of a strain belonging to the species *Escherichia marmotae* (Genome ID: 1499973.23) indicated that it was collected as part of a study that sequenced metagenomes from urinary tract patients as well as from a control group. There was no indication to which of the two groups this strain belonged, and it was thus removed from the test set.
2. The PATRIC metadata of a strain belonging to an unclassified species of the genus *Starkeya* (Genome ID: 2666134.3) indicated that the host health status is diseased. However, this genus includes soil bacteria [8], and we were not able to find a study suggesting that *Starkeya* species could be pathogenic. Therefore, this genome was removed from the test set.

In addition, two strains were mislabeled by the automatic annotation. A strain of the species *Bacillus clausii* was labeled HP because of the keyword "disease" in the "Comments" field. The full sentence "Genome analysis of Bacillus clausii B619/R for evaluation of its health promoting and disease

preventing properties" indicates that it is a commensal probiotic bacterium, which is also supported by the literature [9]. Therefore, its label was corrected to NHP. A strain of the unclassified species *Clostridium sp. C5-48* was labeled as HP because of the keyword "patient" in the isolation source field. However, this strain was collected from the feces of an alcoholic patient to study his metagenomic population in the colon, and a different database indicates that this strain is commensal [10]. Therefore, its label was corrected to NHP.

S2.3 Manual Verification of the BacPaCS Test Set Genomes

We manually curated the 100 genomes included in the BacPaCS test set using the metadata associated with each genome and the literature. We verified a genome label as HP if it was isolated from a diseased host (based on the PARTIC database entry), and if there was also evidence in the literature that the corresponding species or strain is pathogenic. We verified a genome label as NHP if it was isolated from a healthy host, and if the corresponding species or strain was also described in the literature as commensal or probiotic.

A list of all the genomes included in the original BacPaCS test set along with their verified labels, references to relevant studies, as well as an indication of whether each genome was included in Benchmark Test 2, is given in Table S2. In what follows, we summarize the modifications we made following the verification process.

1. A total of 18 strains belonging to the species *Pseudomonas aeruginosa*, *Acinetobacter nosocomialis*, *Streptococcus sp. NPS 3089*, *Acinetobacter baumannii*, *Escherichia coli*, and *Enterococcus faecium* were originally labeled as NHP, but were verified by us as HP as these strains were isolated from clinical samples or described in the literature as well-known pathogenic strains. This may explain the discrepancy between the pathogenicity annotations detected by Bartosezewitch *et al.* [3].

2. The labels of another six strains belonging to the species *Fusobacterium nucleatum*, *Fusobacterium periodonticum* and *Rothia aeria*, could not be verified by us as neither HP nor NHP:

   - The species *Fusobacterium nucleatum* included one strain that was collected from subgingival dental plaque (which may be an initiating factor in periodontal diseases [11]) and the species *Fusobacterium periodonticum* included four strains that were collected from the tongue or from dental plaque. For these genomes there was no indication in the corresponding metadata whether the hosts carried a periodontal disease. The species *Fusobacterium nucleatum* and *Fusobacterium periodonticum* are associated with a wide spectrum of human periodontal diseases [12, 13]. Therefore, these strains could not be reliably labeled and were removed from the test set.

   - The species *Rothia aeria* included one strain, which was isolated from an air sample of the living environment in the Mir space station [14]. *Rothia aeria* is described as an opportunistic periodontal pathogen that causes infections of immunocompromised patients and neonates, but its virulent features remain uncertain [14]. As it was collected from the

environment, it is not clear whether this specific strain can cause disease, and therefore it was removed from the test set.

S3 Methods

S3.1 Evaluation Metrics

Some of the test sets used in this paper consist of more HP than NHP labeled genomes. Using a regular accuracy metric (the proportion of correct predictions in the test set) may result in misleading classification evaluation due to the imbalance between the two classes. Therefore, we used Sensitivity (true positive rate), Specificity (true negative rate), and Balanced Accuracy (BACC), which denotes the mean of sensitivity and specificity [15]. In addition, for ranking evaluation of WSPC, we used the areas under the precision recall (AUPR) [16], and the receiver operation characteristic (AUROC) [17] curves. Since AUROC considers the ranking of all predictions while accuracy only considers a single prediction threshold (i.e., 0.5) [18], we used AUROC for the feature selection parameter tuning (Section S3.2 and Section 2.4.3 in the main text). Note that in the case of a highly imbalanced dataset, AUPR is more informative than AUROC [19]. However, since there is only a slight imbalance between the classes of the validation set (ratio of 2:1 HP to NHP), and as AUROC is more commonly used than AUPR in the general case, we opted to use AUROC for the purpose of parameter tuning.

S3.2 Feature Selection of the WSPC Classifier - Parameter Tuning

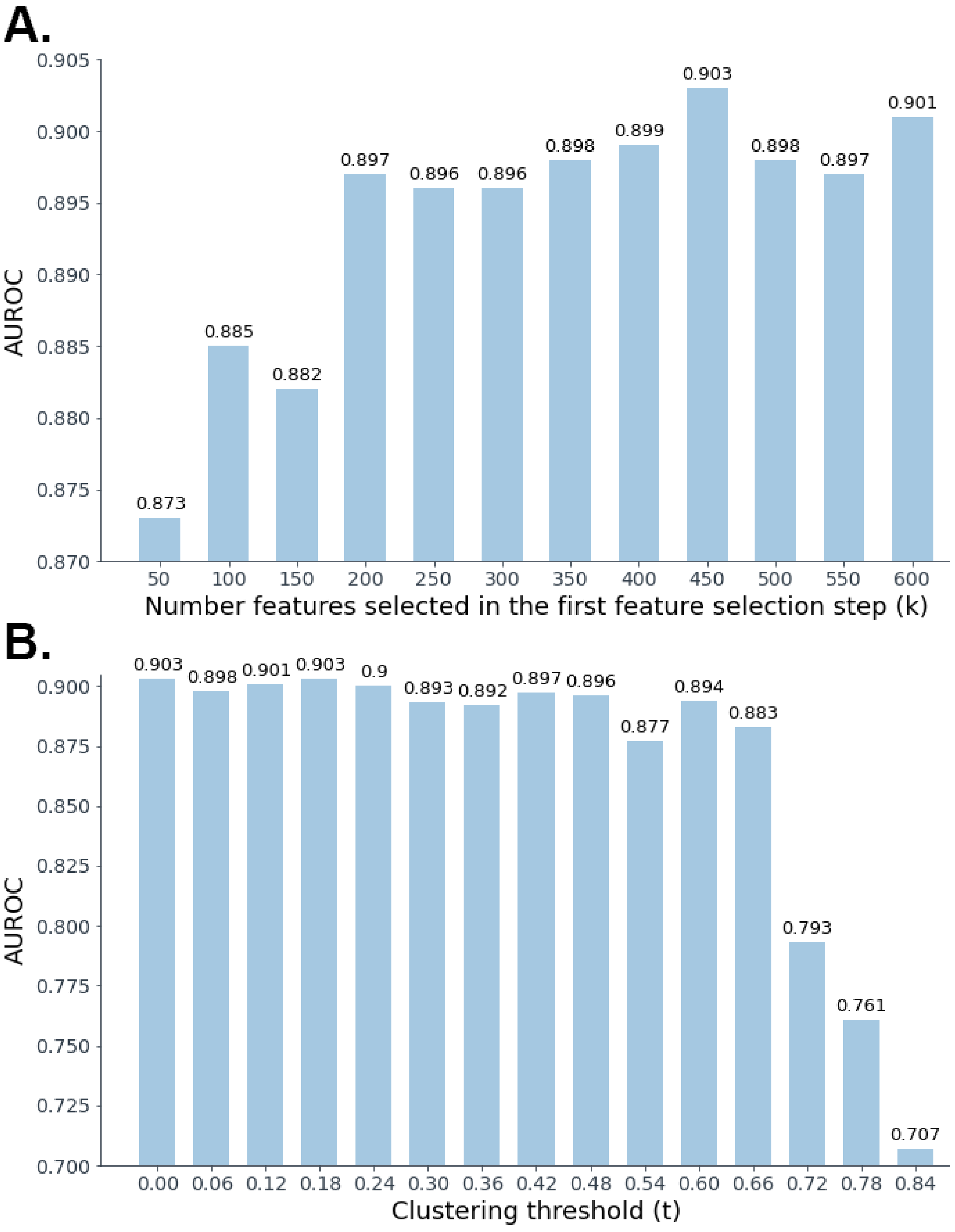

Figure S1: Performance evaluation of the RF classifier using different values for the k and t parameters of the two-step feature selection process (Section 2.4.3, main text). We trained on the training set, and evaluated on the validation set. **A.** AUROC values achieved by the classifier, as a function of k - the number of features selected in the first feature selection step (based on their χ^2 scores, without correlation reduction). The maximum value was obtained for $k = 450$. **B.** AUROC values achieved by the classifier using different subsets of the 450 features selected in the first feature selection step, as a function of t - the clustering threshold. The maximum value was obtained for $t = 0.18$ resulting in a subset of 244 features, and is equal to the AUROC score value obtained before removing correlated features ($t = 0$).

S3.3 Mean Decrease Impurity Measure Computation for Feature Importance

The Mean Decrease Impurity (MDI) importance measure [20, 21] of a feature of interest is computed by the Scikit-learn python package [22] as follows:

1. For each tree in the forest, the total decrease in Gini impurity in all the splits that use this feature, weighted by the proportion of samples reaching that split, is computed.

2. The resulting value in each tree is averaged over all trees in the forest

Consequently, the MDI values for all features sum to one. Thus, an “important” feature is often selected for tree splits and yields a high decrease of Gini impurity, leading to a high MDI. To evaluate the feature importance of each PGFam feature in the final set of features, we computed its average MDI value using 100 RF classifiers with different random seeds (seeds 0-99) trained on the combined training and validation sets.

S4 Classification Performance Comparison on Benchmark Test 1

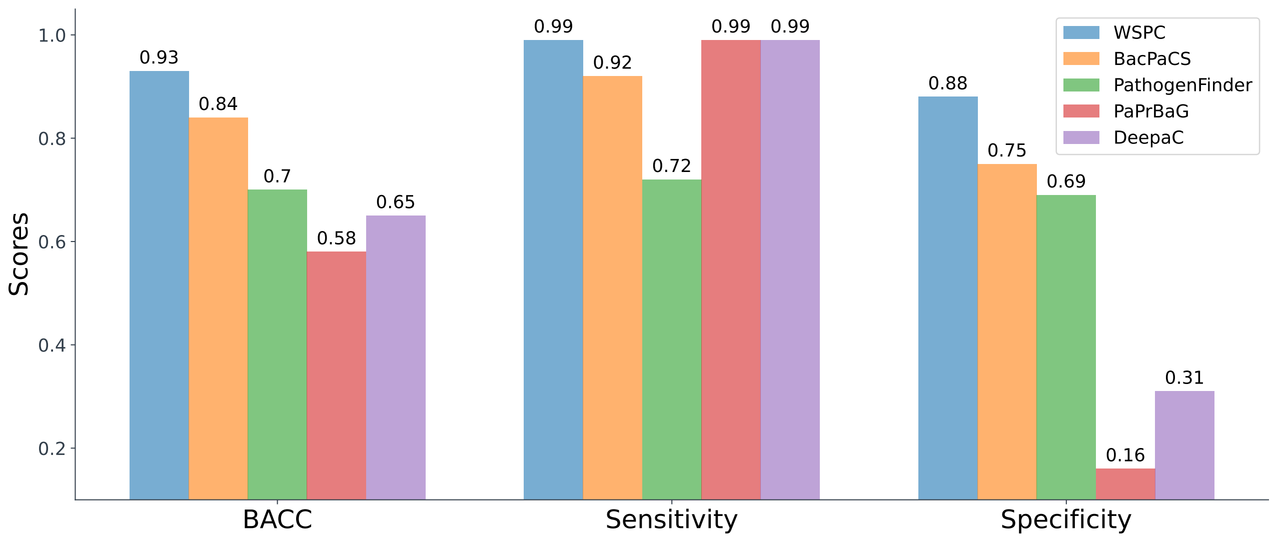

Figure S2: Classification performance comparison between WSPC and extant classifiers on 94 out of 100 genomes of the original BacPaCS test set with manually verified labels (Benchmark Test 1, Section 2.2 in the main text).

S5 Biological Interpretation

S5.1 A Detailed Description of Each of the Top HP PGFams

As a part of the feature selection process, we performed clustering based on a correlation measure between all pairs of features (i.e., genes), and then selected a PGFam representative from each cluster. Interestingly, 14 out of the 15 PGFams that yielded highest MDI scores in the HP category (Table 1 in the main text), each belong to a ”singleton” cluster that contains a single member.

In what follows, we describe the biological function of each of the 15 PGFams in Table 1 from the main text. In addition, we provide a list of PGFams that belong to the same cluster as the PGFam ranked seventh (tRNA-modifying protein YgfZ).

1. Uroporphyrinogen III decarboxylase (EC 4.1.1.37) is an enzyme that catalyzes the fifth step in heme biosynthesis [23]. Heme is essential to the function of hemoproteins, which are involved in processes such as energy generation by the electron transport chain and detoxification of host immune effectors. Both heme acquisition and synthesis are important for pathogenesis [24]. Uroporphyrinogen decarboxylase was found to be important for the survival of *Actinobacillus pleuropneumoniae*, a pathogen swine, during infection [25].

   Other PGFams in the corresponding cluster: None.

2. Dihydrolipoamide acetyltransferase (EC 2.3.1.12) is an enzyme component of the multienzyme pyruvate dehydrogenase complex, which has an important role in aerobic respiration pathways [26]. It has been shown that Dihydrolipoamide acyltransferase is critical for Mycobacterium tuberculosis pathogenesis [27], and for the colonization of *Vibrio cholerae* [28].

   Other PGFams in the corresponding cluster: None.

3. Cytosol aminopeptidase PepA (EC 3.4.11.1). Aminopeptidases are enzymes that catalyze the cleavage of amino acids and are active in several essential cellular processes [29]. PepA transcriptionally regulates the carB gene, which playes multiple roles in the pathogenicity of Xanthomonas citri [30]. In addition, PepA mediates pH regulation of virulence genes in Vibrio cholerae [31].

   Other PGFams in the corresponding cluster: None.

4. Protoheme IX farnesyltransferase is an enzyme involved in catalysing the conversion of heme B to heme O, encoded by the gene ctaB. Heme O is incorporated into the electron transport chain as an electron acceptor, facilitating aerobic respiration and energy production [32]. The deletion of ctaB was observed to cause attenuated growth and virulence of *Staphylococcus aureus* [33], and it was also observed that this gene plays a critical role in the ability of *S. aureus* to secrete cytolytic toxins [34].

   Other PGFams in the corresponding cluster: None.

5. Molybdopterin synthase catalytic subunit MoaE (EC 2.8.1.12), is involved in the molybdenum cofactor (MoCo) biosynthetic pathway [35]. Bacteria posses MoCo-dependent enzymes that catalyze redox reactions associated with bacterial respiration and energy conversion processes. These enzymes have been linked to virulence in a variety of bacteria [36]. The prevalence of MoCo-dependent enzymes in key bacterial pathogens, paired with the mounting evidence of their central roles in bacterial fitness during infection, suggest that they could be important future drug targets [37].

   Other PGFams in the corresponding cluster: None.

6. Class 2 dihydroorotate dehydrogenase (DHODase) participates in the pyrimidine de novo biosynthesis pathway. The pyrimidine synthetic pathway plays essential roles in the pathogenesis and antibiotic resistance of P. aeruginosa and E. coli, and in the survival of the pathogen H. pylori.

   Other PGFams in the corresponding cluster: None.

7. Uncharacterized tRNA-modifying protein YgfZ, participates in the synthesis and repair of iron-sulfur (Fe-S) clusters. A mutation in YgfZ causes growth defects in Escherichia coli, particularly under oxidative stress, and lowers the activities several Fe-S enzymes [38].

   Other PGFams in the corresponding cluster:

   PGF_00024322 NAD(P) transhydrogenase subunit beta (EC 1.6.1.2)
   PGF_00045982 Pyruvate dehydrogenase E1 component (EC 1.2.4.1)
   PGF_00416576 3'-to-5' oligoribonuclease (orn)
   PGF_01053024 Glutamine synthetase adenylyl-L-tyrosine phosphorylase (EC 2.7.7.89) / Glutamate-ammonia-ligase adenylyltransferase (EC 2.7.7.42)
   PGF_03000099 Ribonuclease Y
   PGF_04792416 LSU ribosomal protein L32p @ LSU ribosomal protein L32p, zinc-independent
   PGF_05562713 [Protein-PII] uridylyltransferase (EC 2.7.7.59) / [Protein-PII]-UMP uridylyl-removing enzyme
   PGF_10461681 Ribonuclease E (EC 3.1.26.12)
   PGF_10525969 Magnesium and cobalt efflux protein CorC

8. 23S rRNA (uracil(1939)-C(5))-methyltransferase (EC 2.1.1.190). Methylation of 23S rRNA was found to provide a significant advantage for bacteria at osmotic and oxidative stress [39].

   Other PGFams in the corresponding cluster: None.

9. YpfJ protein, zinc metalloprotease superfamily, is a protein that cleaves other proteins and uses zinc as a metal cofactor. Metalloproteases play multiple roles in virulence including the disruption of physiologically important host processes, release of nutrients such as metals from host metalloproteins, cleavage of host immune components, and interference with host immune signaling cascades [40].

   Other PGFams in the corresponding cluster: None.

10. Threonine dehydratase biosynthetic (EC 4.3.1.19). Threonine dehydratase mediated isoleucine biosynthesis is an important step in maintaining the metabolic pool of isoleucine, a branch chain amino acid. It has been shown that down-regulation of threonine dehydratase in the pathogen *Mycobacterium tuberculosis* increases its susceptibility to oxidative stress [41]. It also has been suggested that the genes for threonine biosynthesis are critical factors for the multiplication of *Staphylococcus aureus* in the blood [42]. Although S. aureus is usually commensal in the skin and the mucosa, its presence in the blood can lead to a bloodstream infection with a high fatality rate [43]. The enzymes belonging to the branch chain amino acid biosynthetic pathway

in bacteria are promising drug targets due to the lack of a similar pathway in mammals, which would reduce related toxicity [44].

Other PGFams in the corresponding cluster: None.

11. The enzyme glutathione reductase (EC 1.8.1.7) is part of the antioxidant glutathione system. Glutathione is an abundant antioxidant in bacteria, where it serves a key function in protecting the cell from the action of low pH, chlorine compounds, osmotic stresses, and reactive oxygen species (ROS) [45]. Glutathione reductase is one of the main enzymes involved in glutathione metabolism [45]. Recent studies suggested that generation of ROS acts as a common mechanism of antibiotics-induced bacterial death, thus inhibiting antioxidant systems such as the glutathione system may limit antibiotic resistance [46].

    Other PGFams in the corresponding cluster: None.

12. Cell division integral membrane protein, YggT and half-length relatives. This is an unknown gene with the predicted function of a cell division integral membrane protein, and its gene symbol is YggT. YggT seems to play a role in osmotic stress tolerance in Escherichia coli [47]. It has been suggested that osmostress responsive systems contribute to the virulence potential of a number of pathogenic bacteria [48].

    Other PGFams in the corresponding cluster: None.

13. Superoxide dismutase [Cu-Zn] precursor (EC 1.15.1.1) enzyme. As part of the innate immune response, macrophages and neutrophils attack invading microbes with toxic superoxide [49]. To counteract this attack, some microbial pathogens express Cu, Zn superoxide dismutase enzymes [50]. This enzyme was shown to induce protection against oxidative stress and enhance the pathogenicity of *Bacillus anthracis* [51]. In addition, it was shown to contribute to the resistance of *Mycobacterium tuberculosis* against oxidative burst products generated by macrophages [52].

    Other PGFams in the corresponding cluster: None.

14. Sulfur carrier protein FdhD. FdhD is required for formate dehydrogenase to be functional. Formate dehydrogenase was suggested to be involved in oxidative stress tolerance in E. coli [53].

    Other PGFams in the corresponding cluster: None.

15. Deoxyribodipyrimidine photolyase. Photolyases are enzymes that repair UV-induced DNA lesions by using light energy. Interestingly, many human and plant pathogens contain photolyases [54].

    Other PGFams in the corresponding cluster: None.

S5.2 Top NHP Features

	PGFam	Function	MDI (SD[1])	HPs	NHPs	P-Ratio[2]	# Genera[3]
1	PGF_03029062	Dihydroorotate dehydrogenase (NAD(+)), catalytic subunit (EC 1.3.1.14)	0.034 (0.01)	70	182	5.17	98
2	PGF_01667671	Cytidylate kinase (EC 2.7.4.25)	0.031 (0.009)	7	125	31.57	60
3	PGF_02930287	Reverse rubrerythrin	0.027 (0.01)	11	125	21.05	57
4	PGF_08946513	Flavodoxin	0.021 (0.007)	67	170	5.04	94
5	PGF_01469197	RNA methyltransferase, TrmA family	0.021 (0.009)	76	180	4.71	100
6	PGF_01333294	Activator of (R)-2-hydroxyglutaryl-CoA dehydratase	0.019 (0.007)	25	128	9.95	77
7	PGF_01284176	Rubrerythrin	0.018 (0.009)	35	142	7.96	81
8	PGF_00006245	Formate–tetrahydrofolate ligase (EC 6.3.4.3)	0.016 (0.007)	138	201	2.91	122
9	PGF_00033940	Phosphoribosylaminoimidazolecarboxamide formyltransferase (EC 2.1.2.3)	0.016 (0.006)	6	106	30.64	53
10	PGF_10332317	Electron transport complex protein RnfB	0.014 (0.006)	18	123	13.08	62
11	PGF_08126536	Flavoprotein	0.013 (0.006)	13	114	16.47	58
12	PGF_00401757	no significant homology.	0.013 (0.006)	8	106	23.83	44
13	PGF_00016404	LSU ribosomal protein L32p @ LSU ribosomal protein L32p, zinc-dependent	0.012 (0.005)	88	173	3.92	100
14	PGF_00075770	FIG00519347: Ribonucleotide reductase-like protein	0.012 (0.003)	51	135	5.24	76
15	PGF_00003251	Macrolide export ATP-binding/permease protein	0.01 (0.002)	16	69	8.25	40

Table S1: The top NHP PGFams that serve as features of the WSPC classifier according to their average Mean Decrease Impurity (MDI) rank, along with the number of HP and NHP genomes in the training set that contain the respective PGFams. The average MDI rank of a PGFam is the average value of the feature's MDI values computed using 100 random forest classifiers with different random seeds trained on the training set. [1]Standard Deviation. [2]The ratio between the proportion of NHPs with the corresponding PGFam and the proportion of HPs with the corresponding PGFam. To avoid zero division, add-one smoothing was performed. [3] The number of different genera to which the genomes that contain the respective PGFams belong.

S6 BacPaCS Test Genomes

	Genome ID	Genome Name	BacPaCS Label[1]	Validated Label [2]	References[3]	OPP[4]	Balanced Test[5]
1	470.3353	Acinetobacter baumannii strain HWBA8	NHP	HP	Clinical isolate [55]	[56]	Yes
2	106654.48	Acinetobacter nosocomialis strain SSA3	NHP	HP	Clinical isolate [55]	[56]	Yes
3	520.659	Bordetella pertussis strain B227	HP	HP	[57]		Yes
4	28450.385	Burkholderia pseudomallei strain MSHR5864	HP	HP	[58]	[59]	Yes
5	83554.74	Chlamydia psittaci strain GIMC 2005:CpsCP1	HP	HP	[60]		Yes
6	813.141	Chlamydia trachomatis strain SQ12	HP	HP	[61]		Yes
7	545.38	Citrobacter koseri strain FDAARGOS_287	HP	HP	[62, 63]	[63]	Yes
8	777.186	Coxiella burnetii strain Heizberg	HP	HP	[64]		Yes
9	1352.1760	Enterococcus faecium strain Ef_aus00233	NHP	HP	Hospital outbreak isolate [65]	[66]	Yes
10	562.22306	Escherichia coli strain FDAARGOS_433 strain Not applicable	HP	HP	[67]		Yes
11	210.2912	Helicobacter pylori strain F20	HP	HP	[68]		Yes
12	573.16474	Klebsiella pneumoniae strain KP9	HP	HP	[69, 70]	[70]	Yes
13	1639.2624	Listeria monocytogenes strain H34	HP	HP	[71]	[71]	Yes
14	1041522.28	Mycobacterium colombiense CECT 3035 strain CECT 3035	HP	HP	[72]	[72]	Yes
15	722731.3	Mycobacterium shigaense strain UN-152	HP	HP	[73]	[74]	Yes
16	1773.8714	Mycobacterium tuberculosis strain MDRMA2441	HP	HP	[75]		Yes
17	2104.190	Mycoplasma pneumoniae strain KCH-405	HP	HP	[76]		Yes
18	37326.9	Nocardia brasiliensis strain FDAARGOS_352	HP	HP	[77, 78]		Yes
19	28131.10	Prevotella intermedia strain OMA14	HP	HP	[79]	[80]	Yes
20	287.4623	Pseudomonas aeruginosa strain PB350	HP	HP	[81]	[82]	Yes
21	1280.11681	Staphylococcus aureus strain USA300-SUR15	HP	HP	[83]	[84]	Yes
22	1302.83	Streptococcus gordonii strain FDAARGOS_257	HP	HP	[85, 86]	[86]	Yes
23	1338.30	Streptococcus intermedius strain FDAARGOS_233	HP	HP	[87]	[87]	Yes
24	1902136.3	Streptococcus sp. NPS 308	NHP	HP	Clinical isolate [88]	[88]	Yes
25	730.54	[Haemophilus] ducreyi strain FDAARGOS_297	HP	HP	[89, 90]		Yes
26	28025.19	Bifidobacterium animalis strain BL3	NHP	NHP	[91]		Yes
27	1496.1556	Clostridioides difficile strain BR81	NHP	NHP	[92, 93]	[93]	Yes
28	74426.49	Collinsella aerofaciens strain indica	NHP	NHP	[94]	[95]	Yes
29	853.172	Faecalibacterium prausnitzii strain Indica	NHP	NHP	[96]		Yes
30	1841863.3	Gordonibacter sp. Marseille-P2775 strain Marseille-P2775	NHP	NHP	[97]		Yes
31	1582.99	Lactobacillus casei strain LC5	NHP	NHP	[98]		Yes
32	1613.133	Lactobacillus fermentum strain FTDC 8312	NHP	NHP	[99]		Yes
33	1590.548	Lactobacillus plantarum strain LP3	NHP	NHP	[100]		Yes
34	47715.277	Lactobacillus rhamnosus strain Pen	NHP	NHP	[101]		Yes

Continued on next page

	Genome ID	Genome Name	BacPaCS Label[1]	Validated Label [2]	References[3]	OPP[4]	Balanced Test[5]
35	487.1231	Neisseria meningitidis strain 38277	NHP	NHP	[102]	[102]	Yes
36	1944646.3	Phoenicibacter massiliensis strain Marseille-P3241	NHP	NHP	[103]		Yes
37	1849491.6	Staphylococcaceae bacterium S31 strain S31	NHP	NHP	[104]		Yes
38	1308.247	Streptococcus thermophilus strain ST3	NHP	NHP	[105]		Yes
39	137591.34	Weissella cibaria strain CMS3	NHP	NHP	[106]		Yes
40	515619.32	[Eubacterium rectale] ATCC 33656	NHP	NHP	[107]		Yes
41	470.3349	Acinetobacter baumannii strain 15A34	NHP	HP	Clinical isolate [55]		No
42	470.3350	Acinetobacter baumannii strain 15A5	NHP	HP	Clinical isolate [55]		No
43	470.3348	Acinetobacter baumannii strain CBA7	NHP	HP	Clinical isolate [55]		No
44	470.3347	Acinetobacter baumannii strain JBA13	NHP	HP	Clinical isolate [55]		No
45	470.3356	Acinetobacter baumannii strain SAA14	NHP	HP	Clinical isolate [55]		No
46	470.3357	Acinetobacter baumannii strain SSA12	NHP	HP	Clinical isolate [55]		No
47	470.3352	Acinetobacter baumannii strain SSA6	NHP	HP	Clinical isolate [55]		No
48	470.3358	Acinetobacter baumannii strain SSMA17	NHP	HP	Clinical isolate [55]		No
49	470.3355	Acinetobacter baumannii strain USA15	NHP	HP	Clinical isolate [55]		No
50	470.3351	Acinetobacter baumannii strain USA2	NHP	HP	Clinical isolate [55]		No
51	470.3354	Acinetobacter baumannii strain WKA02	NHP	HP	Clinical isolate [55]		No
52	520.747	Bordetella pertussis strain C934	HP	HP	[57]		No
53	520.694	Bordetella pertussis strain H361	HP	HP	[57]		No
54	520.667	Bordetella pertussis strain H698	HP	HP	[57]		No
55	520.529	Bordetella pertussis strain H754	HP	HP	[57]		No
56	520.670	Bordetella pertussis strain H771	HP	HP	[57]		No
57	520.535	Bordetella pertussis strain H812	HP	HP	[57]		No
58	520.698	Bordetella pertussis strain H876	HP	HP	[57]		No
59	520.699	Bordetella pertussis strain I093	HP	HP	[57]		No
60	520.572	Bordetella pertussis strain I112	HP	HP	[57]		No
61	520.537	Bordetella pertussis strain I238	HP	HP	[57]		No
62	520.629	Bordetella pertussis strain I461	HP	HP	[57]		No
63	520.543	Bordetella pertussis strain I763	HP	HP	[57]		No
64	520.574	Bordetella pertussis strain I998	HP	HP	[57]		No
65	520.546	Bordetella pertussis strain J018	HP	HP	[57]		No
66	520.650	Bordetella pertussis strain J073	HP	HP	[57]		No
67	520.737	Bordetella pertussis strain J078	HP	HP	[57]		No
68	520.708	Bordetella pertussis strain J122	HP	HP	[57]		No
69	520.671	Bordetella pertussis strain J178	HP	HP	[57]		No
70	520.672	Bordetella pertussis strain J179	HP	HP	[57]		No
71	520.678	Bordetella pertussis strain J194	HP	HP	[57]		No
72	520.680	Bordetella pertussis strain J296	HP	HP	[57]		No
73	28450.655	Burkholderia pseudomallei strain 2010007509	HP	HP	[108, 59]	[59]	No
74	813.142	Chlamydia trachomatis strain SQ14	HP	HP	[61]		No
75	168807.6	Escherichia coli O127:H6 strain EPEC1	NHP	HP	Pathogenic *E. coli* strain [109]		No
76	562.16466	Escherichia coli strain 5CRE51	HP	HP	[110]		No
77	562.22333	Escherichia coli strain ATCC 43896	HP	HP	[111]		No

Continued on next page

	Genome ID	Genome Name	BacPaCS Label[1]	Validated Label [2]	References[3]	OPP[4]	Balanced Test[5]
78	562.15193	Escherichia coli strain Ecol_276	HP	HP	[112]		No
79	562.22323	Escherichia coli strain FDAARGOS_434 strain Not applicable	HP	HP	[113]		No
80	562.22307	Escherichia coli strain FDAARGOS_448 strain Not applicable	HP	HP	[114]		No
81	562.16428	Escherichia coli strain G199	NHP	HP	Pathogenic *E. coli* strain [115]		No
82	562.22326	Escherichia coli strain UFU_EC98	HP	HP	[116]		No
83	573.16440	Klebsiella pneumoniae strain 459	HP	HP	[117]	[70]	No
84	573.15319	Klebsiella pneumoniae strain FDAARGOS_436 strain Not applicable	HP	HP	[118]	[70]	No
85	1196172.3	Listeria monocytogenes serotype 4b str. 02-1289 strain 02-1289	HP	HP	[119, 71]	[71]	No
86	1196162.3	Listeria monocytogenes serotype 4b str. 10-0809 strain 10-0809	HP	HP	[120, 71]	[71]	No
87	1773.8686	Mycobacterium tuberculosis strain LE13	HP	HP	[75]		No
88	1773.8719	Mycobacterium tuberculosis strain TBDM2489	HP	HP	[75]		No
89	2104.189	Mycoplasma pneumoniae strain KCH-402	HP	HP	[76]		No
90	487.1548	Neisseria meningitidis strain M26417	HP	HP	[121]	[102]	No
91	287.3868	Pseudomonas aeruginosa strain RIVM-EMC2982	NHP	HP	Clinical isolate [122]	[82]	No
92	1280.12234	Staphylococcus aureus strain JE2	HP	HP	[123, 84]	[84]	No
93	1280.11677	Staphylococcus aureus strain USA300-SUR11	HP	HP	[124, 84]	[84]	No
94	76857.43	Fusobacterium nucleatum subsp. polymorphum strain KCOM 1275	NHP	INC	[12]		No
95	172042.4	Rothia aeria strain JCM 11412	NHP	INC		[14]	No
96	860.11	Fusobacterium periodonticum strain KCOM 1261	NHP	INC	[125]		No
97	860.17	Fusobacterium periodonticum strain KCOM 1262	NHP	INC	[125]		No
98	860.16	Fusobacterium periodonticum strain KCOM 2555	NHP	INC	[125]		No
99	860.18	Fusobacterium periodonticum strain KCOM 2653	NHP	INC	[125]		No
100	47715.310	Lactobacillus rhamnosus strain LR5	NHP	NHP	[126]		No

Table S2: A list of genomes included in the BacPaCS test set. [1]The original label given to each genome by Barash *et al.* [7]. [2,3]Manually validated label according to the PATRIC metadata entry and according to the literature, along with the respective reference. INC stands for an inconclusive label, see text. [4]A reference is provided if the corresponding species is known to cause opportunistic infections. [5]Whether the genome is part of the balanced version of the BacPaCS test, which includes one genomes per species.

S7 WSPC Test Genomes

	Genome ID	Genome Name	Label	HP/NHP[1]	Species	References[2]	OPP.[3]
1	163603.4	Actinomadura latina strain ATCC BAA-277	HP	1/0	Actinomadura latina	[127]	No
2	648.157	Aeromonas caviae strain ScAc2001	HP	9/0	Aeromonas caviae	[128]	No
3	565.15	Atlantibacter hermannii strain 3608	HP	1/0	Atlantibacter hermannii	[129]	No

Continued on next page

	Genome ID	Genome Name	Label	HP/NHP[1]	Species	References[2]	OPP.[3]
4	29459.655	Brucella melitensis strain HN20190002	HP	13/0	Brucella melitensis	[130]	No
5	87883.284	Burkholderia multivorans strain C1576	HP	107/0	Burkholderia multivorans	[131]	No
6	195.2778	Campylobacter coli strain 202823	HP	95/0	Campylobacter coli	[132, 133]	No
7	201.69	Campylobacter lari strain 503734	HP	3/0	Campylobacter lari	[134, 135]	No
8	2572066.4	Campylobacter sp. CFSAN093243	HP	194/0	Campylobacter jejuni	[134, 136, 137]	No
9	1491.1756	Clostridium botulinum strain ZBS3_16-240-01	HP	7/0	Clostridium botulinum	[138]	No
10	65058.433	Corynebacterium ulcerans strain 02-13	HP	8/0	Corynebacterium ulcerans	[139]	No
11	208962.153	Escherichia albertii strain 13S38	HP	34/0	Escherichia albertii	[140]	No
12	562.55247	Escherichia coli strain 4374	HP	4352/396	Escherichia coli	[141]	No
13	210.6620	Helicobacter pylori strain MHP47	HP	526/0	Helicobacter pylori	[142]	No
14	104628.50	Helicobacter suis strain NHP19-4022	HP	2/0	Helicobacter suis	[143]	No
15	44275.61	Leptospira interrogans serovar Copenhageni strain CLEP00179	HP	6/0	Leptospira interrogans	[144]	No
16	1639.7756	Listeria monocytogenes strain SCPM-O-B-7909	HP	197/0	Listeria monocytogenes	[145]	No
17	78331.108	Mycobacterium canettii strain NLA000701671	HP	1/0	Mycobacterium canettii	[146]	No
18	1768.199	Mycobacterium kansasii strain JALMAMYKAN-1	HP	34/0	Mycobacterium kansasii	[147]	No
19	2664891.3	Mycobacterium tuberculosis complex sp. AY1MRC	HP	1/0	Mycobacterium tuberculosis complex sp. AY1MRC	[148]	No
20	1773.20690	Mycobacterium tuberculosis strain Mycobacterium tuberculosis 79499	HP	3445/2	Mycobacterium tuberculosis	[149]	No
21	485.8128	Neisseria gonorrhoeae strain 5671 strain not applicable	HP	289/0	Neisseria gonorrhoeae	[150]	No
22	90370.3046	Salmonella enterica subsp. enterica serovar Typhi strain 343077_206161	HP	1480/2	Salmonella enterica	[151]	No
23	624.2242	Shigella sonnei strain 7109.28	HP	850/0	Shigella sonnei	[152]	No
24	686.65	Vibrio cholerae O1 biovar El Tor strain NALMLE37	HP	344/0	Vibrio cholerae	[153]	No
25	150053.31	Yersinia enterocolitica subsp. palearctica strain Ye9N	HP	5/0	Yersinia enterocolitica	[154]	No
26	85698.208	Achromobacter xylosoxidans strain DN2019	HP	16/0	Achromobacter xylosoxidans	[155]	Yes
27	470.9648	Acinetobacter baumannii strain KT_2016_39	HP	2281/0	Acinetobacter baumannii	[156]	Yes
28	29430.96	Acinetobacter haemolyticus strain 11654	HP	25/0	Acinetobacter haemolyticus	[157]	Yes
29	28090.110	Acinetobacter lwoffii strain FDAARGOS_620 strain Not applicable	HP	2/0	Acinetobacter lwoffii	[158]	Yes
30	48296.376	Acinetobacter pittii strain VNMU 150	HP	43/2	Acinetobacter pittii	[159]	Yes
31	2026190.21	Bacillus mobilis strain 1428.155	HP	1/0	Bacillus mobilis	[160, 161]	Yes
32	2026186.33	Bacillus paranthracis strain ELWA-3_3298	HP	1/1	Bacillus paranthracis	[162]	Yes
33	817.1653	Bacteroides fragilis strain PP971	HP	61/45	Bacteroides fragilis	[163]	Yes

Continued on next page

	Genome ID	Genome Name	Label	HP/NHP[1]	Species	References[2]	OPP.[3]
34	95486.460	Burkholderia cenocepacia strain MS-2140	HP	206/0	Burkholderia cenocepacia	[164]	Yes
35	292.237	Burkholderia cepacia strain IST612	HP	3/0	Burkholderia cepacia	[165]	Yes
36	28450.2018	Burkholderia pseudomallei strain haikou8	HP	168/0	Burkholderia pseudomallei	[59]	Yes
37	1507806.57	Campylobacter fetus subsp. testudinum strain wqj4	HP	14/0	Campylobacter fetus	[166]	Yes
38	57706.80	Citrobacter braakii strain CB00017	HP	1/0	Citrobacter braakii	[167]	Yes
39	67824.24	Citrobacter farmeri strain YDC697-2	HP	1/0	Citrobacter farmeri	[63, 168]	Yes
40	546.797	Citrobacter freundii strain YDC638-3	HP	34/1	Citrobacter freundii	[63]	Yes
41	545.121	Citrobacter koseri strain AS012499	HP	7/1	Citrobacter koseri	[63]	Yes
42	67827.42	Citrobacter werkmanii strain YDC667-1	HP	4/0	Citrobacter werkmanii	[63]	Yes
43	133448.15	Citrobacter youngae strain AS012330	HP	1/0	Citrobacter youngae	[63]	Yes
44	1496.5104	Clostridioides difficile strain TMD0138	HP	601/39	Clostridioides difficile	[93, 169]	Yes
45	413503.68	Cronobacter malonaticus strain BJ15	HP	8/0	Cronobacter malonaticus	[170]	Yes
46	413501.15	Cronobacter muytjensii strain Cr150	HP	1/0	Cronobacter muytjensii	[170]	Yes
47	28141.803	Cronobacter sakazakii strain SD45	HP	47/0	Cronobacter sakazakii	[170]	Yes
48	413502.35	Cronobacter turicensis strain SH11	HP	2/0	Cronobacter turicensis	[170]	Yes
49	539.170	Eikenella corrodens strain EI_09	HP	8/0	Eikenella corrodens	[171, 172]	Yes
50	2528037.4	Eikenella exigua strain EI_02	HP	1/0	Eikenella exigua	[171, 173]	Yes
51	1117645.412	Elizabethkingia anophelis strain PHOL-515	HP	11/0	Elizabethkingia anophelis	[174]	Yes
52	238.101	Elizabethkingia meningoseptica strain GX196	HP	6/0	Elizabethkingia meningoseptica	[174]	Yes
53	61645.317	Enterobacter asburiae strain TUM17941	HP	20/0	Enterobacter asburiae	[175]	Yes
54	2494701.5	Enterobacter chengduensis strain C2-143-1	HP	2/0	Enterobacter chengduensis	[176]	Yes
55	550.2644	Enterobacter cloacae strain AS012445	HP	119/1	Enterobacter cloacae	[176]	Yes
56	299767.100	Enterobacter ludwigii strain AS012471	HP	6/0	Enterobacter ludwigii	[175]	Yes
57	1812935.59	Enterobacter roggenkampii strain AS012293	HP	12/0	Enterobacter roggenkampii	[177]	Yes
58	1351.3480	Enterococcus faecalis strain VNMU281	HP	98/13	Enterococcus faecalis	[178, 179]	Yes
59	1353.134	Enterococcus gallinarum strain EGR748	HP	2/1	Enterococcus gallinarum	[180, 181]	Yes
60	727.2505	Haemophilus influenzae strain AS012767	HP	409/0	Haemophilus influenzae	[182]	Yes
61	729.1932	Haemophilus parainfluenzae strain COPD-014-E1 O	HP	3/0	Haemophilus parainfluenzae	[183]	Yes
62	548.678	Klebsiella aerogenes strain AS012329	HP	96/0	Klebsiella aerogenes	[184]	Yes
63	1134687.181	Klebsiella michiganensis strain AS012446	HP	13/0	Klebsiella michiganensis	[185]	Yes
64	571.798	Klebsiella oxytoca strain AS012479	HP	28/0	Klebsiella oxytoca	[186]	Yes
65	573.29103	Klebsiella pneumoniae strain ST974-OXA48	HP	3859/8	Klebsiella pneumoniae	[186]	Yes

Continued on next page

	Genome ID	Genome Name	Label	HP/NHP[1]	Species	References[2]	OPP.[3]
66	1463165.235	Klebsiella quasipneumoniae strain M36	HP	67/0	Klebsiella quasipneumoniae	[187]	Yes
67	244366.462	Klebsiella variicola strain AS012291	HP	129/0	Klebsiella variicola	[187]	Yes
68	109328.8	Leptotrichia trevisanii strain JMUB3870	HP	1/0	Leptotrichia trevisanii	[188]	Yes
69	480.450	Moraxella catarrhalis strain AS012766	HP	64/0	Moraxella catarrhalis	[189]	Yes
70	582.399	Morganella morganii strain AS012332	HP	18/0	Morganella morganii	[190]	Yes
71	1809.19	Mycobacterium ulcerans strain CSURQ0185	HP	3/0	Mycobacterium ulcerans	[191]	Yes
72	36809.633	Mycobacteroides abscessus strain 1322-S0	HP	682/0	Mycobacteroides abscessus	[192]	Yes
73	948102.18	Mycobacteroides franklinii strain 9917	HP	1/0	Mycobacteroides franklinii	[193]	Yes
74	487.3238	Neisseria meningitidis strain N186_00	HP	278/43	Neisseria meningitidis	[102]	Yes
75	455432.12	Nocardia terpenica strain IFM 0706	HP	1/0	Nocardia terpenica	[194, 195]	Yes
76	419475.21	Ochrobactrum pseudogrignonense strain SHIN	HP	1/0	Ochrobactrum pseudogrignonense	[196, 197]	Yes
77	1202713.3	Paenalcaligenes suwonensis strain 191B	HP	1/0	Paenalcaligenes suwonensis	[198]	Yes
78	44250.11	Paenibacillus alvei strain bk032014	HP	1/0	Paenibacillus alvei	[199]	Yes
79	753.7	Pasteurella canis strain QBSD	HP	1/0	Pasteurella canis	[200]	Yes
80	584.1255	Proteus mirabilis strain L76	HP	68/2	Proteus mirabilis	[201]	Yes
81	585.92	Proteus vulgaris strain AS012427	HP	2/0	Proteus vulgaris	[202]	Yes
82	588.149	Providencia stuartii strain AS012498	HP	7/0	Providencia stuartii	[203]	Yes
83	287.11178	Pseudomonas aeruginosa strain PASP309	HP	1957/0	Pseudomonas aeruginosa	[204]	Yes
84	46680.55	Pseudomonas nitroreducens strain SC-1148-IPA-05	HP	1/0	Pseudomonas nitroreducens	[204, 205]	Yes
85	2681497.3	Pseudomonas sp. AU8050	HP	32/0	unclassified Pseudomonas	[206, 207]	Yes
86	190721.31	Ralstonia insidiosa strain 5047 strain not applicable	HP	1/0	Ralstonia insidiosa	[208]	Yes
87	54291.261	Raoultella ornithinolytica strain Z&Z370	HP	10/0	Raoultella ornithinolytica	[209]	Yes
88	575.68	Raoultella planticola strain AS012264	HP	5/0	Raoultella planticola	[210]	Yes
89	648995.31	Rhizobium pusense strain FDAARGOS_618 strain Not applicable	HP	3/0	Rhizobium pusense	[211]	Yes
90	615.1093	Serratia marcescens strain C3	HP	137/0	Serratia marcescens	[212]	Yes
91	29378.48	Staphylococcus arlettae strain N283	HP	2/0	Staphylococcus arlettae	[213]	Yes
92	1280.23630	Staphylococcus aureus strain SA15KEN strain not applicable	HP	2605/61	Staphylococcus aureus	[214, 215]	Yes
93	29388.243	Staphylococcus capitis strain 12-400	HP	29/1	Staphylococcus capitis	[216]	Yes
94	1283.793	Staphylococcus haemolyticus strain 166 strain not applicable	HP	131/0	Staphylococcus haemolyticus	[217]	Yes
95	2045451.27	Stenotrophomonas indicatrix strain AS012656	HP	1/0	Stenotrophomonas indicatrix	[218, 219]	Yes
96	40324.1544	Stenotrophomonas maltophilia strain AS012600	HP	216/1	Stenotrophomonas maltophilia	[220]	Yes
97	119602.327	Streptococcus dysgalactiae subsp. equisimilis strain KNZ15	HP	15/0	Streptococcus dysgalactiae	[221]	Yes
98	254785.37	Streptococcus halichoeri strain CCUG67100	HP	1/0	Streptococcus halichoeri	[222]	Yes

Continued on next page

	Genome ID	Genome Name	Label	HP/NHP[1]	Species	References[2]	OPP.[3]
99	1313.20171	Streptococcus pneumoniae strain GPSC334	HP	4748/235	Streptococcus pneumoniae	[223, 224]	Yes
100	257758.659	Streptococcus pseudopneumoniae strain CCUG 72018	HP	40/0	Streptococcus pseudopneumoniae	[225]	Yes
101	1343.51	Streptococcus vestibularis strain AS012761	HP	1/1	Streptococcus vestibularis	[226, 227]	Yes
102	40545.1254	Sutterella wadsworthensis strain 809h	HP	20/0	Sutterella wadsworthensis	[228, 229]	Yes
103	1648923.136	Bacillus paralicheniformis strain 6-1	NHP	0/2	Bacillus paralicheniformis	[230, 231]	No
104	1423.1028	Bacillus subtilis strain 8-1	NHP	0/3	Bacillus subtilis	[232, 233]	No
105	492670.508	Bacillus velezensis strain Marseille-Q1230	NHP	0/1	Bacillus velezensis	[234, 235]	No
106	28116.1355	Bacteroides ovatus strain F11	NHP	2/28	Bacteroides ovatus	[236, 237]	No
107	820.4961	Bacteroides uniformis strain A23	NHP	2/46	Bacteroides uniformis	[238, 239]	No
108	371601.509	Bacteroides xylanisolvens strain BIOML-A67	NHP	0/11	Bacteroides xylanisolvens	[240, 241]	No
109	1680.1978	Bifidobacterium adolescentis strain BIO5485	NHP	0/24	Bifidobacterium adolescentis	[242]	No
110	28025.139	Bifidobacterium animalis strain BIOML-A2	NHP	0/6	Bifidobacterium animalis	[240, 243]	No
111	1681.836	Bifidobacterium bifidum strain BIOML-A20	NHP	0/33	Bifidobacterium bifidum	[240, 244]	No
112	1689.110	Bifidobacterium dentium strain UT_Austin_Bifido_FMT_C1	NHP	0/4	Bifidobacterium dentium	[245]	No
113	1679.240	Bifidobacterium longum subsp. longum strain BIO6283	NHP	0/60	Bifidobacterium longum	[246]	No
114	1720313.7	Bittarella massiliensis strain BIOML-A2	NHP	0/2	Bittarella massiliensis	[240, 247]	No
115	1737424.51	Blautia massiliensis strain BIOML-A2	NHP	0/4	Blautia massiliensis	[240, 248]	No
116	40520.1620	Blautia obeum strain BIOML-A1	NHP	0/28	Blautia obeum	[240, 249]	No
117	2584624.3	Blautia sp. BIOML-A1	NHP	0/40	unclassified Blautia	[240, 250]	No
118	418240.274	Blautia wexlerae strain BIOML-A15	NHP	0/17	Blautia wexlerae	[240, 251]	No
119	2584625.3	Butyricicoccus sp. BIOML-A1	NHP	0/18	unclassified Butyricicoccus	[240, 252]	No
120	2584626.3	Catenibacterium sp. BIOML-A1	NHP	0/4	unclassified Catenibacterium	[240, 253]	No
121	1520.337	Clostridium beijerinckii strain BIOML-A8	NHP	0/11	Clostridium beijerinckii	[240, 254]	No
122	2547410.3	Clostridium sp. C5-48	NHP	1/88	unclassified Clostridium	[10]	No
123	2584634.3	Coprobacillus sp. BIOML-A1	NHP	0/39	unclassified Coprobacillus	[240, 255]	No
124	410072.542	Coprococcus comes strain F22	NHP	0/6	Coprococcus comes	[256]	No
125	2584635.3	Coprococcus sp. BIOML-A1	NHP	0/17	unclassified Coprococcus	[240, 257]	No
126	88431.891	Dorea longicatena strain BIOML-A2	NHP	0/14	Dorea longicatena	[240, 258]	No
127	2584637.3	Dorea sp. BIOML-A1	NHP	0/9	unclassified Dorea	[240, 259]	No
128	39490.144	Eubacterium ramulus strain BIOML-A2	NHP	0/4	Eubacterium ramulus	[240, 260]	No

Continued on next page

	Genome ID	Genome Name	Label	HP/NHP[1]	Species	References[2]	OPP.[3]
129	2584644.3	Eubacterium sp. BIOML-A2	NHP	0/29	unclassified Eubacterium	[240, 261]	No
130	853.7356	Faecalibacterium prausnitzii strain BIOML-B10	NHP	0/39	Faecalibacterium prausnitzii	[240, 262]	No
131	2584645.3	Faecalibacterium sp. BIOML-A1	NHP	0/11	unclassified Faecalibacterium	[240, 263]	No
132	292800.500	Flavonifractor plautii strain BIOML-A3	NHP	0/3	Flavonifractor plautii	[240, 264]	No
133	1335613.28	Gordonibacter urolithinfaciens strain BIOML-A1	NHP	0/1	Gordonibacter urolithinfaciens	[240, 265]	No
134	2686092.3	Halobacillus sp. Marseille-Q1234	NHP	0/1	unclassified Halobacillus	[266]	No
135	1468449.9	Holdemania massiliensis strain BIOML-A4	NHP	0/5	Holdemania massiliensis	[240, 267]	No
136	1613.506	Lactobacillus fermentum strain CVM-347	NHP	1/11	Lactobacillus fermentum	[268]	No
137	1587.365	Lactobacillus helveticus strain DLBSA201	NHP	0/3	Lactobacillus helveticus	[269]	No
138	1597.462	Lactobacillus paracasei strain BIOML-A2	NHP	0/19	Lactobacillus paracasei	[240, 270]	No
139	47715.653	Lactobacillus rhamnosus strain BIOML-A3	NHP	2/20	Lactobacillus rhamnosus	[240, 271]	No
140	1623.232	Lactobacillus ruminis strain BIOML-A15	NHP	0/31	Lactobacillus ruminis	[240, 272]	No
141	2584658.3	Lactonifactor sp. BIOML-A5	NHP	0/7	unclassified Lactonifactor	[240, 273]	No
142	2584661.3	Megasphaera sp. BIOML-A1	NHP	0/4	unclassified Megasphaera	[240, 274]	No
143	28118.1621	Odoribacter splanchnicus strain BIOML-A4	NHP	0/16	Odoribacter splanchnicus	[240, 275]	No
144	823.3208	Parabacteroides distasonis strain BIOML-A11	NHP	5/66	Parabacteroides distasonis	[240, 276]	No
145	328812.119	Parabacteroides goldsteinii strain BIOML-A2	NHP	1/11	Parabacteroides goldsteinii	[240, 277]	No
146	46503.1849	Parabacteroides merdae strain BIOML-A28	NHP	0/43	Parabacteroides merdae	[240, 278]	No
147	487175.63	Parasutterella excrementihominis strain BIOML-A4	NHP	0/15	Parasutterella excrementihominis	[240, 279]	No
148	33025.448	Phascolarctobacterium faecium strain BIOML-A6	NHP	0/17	Phascolarctobacterium faecium	[240, 280]	No
149	2049039.16	Phascolarctobacterium sp. strain P2A-2	NHP	0/2	unclassified Phascolarctobacterium	[280]	No
150	571933.10	Piscibacillus halophilus strain Marseille-Q1613	NHP	0/1	Piscibacillus halophilus	[281]	No
151	2584670.3	Pseudoflavonifractor sp. BIOML-A8	NHP	0/17	unclassified Pseudoflavonifractor	[240, 282]	No
152	301302.2226	Roseburia faecis strain BIOML-A1	NHP	0/2	Roseburia faecis	[240, 283]	No
153	166486.885	Roseburia intestinalis strain BIOML-A1	NHP	0/6	Roseburia intestinalis	[240, 284]	No
154	40518.2573	Ruminococcus bromii strain BIOML-A2	NHP	3/12	Ruminococcus bromii	[240, 285]	No
155	592978.29	Ruminococcus faecis strain BIOML-A1	NHP	0/1	[Ruminococcus] faecis	[240, 286]	No
156	1550024.413	Ruthenibacterium lactatiformans strain BIOML-A15	NHP	0/13	Ruthenibacterium lactatiformans	[240, 287]	No
157	2681551.3	Staphylococcus sp. 170179	NHP	3/2	unclassified Staphylococcus	[288]	No

Continued on next page

	Genome ID	Genome Name	Label	HP/NHP[1]	Species	References[2]	OPP.[3]
158	1304.1361	Streptococcus salivarius strain BIOML-A17	NHP	2/43	Streptococcus salivarius	[240, 289]	No
159	2053618.40	Subdoligranulum sp. strain P1-4	NHP	0/6	unclassified Subdoligranulum	[290]	No
160	2093855.3	Veillonellaceae bacterium M2-8	NHP	0/3	unclassified Veillonellaceae	[291, 292]	No
161	39485.1649	[Eubacterium] eligens strain BIOML-A1	NHP	0/13	Lachnospira eligens	[240, 293]	No
162	39491.2484	[Eubacterium] rectale strain BIOML-A5	NHP	6/70	unclassified Lachnospiraceae	[240, 294]	No
163	33039.1028	[Ruminococcus] torques strain BIOML-A5	NHP	0/9	[Ruminococcus] torques	[240, 295]	No
164	1872444.542	Alistipes sp. strain P1-1	NHP	0/5	unclassified Alistipes	[296]	Yes
165	1396.2563	Bacillus cereus strain 2-1	NHP	16/7	Bacillus cereus	[297, 298]	Yes
166	79880.75	Bacillus clausii strain B619/R	NHP	5/0	Bacillus clausii	[299]	Yes
167	818.1282	Bacteroides thetaiotaomicron strain F9-2	NHP	2/15	Bacteroides thetaiotaomicron	[300, 301]	Yes
168	821.3918	Bacteroides vulgatus strain H23	NHP	3/70	Bacteroides vulgatus	[302]	Yes
169	2044936.47	Bacteroidia bacterium strain T-B-M_MAG_00007	NHP	0/3	unclassified Bacteroidia	[303]	Yes
170	2044595.31	Candidatus Gracilibacteria bacterium strain P-C-F_MAG_00005	NHP	0/2	unclassified Candidatus Gracilibacteria	[304]	Yes
171	2026720.132	Candidatus Saccharibacteria bacterium strain T-D-F_MAG_00008	NHP	0/20	unclassified Saccharibacteria	[303]	Yes
172	44737.7	Capnocytophaga sp. strain P-B-M_MAG_00008	NHP	0/1	unclassified Capnocytophaga	[305]	Yes
173	35703.89	Citrobacter amalonaticus strain BIOML-A5	NHP	1/13	Citrobacter amalonaticus	[240, 306]	Yes
174	1898207.3769	Clostridiales bacterium strain T-D-F_MAG_00006	NHP	0/9	unclassified Clostridiales	[303]	Yes
175	1492.181	Clostridium butyricum strain BIOML-A2	NHP	0/2	Clostridium butyricum	[240, 307]	Yes
176	74426.1601	Collinsella aerofaciens strain BIOML-A15	NHP	0/20	Collinsella aerofaciens	[240, 95]	Yes
177	2584631.3	Collinsella sp. BIOML-A4	NHP	0/82	unclassified Collinsella	[240, 95]	Yes
178	218538.1051	Dialister invisus strain P2A-1	NHP	0/1	Dialister invisus	[308]	Yes
179	2584641.3	Eggerthella sp. BIOML-A4	NHP	0/5	unclassified Eggerthella	[240, 309]	Yes
180	158836.472	Enterobacter hormaechei strain BIOML-A4	NHP	305/6	Enterobacter hormaechei	[240, 310]	Yes
181	33945.49	Enterococcus avium strain BIOML-A3	NHP	4/5	Enterococcus avium	[240, 311]	Yes
182	53345.154	Enterococcus durans strain BIOML-A46	NHP	0/67	Enterococcus durans	[240, 312]	Yes
183	1352.8997	Enterococcus faecium strain C59	NHP	666/7	Enterococcus faecium	[66]	Yes
184	1354.275	Enterococcus hirae strain BIOML-A52	NHP	0/87	Enterococcus hirae	[240, 313]	Yes
185	53346.66	Enterococcus mundtii strain BIOML-A5	NHP	1/14	Enterococcus mundtii	[240, 314]	Yes
186	71452.14	Enterococcus raffinosus strain BIOML-A1	NHP	0/1	Enterococcus raffinosus	[240, 315]	Yes
187	1260.144	Finegoldia magna strain BIOML-A2	NHP	10/2	Finegoldia magna	[240, 316]	Yes
188	2584653.3	Finegoldia sp. BIOML-A5	NHP	0/5	unclassified Finegoldia	[240, 317]	Yes
189	1871037.250	Flavobacteriaceae bacterium strain P-A-M_MAG_00002	NHP	1/3	unclassified Flavobacteriaceae	[318]	Yes

Continued on next page

	Genome ID	Genome Name	Label	HP/NHP[1]	Species	References[2]	OPP.[3]
190	471189.25	Gordonibacter pamelaeae strain BIOML-A2	NHP	0/1	Gordonibacter pamelaeae	[240, 319]	Yes
191	104608.5	Leptotrichia sp. strain T-B-M_MAG_00008	NHP	0/1	unclassified Leptotrichia	[303]	Yes
192	1246.49	Leuconostoc lactis strain BIOML-A1	NHP	0/2	Leuconostoc lactis	[240, 320]	Yes
193	2049035.5	Mogibacterium sp. strain T-C-M_MAG_00002	NHP	0/3	unclassified Mogibacterium	[303]	Yes
194	1505.72	Paeniclostridium sordellii strain BIOML-A6	NHP	4/2	Paeniclostridium sordellii	[240, 321]	Yes
195	165179.2440	Prevotella copri strain P2B-2	NHP	0/21	Prevotella copri	[322]	Yes
196	59823.699	Prevotella sp. strain P-A-F_MAG_00002	NHP	1/7	unclassified Prevotella	[323, 324, 325]	Yes
197	1282.3805	Staphylococcus epidermidis strain JH	NHP	85/69	Staphylococcus epidermidis	[326, 327]	Yes
198	1311.2752	Streptococcus agalactiae strain M134	NHP	236/5	Streptococcus agalactiae	[328]	Yes
199	1318.781	Streptococcus parasanguinis strain BIOML-A16	NHP	3/25	Streptococcus parasanguinis	[240, 329]	Yes
200	2584682.3	Streptococcus sp. BIOML-A1	NHP	4/5	unclassified Streptococcus	[240, 330]	Yes
201	39778.876	Veillonella dispar strain BIOML-A2	NHP	0/3	Veillonella dispar	[240, 331]	Yes
202	29466.1006	Veillonella parvula strain BIOML-A1	NHP	1/4	Veillonella parvula	[240, 332]	Yes
203	1522.125	[Clostridium] innocuum strain BIOML-A2	NHP	0/15	[Clostridium] innocuum	[240, 333]	Yes
204	2044938.12	candidate division SR1 bacterium strain P-B-M_MAG_00018	NHP	0/19	unclassified Bacteria	[303]	Yes

Table S3: A list of genomes included in the WSPC test set. [1]HP to NHP ratio among the labeled genomes of the corresponding species (before choosing a representative from each species). [2]References to literature or a database entry asserting the label and the "group" annotations the corresponding genome belongs to. [3]OPP: opportunistic.

References

[1] Ruth R Miller, Vincent Montoya, Jennifer L Gardy, David M Patrick, and Patrick Tang. Metagenomics for pathogen detection in public health. *Genome Medicine*, 5(9):81, 2013.

[2] Carlus Deneke, Robert Rentzsch, and Bernhard Y Renard. Paprbag: A machine learning approach for the detection of novel pathogens from ngs data. *Scientific Reports*, 7(1):1–13, 2017.

[3] Jakub M Bartoszewicz, Anja Seidel, Robert Rentzsch, and Bernhard Y Renard. Deepac: predicting pathogenic potential of novel dna with reverse-complement neural networks. *Bioinformatics*, 36(1):81–89, 2020.

[4] Massimo Andreatta, Morten Nielsen, Frank Møller Aarestrup, and Ole Lund. In silico prediction of human pathogenicity in the γ-proteobacteria. *PloS One*, 5(10):e13680, 2010.

[5] Gregorio Iraola, Gustavo Vazquez, Lucía Spangenberg, and Hugo Naya. Reduced set of virulence genes allows high accuracy prediction of bacterial pathogenicity in humans. *PloS One*, 7(8):e42144, 2012.

[6] Salvatore Cosentino, Mette Voldby Larsen, Frank Møller Aarestrup, and Ole Lund. Pathogenfinder-distinguishing friend from foe using bacterial whole genome sequence data. *PloS One*, 8(10):e77302, 2013.

[7] Eran Barash, Neta Sal-Man, Sivan Sabato, and Michal Ziv-Ukelson. Bacpacs—bacterial pathogenicity classification via sparse-svm. *Bioinformatics*, 35(12):2001–2008, 2019.

[8] Ulrike Kappler, Karen Davenport, Scott Beatson, Susan Lucas, Alla Lapidus, Alex Copeland, Kerrie W Berry, Tijana Glavina Del Rio, Nancy Hammon, Eileen Dalin, et al. Complete genome sequence of the facultatively chemolithoautotrophic and methylotrophic alpha proteobacterium starkeya novella type strain (atcc 8093 t). *Standards in Genomic Sciences*, 7(1):44–58, 2012.

[9] Maria C Urdaci, Philippe Bressollier, and Irina Pinchuk. Bacillus clausii probiotic strains: antimicrobial and immunomodulatory activities. *Journal of Clinical Gastroenterology*, 38:S86–S90, 2004.

[10] NCBI. Clostridium sp. c5-48, Genome ID 2547410.3. `https://www.ncbi.nlm.nih.gov/biosample?term=txid2547410`.

[11] Laurie Ann Ximénez-Fyvie, Anne D Haffajee, and Sigmund S Socransky. Microbial composition of supra-and subgingival plaque in subjects with adult periodontitis. *Journal of Clinical Periodontology*, 27(10):722–732, 2000.

[12] Yiping W Han. Fusobacterium nucleatum: a commensal-turned pathogen. *Current Opinion in Microbiology*, 23:141–147, 2015.

[13] Hwa-Sook Kim, Dae-Sil Lee, Young-Hyo Chang, Min Jung Kim, Sukhoon Koh, Joongsu Kim, Jin-Hyo Seong, Soo Keun Song, Hwan Seon Shin, Jae-Beum Son, et al. Application of rpob and zinc protease gene for use in molecular discrimination of fusobacterium nucleatum subspecies. *Journal of Clinical Microbiology*, 48(2):545–553, 2010.

[14] Takayuki Nambu, Osamu Tsuzukibashi, Satoshi Uchibori, Kazuyoshi Yamane, Takeshi Yamanaka, Hugo Maruyama, Pao-Li Wang, Naho Mugita, Hiroki Morioka, Kazuya Takahashi, et al. Complete genome sequence of rothia aeria type strain jcm 11412, isolated from air in the russian space laboratory mir. *Genome announcements*, 4(6):e01444–16, 2016.

[15] T Ryan Hoens and Nitesh V Chawla. Imbalanced datasets: from sampling to classifiers. *Imbalanced learning: Foundations, Algorithms, and Applications*, pages 43–59, 2013.

[16] Kendrick Boyd, Kevin H Eng, and C David Page. Area under the precision-recall curve: point estimates and confidence intervals. In *Joint European conference on machine learning and knowledge discovery in databases*, pages 451–466. Springer, 2013.

[17] James A Hanley and Barbara J McNeil. The meaning and use of the area under a receiver operating characteristic (roc) curve. *Radiology*, 143(1):29–36, 1982.

[18] Jin Huang and Charles X Ling. Using auc and accuracy in evaluating learning algorithms. *IEEE Transactions on Knowledge and Data Engineering*, 17(3):299–310, 2005.

[19] Jesse Davis and Mark Goadrich. The relationship between precision-recall and roc curves. In *Proceedings of the 23rd international conference on Machine learning*, pages 233–240, 2006.

[20] Leo Breiman. Manual on setting up, using, and understanding random forests v3. 1. *Statistics Department University of California Berkeley, CA, USA*, 1:58, 2002.

[21] Anne-Laure Boulesteix, Silke Janitza, Jochen Kruppa, and Inke R König. Overview of random forest methodology and practical guidance with emphasis on computational biology and bioinformatics. *Wiley Interdisciplinary Reviews: Data Mining and Knowledge Discovery*, 2(6):493–507, 2012.

[22] Gaël Varoquaux, Lars Buitinck, Gilles Louppe, Olivier Grisel, Fabian Pedregosa, and Andreas Mueller. Scikit-learn: Machine learning without learning the machinery. *GetMobile: Mobile Computing and Communications*, 19(1):29–33, 2015.

[23] Jun Fan, Qun Liu, Quan Hao, Maikun Teng, and Liwen Niu. Crystal structure of uroporphyrinogen decarboxylase from bacillus subtilis. *Journal of Bacteriology*, 189(9):3573–3580, 2007.

[24] Jacob E Choby and Eric P Skaar. Heme synthesis and acquisition in bacterial pathogens. *Journal of Molecular Biology*, 428(17):3408–3428, 2016.

[25] Brian J Sheehan, Janine T Bossé, Amanda J Beddek, Andrew N Rycroft, J Simon Kroll, and Paul R Langford. Identification of actinobacillus pleuropneumoniae genes important for survival during infection in its natural host. *Infection and immunity*, 71(7):3960–3970, 2003.

[26] Aart de Kok, Annechien F Hengeveld, Alejandro Martin, and Adrie H Westphal. The pyruvate dehydrogenase multi-enzyme complex from gram-negative bacteria. *Biochimica et Biophysica Acta (BBA)-Protein Structure and Molecular Enzymology*, 1385(2):353–366, 1998.

[27] Shuangping Shi and Sabine Ehrt. Dihydrolipoamide acyltransferase is critical for mycobacterium tuberculosis pathogenesis. *Infection and Immunity*, 74(1):56–63, 2006.

[28] D Scott Merrell, David L Hava, and Andrew Camilli. Identification of novel factors involved in colonization and acid tolerance of vibrio cholerae. *Molecular microbiology*, 43(6):1471–1491, 2002.

[29] Thierry Gonzales and Janine Robert-Baudouy. Bacterial aminopeptidases: properties and functions. *FEMS Microbiology Reviews*, 18(4):319–344, 1996.

[30] Guo Jing, SonG Xue, Li-fang ZOU, Hua-song ZOU, and Gong-you CHEN. The small and large subunits of carbamoyl-phosphate synthase exhibit diverse contributions to pathogenicity in xanthomonas citri subsp. citri. *Journal of Integrative Agriculture*, 14(7):1338–1347, 2015.

[31] Jaideep Behari, Lisa Stagon, and Stephen B Calderwood. pepa, a gene mediating ph regulation of virulence genes in vibrio cholerae. *Journal of Bacteriology*, 183(1):178–188, 2001.

[32] Hiro Nakamura, Keitarou Saiki, Tatsushi Mogi, and Yasuhiro Anraku. Assignment and functional roles of the cyoabcde gene products required for the escherichia coli bo-type quinol oxidase. *The Journal of Biochemistry*, 122(2):415–421, 1997.

[33] Tao Xu, Jian Han, Jia Zhang, Jiazhen Chen, Nan Wu, Wenhong Zhang, and Ying Zhang. Absence of protoheme ix farnesyltransferase ctab causes virulence attenuation but enhances pigment production and persister survival in mrsa. *Frontiers in Microbiology*, 7:1625, 2016.

[34] Emily Stevens, Maisem Laabei, Stewart Gardner, Greg A Somerville, and Ruth C Massey. Cytolytic toxin production by staphylococcus aureus is dependent upon the activity of the protoheme ix farnesyltransferase. *Scientific Reports*, 7(1):1–9, 2017.

[35] Yan Zhang and Vadim N Gladyshev. Molybdoproteomes and evolution of molybdenum utilization. *Journal of Molecular Biology*, 379(4):881–899, 2008.

[36] Rabeb Dhouib, Dk SM Pg Othman, Ama-Tawiah Essilfie, Phil M Hansbro, Jeffrey O Hanson, Alastair G McEwan, and Ulrike Kappler. Maturation of molybdoenzymes and its influence on the pathogenesis of non-typeable haemophilus influenzae. *Frontiers in Microbiology*, 6:1219, 2015.

[37] Qifeng Zhong, Bostjan Kobe, and Ulrike Kappler. Molybdenum enzymes and how they support virulence in pathogenic bacteria. *Frontiers in Microbiology*, 11:3185, 2020.

[38] Jeffrey C Waller, Kenneth W Ellens, Ghulam Hasnain, Sophie Alvarez, James R Rocca, and Andrew D Hanson. Evidence that the folate-dependent proteins ygfz and mnmeg have opposing effects on growth and on activity of the iron-sulfur enzyme miab. *Journal of Bacteriology*, 194(2):362–367, 2012.

[39] Ilya A Osterman, Petr V Sergiev, Philipp O Tsvetkov, Alexander A Makarov, Alexey A Bogdanov, and Olga A Dontsova. Methylated 23s rrna nucleotide m2g1835 of escherichia coli ribosome facilitates subunit association. *Biochimie*, 93(4):725–729, 2011.

[40] Li Ma, Austen Terwilliger, and Anthony W Maresso. Iron and zinc exploitation during bacterial pathogenesis. *Metallomics*, 7(12):1541–1554, 2015.

[41] Rishabh Sharma, Deepa Keshari, Kumar Sachin Singh, Shailendra Yadav, and Sudheer Kumar Singh. Mra_1571 is required for isoleucine biosynthesis and improves mycobacterium tuberculosis h37ra survival under stress. *Scientific Reports*, 6(1):1–12, 2016.

[42] Yuichi Oogai, Masaya Yamaguchi, Miki Kawada-Matsuo, Tomoko Sumitomo, Shigetada Kawabata, and Hitoshi Komatsuzawa. Lysine and threonine biosynthesis from aspartate contributes to staphylococcus aureus growth in calf serum. *Applied and Environmental Microbiology*, 82(20):6150–6157, 2016.

[43] Rasmus V Rasmussen, Vance G Fowler Jr, Robert Skov, and Niels E Bruun. Future challenges and treatment of staphylococcus aureus bacteremia with emphasis on mrsa. *Future Microbiology*, 6(1):43–56, 2011.

[44] Tathyana M Amorim Franco and John S Blanchard. Bacterial branched-chain amino acid biosynthesis: structures, mechanisms, and drugability. *Biochemistry*, 56(44):5849–5865, 2017.

[45] Lluis Masip, Karthik Veeravalli, and George Georgiou. The many faces of glutathione in bacteria. *Antioxidants & Redox Signaling*, 8(5-6):753–762, 2006.

[46] Xiaoyuan Ren, Lili Zou, and Arne Holmgren. Targeting bacterial antioxidant systems for antibiotics development. *Current Medicinal Chemistry*, 27(12):1922–1939, 2020.

[47] Tomokazu Ito, Nobuyuki Uozumi, Tatsunosuke Nakamura, Sayuri Takayama, Nobuyuki Matsuda, Hirofumi Aiba, Hisashi Hemmi, and Tohru Yoshimura. The implication of yggt of escherichia coli in osmotic regulation. *Bioscience, biotechnology, and biochemistry*, 73(12):2698–2704, 2009.

[48] Roy D Sleator and Colin Hill. Bacterial osmoadaptation: the role of osmolytes in bacterial stress and virulence. *FEMS Microbiology Reviews*, 26(1):49–71, 2002.

[49] Chynna N Broxton and Valeria C Culotta. Sod enzymes and microbial pathogens: surviving the oxidative storm of infection. *PLoS Pathogens*, 12(1):e1005295, 2016.

[50] A Battistoni. Role of prokaryotic cu, zn superoxide dismutase in pathogenesis. *Biochemical Society Transactions*, 31(6):1326–1329, 2003.

[51] Robert J Cybulski Jr, Patrick Sanz, Farhang Alem, Scott Stibitz, Robert L Bull, and Alison D O'Brien. Four superoxide dismutases contribute to bacillus anthracis virulence and provide spores with redundant protection from oxidative stress. *Infection and immunity*, 77(1):274–285, 2009.

[52] Debra L Piddington, Ferric C Fang, Tracey Laessig, Andrea M Cooper, Ian M Orme, and Nancy A Buchmeier. Cu, zn superoxide dismutase of mycobacterium tuberculosis contributes to survival in activated macrophages that are generating an oxidative burst. *Infection and immunity*, 69(8):4980–4987, 2001.

[53] Yumi Iwadate, Noriyuki Funabasama, and Jun-ichi Kato. Involvement of formate dehydrogenases in stationary phase oxidative stress tolerance in escherichia coli. *FEMS Microbiology Letters*, 364(20):fnx193, 2017.

[54] Inga Oberpichler, Antonio J Pierik, Janine Wesslowski, Richard Pokorny, Ran Rosen, Michal Vugman, Fan Zhang, Olivia Neubauer, Eliora Z Ron, Alfred Batschauer, et al. A photolyase-like protein from agrobacterium tumefaciens with an iron-sulfur cluster. *PLoS One*, 6(10):e26775, 2011.

[55] Eun-Jeong Yoon, Jung Ok Kim, Ji Woo Yang, Hwa Su Kim, Kwang Jun Lee, Seok Hoon Jeong, Hyukmin Lee, and Kyungwon Lee. The bla oxa-23-associated transposons in the genome of acinetobacter spp. represent an epidemiological situation of the species encountering carbapenems. *Journal of Antimicrobial Chemotherapy*, 72(10):2708–2714, 2017.

[56] Anton Y Peleg, Harald Seifert, and David L Paterson. Acinetobacter baumannii: emergence of a successful pathogen. *Clinical Microbiology Reviews*, 21(3):538–582, 2008.

[57] Katherine E Bowden, Michael R Weigand, Yanhui Peng, Pamela K Cassiday, Scott Sammons, Kristen Knipe, Lori A Rowe, Vladimir Loparev, Mili Sheth, Keeley Weening, et al. Genome structural diversity among 31 bordetella pertussis isolates from two recent us whooping cough statewide epidemics. *MSphere*, 1(3):e00036–16, 2016.

[58] Derek S Sarovich, Jessica R Webb, Matthew C Pitman, Linda T Viberg, Mark Mayo, Robert W Baird, Jennifer M Robson, Bart J Currie, and Erin P Price. Raising the stakes: loss of efflux pump regulation decreases meropenem susceptibility in burkholderia pseudomallei. *Clinical Infectious Diseases*, 67(2):243–250, 2018.

[59] Mihnea R Mangalea, Brooke A Plumley, and Bradley R Borlee. Nitrate sensing and metabolism inhibit biofilm formation in the opportunistic pathogen burkholderia pseudomallei by reducing the intracellular concentration of c-di-gmp. *Frontiers in Microbiology*, 8:1353, 2017.

[60] Valentina A Feodorova, Sergey S Zaitsev, Mariya A Khizhnyakova, Yury V Saltykov, Vitaliy V Evstifeev, Fidail M Khusainov, Sergey I Yakovlev, Olga S Larionova, and Vladimir L Motin. Data of de novo genome assembly of the chlamydia psittaci strain isolated from the livestock in volga region, russian federation. *Data in Brief*, 29:105190, 2020.

[61] Robert J Suchland, Zoe E Dimond, Timothy E Putman, and Daniel D Rockey. Demonstration of persistent infections and genome stability by whole-genome sequencing of repeat-positive, same-serovar chlamydia trachomatis collected from the female genital tract. *The Journal of Infectious Diseases*, 215(11):1657–1665, 2017.

[62] PATRIC Database. Citrobacter koseri strain FDAARGOS_287, Genome ID 545.38. `https://www.patricbrc.org/view/Genome/545.38`.

[63] Chao Yuan, Zhiqiu Yin, Junyue Wang, Chengqian Qian, Yi Wei, Si Zhang, Lingyan Jiang, and Bin Liu. Comparative genomic analysis of citrobacter and key genes essential for the pathogenicity of citrobacter koseri. *Frontiers in Microbiology*, 10:2774, 2019.

[64] Runa Kuley, Eric Kuijt, Mari A Smits, Hendrik IJ Roest, Hilde E Smith, and Alex Bossers. Genome plasticity and polymorphisms in critical genes correlate with increased virulence of dutch outbreak-related coxiella burnetii strains. *Frontiers in Microbiology*, 8:1526, 2017.

[65] Andrew H Buultjens, Margaret MC Lam, Susan Ballard, Ian R Monk, Andrew A Mahony, Elizabeth A Grabsch, M Lindsay Grayson, Stanley Pang, Geoffrey W Coombs, J Owen Robinson,

et al. Evolutionary origins of the emergent st796 clone of vancomycin resistant enterococcus faecium. *PeerJ*, 5:e2916, 2017.

[66] Xuewei Zhou, Rob JL Willems, Alexander W Friedrich, John WA Rossen, and Erik Bathoorn. Enterococcus faecium: from microbiological insights to practical recommendations for infection control and diagnostics. *Antimicrobial Resistance & Infection Control*, 9(1):1–13, 2020.

[67] PATRIC Database. Escherichia coli strain FDAARGOS_433, Genome ID 562.22306. https://www.patricbrc.org/view/Genome/562.22306.

[68] Kenji K Kojima, Yoshikazu Furuta, Koji Yahara, Masaki Fukuyo, Yuh Shiwa, Shin Nishiumi, Masaru Yoshida, Takeshi Azuma, Hirofumi Yoshikawa, and Ichizo Kobayashi. Population evolution of helicobacter pylori through diversification in dna methylation and interstrain sequence homogenization. *Molecular Biology and Evolution*, 33(11):2848–2859, 2016.

[69] PATRIC Database. Klebsiella pneumoniae strain KP9, Genome ID 573.16474. https://www.patricbrc.org/view/Genome/573.16474.

[70] Xiaohui Chi, Björn Berglund, Huiyun Zou, Beiwen Zheng, Stefan Börjesson, Xiang Ji, Jakob Ottoson, Cecilia Stålsby Lundborg, Xuewen Li, and Lennart E Nilsson. Characterization of clinically relevant strains of extended-spectrum β-lactamase-producing klebsiella pneumoniae occurring in environmental sources in a rural area of china by using whole-genome sequencing. *Frontiers in Microbiology*, 10:211, 2019.

[71] Francis Muchaamba, Claudia Guldimann, Taurai Tasara, María Inés Mota, Valeria Braga, Gustavo Varela, Gabriela Algorta, Jochen Klumpp, Marco Jermini, and Roger Stephan. Full-genome sequence of listeria monocytogenes strain h34, isolated from a newborn with sepsis in uruguay. *Genome announcements*, 5(24):e00544–17, 2017.

[72] MN Gonzalez-Perez, MI Murcia, C Parra-Lopez, J Blom, and Andreas Tauch. Deciphering the virulence factors of the opportunistic pathogen mycobacterium colombiense. *New Microbes and New Infections*, 14:98–105, 2016.

[73] Hanako Fukano, Mitsunori Yoshida, Yuko Kazumi, Nagatoshi Fujiwara, Kinya Katayama, Yoshitoshi Ogura, Tetsuya Hayashi, Yuji Miyamoto, Noriki Fujimoto, Wang Hongsheng, et al. Mycobacterium shigaense sp. nov., a slow-growing, scotochromogenic species, is a member of the mycobacterium simiae complex. *International Journal of Systematic and Evolutionary Microbiology*, 68(8):2437–2442, 2018.

[74] H Fukano, O Hiranuma, Y Matsui, S Tanaka, and Y Hoshino. The first case of chronic pulmonary mycobacterium shigaense infection in an immunocompetent patient. *New Microbes and New Infections*, 33:100630, 2020.

[75] Patricia Sheen, David Requena, Eduardo Gushiken, Robert H Gilman, Ricardo Antiparra, Bryan Lucero, Pilar Lizárraga, Basilio Cieza, Elisa Roncal, Louis Grandjean, et al. A multiple genome

analysis of mycobacterium tuberculosis reveals specific novel genes and mutations associated with pyrazinamide resistance. *BMC Genomics*, 18(1):769, 2017.

[76] Tsuyoshi Kenri, Masato Suzuki, Atsuko Horino, Tsuyoshi Sekizuka, Makoto Kuroda, Hiroyuki Fujii, Toru Hashimoto, Hiroshi Nakajima, Hitomi Ohya, and Keigo Shibayama. Complete genome sequences of the p1 gene type 2b and 2c strains mycoplasma pneumoniae kch-402 and kch-405. *Genome announcements*, 5(24):e00513–17, 2017.

[77] PATRIC Database. Nocardia brasiliensis strain FDAARGOS_352, Genome ID 37326.9. `https://www.patricbrc.org/view/Genome/37326.9`.

[78] Barbara A Brown-Elliott, June M Brown, Patricia S Conville, and Richard J Wallace. Clinical and laboratory features of the nocardia spp. based on current molecular taxonomy. *Clinical Microbiology Reviews*, 19(2):259–282, 2006.

[79] Mariko Naito, Yoshitoshi Ogura, Takehiko Itoh, Mikio Shoji, Masaaki Okamoto, Tetsuya Hayashi, and Koji Nakayama. The complete genome sequencing of prevotella intermedia strain oma14 and a subsequent fine-scale, intra-species genomic comparison reveal an unusual amplification of conjugative and mobile transposons and identify a novel prevotella-lineage-specific repeat. *DNA Research*, 23(1):11–19, 2016.

[80] Simone G Santos, Cláudio G Diniz, Vânia L Silva, Francisca L Lima, Hélida M Andrade, Donat A Chapeaurouge, Jonas Perales, José Carlos Serufo, Maria Auxiliadora R Carvalho, and Luiz M Farias. Differentially regulated proteins in prevotella intermedia after oxidative stress analyzed by 2d electrophoresis and mass spectrometry. *Anaerobe*, 18(1):76–82, 2012.

[81] Weihua Huang, Joelle El Hamouche, Guiqing Wang, Melissa Smith, Changhong Yin, Abhay Dhand, Nevenka Dimitrova, and John T Fallon. Integrated genome-wide analysis of an isogenic pair of pseudomonas aeruginosa clinical isolates with differential antimicrobial resistance to ceftolozane/tazobactam, ceftazidime/avibactam, and piperacillin/tazobactam. *International Journal of Molecular Sciences*, 21(3):1026, 2020.

[82] Stephen P Diggle and Marvin Whiteley. Microbe profile: Pseudomonas aeruginosa: opportunistic pathogen and lab rat. *Microbiology*, 166(1):30–33, 2020.

[83] Artur J Sabat, Sandra M Hermelijn, Viktoria Akkerboom, Amadu Juliana, John E Degener, Hajo Grundmann, and Alexander W Friedrich. Complete-genome sequencing elucidates outbreak dynamics of ca-mrsa usa300 (st8-spa t008) in an academic hospital of paramaribo, republic of suriname. *Scientific Reports*, 7(1):1–12, 2017.

[84] James P O'Gara. Into the storm: Chasing the opportunistic pathogen staphylococcus aureus from skin colonisation to life-threatening infections. *Environmental Microbiology*, 19(10):3823–3833, 2017.

[85] PATRIC Database. Streptococcus gordonii strain FDAARGOS_257, Genome ID 1302.83. `https://www.patricbrc.org/view/Genome/1302.83`.

[86] Ok-Jin Park, Yeongkag Kwon, Chaeyeon Park, Yoon Ju So, Tae Hwan Park, Sungho Jeong, Jintaek Im, Cheol-Heui Yun, and Seung Hyun Han. Streptococcus gordonii: Pathogenesis and host response to its cell wall components. *Microorganisms*, 8(12):1852, 2020.

[87] Elio Issa, Tamara Salloum, Balig Panossian, David Ayoub, Edmond Abboud, and Sima Tokajian. Genome mining and comparative analysis of streptococcus intermedius causing brain abscess in a child. *Pathogens*, 8(1):22, 2019.

[88] Yoshio Kondo, Yoshitoshi Ogura, Keiko Sato, Keigo Imamura, Tomonori Hoshino, Miyuki Nishiguchi, Tomoyuki Hasuwa, Hiroyuki Moriuchi, Tetsuya Hayashi, and Taku Fujiwara. Complete genome sequence of streptococcus sp. strain nps 308. *Genome announcements*, 4(6):e01349–16, 2016.

[89] PATRIC Database. Haemophilus ducreyi strain FDAARGOS_297, Genome ID 730.54. `https://www.patricbrc.org/view/Genome/730.54`.

[90] Michelle Alfa. The laboratory diagnosis of haemophilus ducreyi. *Canadian Journal of Infectious Diseases and Medical Microbiology*, 16(1):31–34, 2005.

[91] J Kang, W-H Chung, T-J Lim, S Lim, and Y-D Nam. Complete genome sequence of the bifidobacterium animalis subspecies lactis bl3, preventive probiotics for acute colitis and colon cancer. *New Microbes and New Infections*, 19:34–37, 2017.

[92] PATRIC Database. Clostridioides difficile strain BR81, Genome ID 1496.1556. `https://www.patricbrc.org/view/Genome/1496.1556`.

[93] Mehdi Goudarzi, Sima Sadat Seyedjavadi, Hossein Goudarzi, Elnaz Mehdizadeh Aghdam, and Saeed Nazeri. Clostridium difficile infection: epidemiology, pathogenesis, risk factors, and therapeutic options. *Scientifica*, 2014, 2014.

[94] Satyabrata Bag, Tarini Shankar Ghosh, and Bhabatosh Das. Complete genome sequence of collinsella aerofaciens isolated from the gut of a healthy indian subject. *Genome announcements*, 5(47):e01361–17, 2017.

[95] Dieter M Tourlousse, Mitsuo Sakamoto, Takamasa Miura, Koji Narita, Akiko Ohashi, Yoshihito Uchino, Atsushi Yamazoe, Keishi Kameyama, Jun Terauchi, Moriya Ohkuma, et al. Complete genome sequence of collinsella aerofaciens jcm 10188t. *Microbiology resource announcements*, 9(16):e00134–20, 2020.

[96] Satyabrata Bag, Tarini Shankar Ghosh, and Bhabatosh Das. Complete genome sequence of faecalibacterium prausnitzii isolated from the gut of a healthy indian adult. *Genome announcements*, 5(46):e01286–17, 2017.

[97] II Ngom, I Hasni, CI Lo, SI Traore, A Fontanini, D Raoult, and F Fenollar. Taxono-genomics and description of gordonibacter massiliensis sp. nov., a new bacterium isolated from stool of healthy patient. *New Microbes and New Infections*, 33:100624, 2020.

[98] Jisu Kang, Won-Hyong Chung, Tae-Joong Lim, Tae Woong Whon, Sanghyun Lim, and Young-Do Nam. Complete genome sequence of lactobacillus casei lc5, a potential probiotics for atopic dermatitis. *Frontiers in Immunology*, 8:413, 2017.

[99] Huey-Shi Lye, Tamotsu Kato, Wai-Yee Low, Todd D Taylor, Tulika Prakash, Lee-Ching Lew, Hiroshi Ohno, and Min-Tze Liong. Lactobacillus fermentum ftdc 8312 combats hypercholesterolemia via alteration of gut microbiota. *Journal of Biotechnology*, 262:75–83, 2017.

[100] Zitong Ding, Anum Hani, Wenyuan Li, Wencan Ke, Xusheng Guo, et al. Influence of a cholesterol-lowering strain lactobacillus plantarum lp3 isolated from traditional fermented yak milk on gut bacterial microbiota and metabolome of rats fed with a high-fat diet. *Food & Function*, 11(9):8342–8353, 2020.

[101] Piotr Jarocki, Marcin Podleśny, Mariusz Krawczyk, Agnieszka Glibowska, Jarosław Pawelec, Elwira Komoń-Janczara, Oleksandr Kholiavskyi, Michał Dworniczak, and Zdzisław Targoński. Complete genome sequence of lactobacillus rhamnosus pen, a probiotic component of a medicine used in prevention of antibiotic-associated diarrhoea in children. *Gut Pathogens*, 10(1):1–6, 2018.

[102] Giulia Bernardini, Giovanni Renzone, Maurizio Comanducci, Roberta Mini, Simona Arena, Chiara D'Ambrosio, Stefania Bambini, Lorenza Trabalzini, Guido Grandi, Paola Martelli, et al. Proteome analysis of neisseria meningitidis serogroup a. *Proteomics*, 4(10):2893–2926, 2004.

[103] M Bilen, F Cadoret, G Dubourg, P-E Fournier, Z Daoud, and Didier Raoult. 'phoenicibacter massiliensis' gen. nov., sp. nov., a new bacterium isolated from the human gut of a pygmy woman. *New Microbes and New Infections*, 19:43–44, 2017.

[104] Om Prakash, Suchismita Muduli, Rohit Kumar, Chanchal Kumari, Yogesh Nimonkar, Yogesh S Shouche, and Rakesh Sharma. Description of auricoccus indicus gen. nov., sp. nov., isolated from skin of human ear. *International Journal of Systematic and Evolutionary Microbiology*, 67(5):1212–1218, 2017.

[105] Voula Alexandraki, Maria Kazou, Jochen Blom, Bruno Pot, Konstantinos Papadimitriou, and Effie Tsakalidou. Comparative genomics of streptococcus thermophilus support important traits concerning the evolution, biology and technological properties of the species. *Frontiers in Microbiology*, 10:2916, 2019.

[106] Mi-Sun Kang, Hee-Sam Na, and Jong-Suk Oh. Coaggregation ability of weissella cibaria isolates with fusobacterium nucleatum and their adhesiveness to epithelial cells. *FEMS Microbiology Letters*, 253(2):323–329, 2005.

[107] Michael A Mahowald, Federico E Rey, Henning Seedorf, Peter J Turnbaugh, Robert S Fulton, Aye Wollam, Neha Shah, Chunyan Wang, Vincent Magrini, Richard K Wilson, et al. Characterizing a model human gut microbiota composed of members of its two dominant bacterial phyla. *Proceedings of the National Academy of Sciences*, 106(14):5859–5864, 2009.

[108] PATRIC Database. Burkholderia pseudomallei strain 2010007509, Genome ID 28450.655. https://www.patricbrc.org/view/Genome/28450.655.

[109] Atsushi Iguchi, Nicholas R Thomson, Yoshitoshi Ogura, David Saunders, Tadasuke Ooka, Ian R Henderson, David Harris, M Asadulghani, Ken Kurokawa, Paul Dean, et al. Complete genome sequence and comparative genome analysis of enteropathogenic escherichia coli o127: H6 strain e2348/69. *Journal of Bacteriology*, 191(1):347–354, 2009.

[110] PATRIC Database. Escherichia coli strain 5CRE51, Genome ID 562.16466. https://www.patricbrc.org/view/Genome/562.16466.

[111] PATRIC Database. Escherichia coli strain ATCC 43896, Genome ID 562.22333. https://www.patricbrc.org/view/Genome/562.22333.

[112] PATRIC Database. Escherichia coli strain Ecol_276, Genome ID 562.15193. https://www.patricbrc.org/view/Genome/562.15193.

[113] PATRIC Database. Escherichia coli strain FDAARGOS_434, Genome ID 562.22323. https://www.patricbrc.org/view/Genome/562.22323.

[114] PATRIC Database. Escherichia coli strain FDAARGOS_448, Genome ID 562.22307. https://www.patricbrc.org/view/Genome/562.22307.

[115] Timothy J Johnson, Jessica L Danzeisen, Bonnie Youmans, Kyle Case, Katharine Llop, Jeannette Munoz-Aguayo, Cristian Flores-Figueroa, Maliha Aziz, Nicole Stoesser, Evgeni Sokurenko, et al. Separate f-type plasmids have shaped the evolution of the h 30 subclone of escherichia coli sequence type 131. *MSphere*, 1(4):e00121–16, 2016.

[116] PATRIC Database. Escherichia coli strain UFU_EC98, Genome ID 562.22326. https://www.patricbrc.org/view/Genome/562.22326.

[117] PATRIC Database. Klebsiella pneumoniae strain 459, Genome ID 573.16440. https://www.patricbrc.org/view/Genome/573.16440.

[118] PATRIC Database. Klebsiella pneumoniae strain FDAARGOS_436, Genome ID 573.15319. https://www.patricbrc.org/view/Genome/573.15319.

[119] PATRIC Database. Listeria monocytogenes serotype 4b str. 02-1289 strain 02-1289, Genome ID 1196172.3. https://www.patricbrc.org/view/Genome/1196172.3.

[120] PATRIC Database. Listeria monocytogenes serotype 4b str. 10-0809, Genome ID 1196162.3. https://www.patricbrc.org/view/Genome/1196162.3.

[121] Temitope A Folaranmi, Cecilia B Kretz, Hajime Kamiya, Jessica R MacNeil, Melissa J Whaley, Amy Blain, Mike Antwi, Marie Dorsinville, Massimo Pacilli, Shamika Smith, et al. Increased risk for meningococcal disease among men who have sex with men in the united states, 2012–2015. *Clinical Infectious Diseases*, 65(5):756–763, 2017.

[122] João Botelho, Filipa Grosso, Sandra Quinteira, Michael Brilhante, Helena Ramos, and Luísa Peixe. Two decades of bla vim-2-producing pseudomonas aeruginosa dissemination: an interplay between mobile genetic elements and successful clones. *Journal of Antimicrobial Chemotherapy*, 73(4):873–882, 2018.

[123] PATRIC Database. Staphylococcus aureus strain JE2, Genome ID 1280.12234. `https://www.patricbrc.org/view/Genome/1280.12234`.

[124] PATRIC Database. Staphylococcus aureus strain USA300-SUR11, Genome ID 1280.11677. `https://www.patricbrc.org/view/Genome/1280.11677`.

[125] Tor Hofstad. The genus fusobacterium. In *The prokaryotes*, pages 4114–4126. Springer, 1992.

[126] Jie Gao, Yubin Li, Yu Wan, Tongtong Hu, Liting Liu, Shaojie Yang, Zelong Gong, Qing Zeng, Yi Wei, Weijun Yang, et al. A novel postbiotic from lactobacillus rhamnosus gg with a beneficial effect on intestinal barrier function. *Frontiers in Microbiology*, 10:477, 2019.

[127] ME Trujillo and M Goodfellow. Polyphasic taxonomic study of clinically significant actinomadurae including the description of actinomadura latina sp. nov. *Zentralblatt für Bakteriologie*, 285(2):212–233, 1997.

[128] Lingtong Tang, Jianglian Huang, Junping She, Kelei Zhao, and Yingshun Zhou. Co-occurrence of the bla kpc-2 and mcr-3.3 gene in aeromonas caviae scac2001 isolated from patients with diarrheal disease. *Infection and Drug Resistance*, 13:1527–1536, 2020.

[129] Petros Ioannou. Escherichia hermannii infections in humans: a systematic review. *Tropical Medicine and Infectious Disease*, 4(1):17, 2019.

[130] Zhenjun Li, Xu-ming Wang, Xiong Zhu, Miao Wang, Hai Cheng, Dan Li, and Zhi Guo Liu. Molecular characteristics of brucella isolates collected from humans in hainan province, china. *Frontiers in Microbiology*, 11:452, 2020.

[131] Inês N Silva, Pedro M Santos, Mário R Santos, James EA Zlosnik, David P Speert, Sean W Buskirk, Eric L Bruger, Christopher M Waters, Vaughn S Cooper, and Leonilde M Moreira. Long-term evolution of burkholderia multivorans during a chronic cystic fibrosis infection reveals shifting forces of selection. *MSystems*, 1(3):e00029–16, 2016.

[132] Samuel K Sheppard and Martin CJ Maiden. The evolution of campylobacter jejuni and campylobacter coli. *Cold Spring Harbor perspectives in biology*, 7(8):a018119, 2015.

[133] PATRIC Database. Campylobacter coli strain 202823, Genome ID 195.2778. `https://www.patricbrc.org/view/Genome/195.2778`.

[134] Aboi Igwaran and Anthony Ifeanyi Okoh. Human campylobacteriosis: A public health concern of global importance. *Heliyon*, 5(11):e02814, 2019.

[135] PATRIC Database. Campylobacter lari strain 503734, Genome ID 201.69. https://www.patricbrc.org/view/Genome/201.69.

[136] PATRIC Database. Campylobacter sp. CFSAN093243, Genome ID 2572066.4. https://www.patricbrc.org/view/Genome/2572066.4.

[137] Veronica Bravo, Assaf Katz, Lorena Porte, Thomas Weitzel, Carmen Varela, Narjol Gonzalez-Escalona, and Carlos J Blondel. Genomic analysis of the diversity, antimicrobial resistance and virulence potential of clinical campylobacter jejuni and campylobacter coli strains from chile. *PLOS Neglected Tropical Diseases*, 15(2):e0009207, 2021.

[138] Michael W Peck and Arnoud HM van Vliet. Impact of clostridium botulinum genomic diversity on food safety. *Current Opinion in Food Science*, 10:52–59, 2016.

[139] Justine Schaeffer, Steliana Huhulescu, Anna Stoeger, Franz Allerberger, and Werner Ruppitsch. Draft genome sequences of six corynebacterium ulcerans strains isolated from humans and animals in austria, 2013 to 2019. *Microbiology Resource Announcements*, 9(36):e00946–20, 2020.

[140] Tadasuke Ooka, Eisuke Tokuoka, Masato Furukawa, Tetsuya Nagamura, Yoshitoshi Ogura, Kokichi Arisawa, Seiya Harada, and Tetsuya Hayashi. Human gastroenteritis outbreak associated with escherichia albertii, japan. *Emerging Infectious Diseases*, 19(1):144, 2013.

[141] Christiana Jesumirhewe, Burkhard Springer, Franz Allerberger, and Werner Ruppitsch. Whole genome sequencing of extended-spectrum β-lactamase genes in enterobacteriaceae isolates from nigeria. *PloS One*, 15(4):e0231146, 2020.

[142] Rajagopalan Saranathan, Michael H Levi, Alice R Wattam, Adel Malek, Emmanuel Asare, Daniel S Behin, Debra H Pan, William R Jacobs Jr, and Wendy A Szymczak. Helicobacter pylori infections in the bronx, new york: surveying antibiotic susceptibility and strain lineage by whole-genome sequencing. *Journal of clinical microbiology*, 58(3):e01591–19, 2020.

[143] Freddy Haesebrouck, Frank Pasmans, Bram Flahou, Koen Chiers, Margo Baele, Tom Meyns, Annemie Decostere, and Richard Ducatelle. Gastric helicobacters in domestic animals and nonhuman primates and their significance for human health. *Clinical Microbiology Reviews*, 22(2):202–223, 2009.

[144] Camilla NR Trindade, Pedro HN Panzenhagen, Ricardo M Junqueira, Deyse CV Silva, Carlos A Conte-Junior, and Ilana T Balassiano. Draft genome sequences of leptospira interrogans serovar copenhageni strains isolated from patients with weil's disease in brazil. *Microbiology resource announcements*, 9(7):e01538–19, 2020.

[145] Vidhya Ramaswamy, Vincent Mary Cresence, Jayan S Rejitha, Mohandas Usha Lekshmi, KS Dharsana, Suryaprasad Priya Prasad, and Helan Mary Vijila. Listeria-review of epidemiology and pathogenesis. *Journal of Microbiology Immunology and Infection*, 40(1):4, 2007.

[146] Philip Supply and Roland Brosch. The biology and epidemiology of mycobacterium canettii. In *Strain Variation in the Mycobacterium tuberculosis Complex: Its Role in Biology, Epidemiology and Control*, pages 27–41. Springer, 2017.

[147] Tomasz Jagielski, Paulina Borówka, Zofia Bakuła, Jakub Lach, Błażej Marciniak, Anna Brzostek, Jarosław Dziadek, Mikołaj Dziurzyński, Lian Pennings, Jakko Van Ingen, et al. Genomic insights into the mycobacterium kansasii complex: an update. *Frontiers in Microbiology*, 10:2918, 2020.

[148] Aymen Abdelhaleem, Almonther Hershan, Pradeep Agarwal, Abdullah Farasani, Shaheed V Omar, Arshad Ismail, and Mushal Allam. Whole-genome sequencing of a mycobacterium tuberculosis strain belonging to lineage 1 (indo-oceanic) and the east african indian spoligotype, isolated in jazan, saudi arabia. *Microbiology Resource Announcements*, 9(33):e00717–20, 2020.

[149] Daria Bottai, Wafa Frigui, Fadel Sayes, Mariagrazia Di Luca, Dalila Spadoni, Alexandre Pawlik, Marina Zoppo, Mickael Orgeur, Varun Khanna, David Hardy, et al. Tbd1 deletion as a driver of the evolutionary success of modern epidemic mycobacterium tuberculosis lineages. *Nature Communications*, 11(1):1–14, 2020.

[150] Sarah Jane Quillin and H Steven Seifert. Neisseria gonorrhoeae host adaptation and pathogenesis. *Nature Reviews Microbiology*, 16(4):226, 2018.

[151] Arif M Tanmoy, Emilie Westeel, Katrien De Bruyne, Johan Goris, Alain Rajoharison, Mohammad SI Sajib, Alex van Belkum, Samir K Saha, Florence Komurian-Pradel, and Hubert P Endtz. Salmonella enterica serovar typhi in bangladesh: exploration of genomic diversity and antimicrobial resistance. *MBio*, 9(6):e02112–18, 2018.

[152] Ahtesham Ahmad Shad and Wajahat Ahmed Shad. Shigella sonnei: virulence and antibiotic resistance. *Archives of Microbiology*, 203(1):45–58, 2021.

[153] Mike S Son, Christina J Megli, Gabriela Kovacikova, Firdausi Qadri, and Ronald K Taylor. Characterization of vibrio cholerae o1 el tor biotype variant clinical isolates from bangladesh and haiti, including a molecular genetic analysis of virulence genes. *Journal of Clinical Microbiology*, 49(11):3739–3749, 2011.

[154] Marta Nieckarz, Patrycja Kaczor, Karolina Jaworska, Adrianna Raczkowska, and Katarzyna Brzostek. Urease expression in pathogenic yersinia enterocolitica strains of bio-serotypes 2/o: 9 and 1b/o: 8 is differentially regulated by the ompr regulator. *Frontiers in Microbiology*, 11:607, 2020.

[155] Julia Dai, Auris O Huen, Lori A Kestenbaum, Margaret D Sarezky, Carrie C Coughlin, and Albert C Yan. Achromobacter xylosoxidans bacteremia and cellulitis: a report of a case. *Pediatric Dermatology*, 32(4):e186–e187, 2015.

[156] Aoife Howard, Michael O'Donoghue, Audrey Feeney, and Roy D Sleator. Acinetobacter baumannii: an emerging opportunistic pathogen. *Virulence*, 3(3):243–250, 2012.

[157] Semiramis Castro-Jaimes, Elena Bello-López, Consuelo Velázquez-Acosta, Patricia Volkow-Fernández, Patricia Lozano-Zarain, Santiago Castillo-Ramírez, and Miguel Angel Cevallos. Chromosome architecture and gene content of the emergent pathogen acinetobacter haemolyticus. *Frontiers in Microbiology*, 11:926, 2020.

[158] Nora G Regalado, Greg Martin, and Suresh J Antony. Acinetobacter lwoffii: bacteremia associated with acute gastroenteritis. *Travel Medicine and Infectious Disease*, 7(5):316–317, 2009.

[159] P Espinal, H Seifert, L Dijkshoorn, J Vila, and I Roca. Rapid and accurate identification of genomic species from the acinetobacter baumannii (ab) group by maldi-tof ms. *Clinical Microbiology and Infection*, 18(11):1097–1103, 2012.

[160] PATRIC Database. Bacillus mobilis strain 1428.155, Genome ID 2026190.21. `https://www.patricbrc.org/view/Genome/2026190.21`.

[161] Nalini Ramarao, Seav-Ly Tran, Marco Marin, and Jasmina Vidic. Advanced methods for detection of bacillus cereus and its pathogenic factors. *Sensors*, 20(9):2667, 2020.

[162] M Jeremiah Matson, Sarah L Anzick, Friederike Feldmann, Craig A Martens, Steven K Drake, Heinz Feldmann, Moses Massaquoi, Daniel S Chertow, and Vincent J Munster. Bacillus paranthracis isolate from blood of fatal ebola virus disease case. *Pathogens*, 9(6):475, 2020.

[163] Hannah M Wexler. Bacteroides: the good, the bad, and the nitty-gritty. *Clinical Microbiology Reviews*, 20(4):593–621, 2007.

[164] Magdolna Csávás, Lenka Malinovská, Florent Perret, Milán Gyurkó, Zita Tünde Illyés, Michaela Wimmerová, and Anikó Borbás. Tri-and tetravalent mannoclusters cross-link and aggregate bc2l-a lectin from burkholderia cenocepacia. *Carbohydrate Research*, 437:1–8, 2017.

[165] Eshwar Mahenthiralingam, A Baldwin, and CG Dowson. Burkholderia cepacia complex bacteria: opportunistic pathogens with important natural biology. *Journal of Applied Microbiology*, 104(6):1539–1551, 2008.

[166] Pengling Li, Min Wang, Xianping Li, Feihu Hu, Huan Song, Yixin Xie, Min Yang, and Aiguo Tang. Case report spontaneous bacterial peritonitis caused by oxidase negative campylobacter fetus subsp. testudinum isolated from the patient with decompensated liver cirrhosis: a case report and literatures review. *Int J Clin Exp Med*, 9(7):14866–14869, 2016.

[167] Jun Hirai, Kohei Uechi, Mao Hagihara, Daisuke Sakanashi, Takeshi Kinjo, Shusaku Haranaga, and Jiro Fujita. Bacteremia due to citrobacter braakii: a case report and literature review. *Journal of Infection and Chemotherapy*, 22(12):819–821, 2016.

[168] Ahmed Babiker, Daniel R Evans, Marissa P Griffith, Christi L McElheny, Mohamed Hassan, Lloyd G Clarke, Roberta T Mettus, Lee H Harrison, Yohei Doi, Ryan K Shields, et al. Clinical and genomic epidemiology of carbapenem-nonsusceptible citrobacter spp. at a tertiary health care center over 2 decades. *Journal of clinical microbiology*, 58(9):e00275–20, 2020.

[169] Yukino Usui, Yoko Nukui, Ryuji Koike, Shuji Tohda, and Ryoichi Saito. Draft genome sequence of a clostridioides difficile sequence type 97 strain belonging to hypervirulent clade 2. *Microbiology resource announcements*, 9(14):e00245–20, 2020.

[170] Niharika Singh, Gunjan Goel, and Mamta Raghav. Insights into virulence factors determining the pathogenicity of cronobacter sakazakii. *Virulence*, 6(5):433–440, 2015.

[171] Michael Hombach, Hans R Frey, and Gaby E Pfyffer. Urinary tract infection caused by eikenella corrodens. *Journal of Clinical Microbiology*, 45(2):675–675, 2007.

[172] PATRIC Database. Eikenella corrodens strain EI_09, Genome ID 539.170. `https://www.patricbrc.org/view/Genome/539.170`.

[173] Kristin A Stormo, Randi M Nygaard, Torbjørn S Bruvold, Grete Dimmen, Paul Christoffer Lindemann, Stina Jordal, and Øyvind Kommedal. Eikenella exigua sp. nov., isolated from brain abscess and blood. *International Journal of Systematic and Evolutionary Microbiology*, 70(3):1478–1488, 2020.

[174] Lisa R McTaggart, Patrick J Stapleton, AliReza Eshaghi, Deirdre Soares, Sylvain Brisse, Samir N Patel, and Julianne V Kus. Application of whole genome sequencing to query a potential outbreak of elizabethkingia anophelis in ontario, canada. *Access Microbiology*, 1(2):e000017, 2019.

[175] Medini K Annavajhala, Angela Gomez-Simmonds, and Anne-Catrin Uhlemann. Multidrug-resistant enterobacter cloacae complex emerging as a global, diversifying threat. *Frontiers in Microbiology*, 10:44, 2019.

[176] Wenjing Wu, Yu Feng, and Zhiyong Zong. Characterization of a strain representing a new enterobacter species, enterobacter chengduensis sp. nov. *Antonie van Leeuwenhoek*, 112(4):491–500, 2019.

[177] Akira Uchida, Hie Lim Kim, Rikky W Purbojati, Vineeth Kodengil Vettath, Anjali B Gupta, James NI Houghton, Caroline Chénard, Anthony Wong, Megan E Clare, Kavita K Kushwaha, et al. Complete genome of enterobacter sichuanensis strain SGAir0282 isolated from air in singapore. *Gut Pathogens*, 12(1):1–7, 2020.

[178] Elizabeth Fiore, Daria Van Tyne, and Michael S Gilmore. Pathogenicity of enterococci. *Microbiology spectrum*, 7(4):7–4, 2019.

[179] PATRIC Database. Enterococcus faecalis strain VNMU281, Genome ID 1351.3480. `https://www.patricbrc.org/view/Genome/1351.3480`.

[180] Jacopo Monticelli, Anna Knezevich, Roberto Luzzati, and Stefano Di Bella. Clinical management of non-faecium non-faecalis vancomycin-resistant enterococci infection. focus on enterococcus gallinarum andáenterococcus casseliflavus/flavescens. *Journal of Infection and Chemotherapy*, 24(4):237–246, 2018.

[181] PATRIC Database. Enterococcus gallinarum strain EGR748, Genome ID 1353.134. https://www.patricbrc.org/view/Genome/1353.134.

[182] Fahimeh Shooraj, Bahman Mirzaei, Seyed Fazlollah Mousavi, and Farzaneh Hosseini. Clonal diversity of haemophilus influenzae carriage isolated from under the age of 6 years children. *BMC Research Notes*, 12(1):565, 2019.

[183] Ad C Fluit, Miquel B Ekkelenkamp, Michael M Tunney, J Stuart Elborn, Malbert RC Rogers, and Jumamurat R Bayjanov. Draft genome sequence of a haemophilus parainfluenzae strain isolated from a patient with chronic obstructive pulmonary disease. *Microbiology resource announcements*, 9(13):e00079–20, 2020.

[184] Anne Davin-Regli et al. Enterobacter aerogenes and enterobacter cloacae; versatile bacterial pathogens confronting antibiotic treatment. *Frontiers in Microbiology*, 6:392, 2015.

[185] Paul Chapman, Brian M Forde, Leah W Roberts, Haakon Bergh, Debra Vesey, Amy V Jennison, Susan Moss, David L Paterson, Scott A Beatson, and Patrick NA Harris. Genomic investigation reveals contaminated detergent as the source of an extended-spectrum-β-lactamase-producing klebsiella michiganensis outbreak in a neonatal unit. *Journal of clinical microbiology*, 58(5):e01980–19, 2020.

[186] Lavan Singh, MP Cariappa, and Mandeep Kaur. Klebsiella oxytoca: An emerging pathogen? *Medical Journal Armed Forces India*, 72:S59–S61, 2016.

[187] Kazuo Imai, Noriomi Ishibashi, Masahiro Kodana, Norihito Tarumoto, Jun Sakai, Toru Kawamura, Shinichi Takeuchi, Yoshitada Taji, Yasuhiro Ebihara, Kenji Ikebuchi, et al. Clinical characteristics in blood stream infections caused by klebsiella pneumoniae, klebsiella variicola, and klebsiella quasipneumoniae: A comparative study, japan, 2014–2017. *BMC Infectious Diseases*, 19(1):946, 2019.

[188] Emenike RK Eribe and Ingar Olsen. Leptotrichia species in human infections ii. *Journal of Oral Microbiology*, 9(1):1368848, 2017.

[189] Luke V Blakeway, Aimee Tan, Ian RA Peak, and Kate L Seib. Virulence determinants of moraxella catarrhalis: distribution and considerations for vaccine development. *Microbiology*, 163(10):1371–1384, 2017.

[190] Hui Liu, Junmin Zhu, Qiwen Hu, and Xiancai Rao. Morganella morganii, a non-negligent opportunistic pathogen. *International Journal of Infectious Diseases*, 50:10–17, 2016.

[191] Jamal Saad, Nassim Hammoudi, Rita Zgheib, Hussein Anani, and Michel Drancourt. Geographic microevolution of mycobacterium ulcerans sustains buruli ulcer extension, australia. *bioRxiv*, 2020.

[192] Rose C Lopeman, James Harrison, Maya Desai, and Jonathan AG Cox. Mycobacterium abscessus: environmental bacterium turned clinical nightmare. *Microorganisms*, 7(3):90, 2019.

[193] Christiane Lourenço Nogueira, Keith E Simmon, Erica Chimara, Margo Cnockaert, Juan Carlos Palomino, Anandi Martin, Peter Vandamme, Barbara A Brown-Elliott, Richard J Wallace Jr, and Sylvia Cardoso Leao. Mycobacterium franklinii sp. nov., a species closely related to members of the mycobacterium chelonae–mycobacterium abscessus group. *International Journal of Systematic and Evolutionary Microbiology*, 65(7):2148–2153, 2015.

[194] Heer H Mehta and Yousif Shamoo. Pathogenic nocardia: A diverse genus of emerging pathogens or just poorly recognized? *PLoS Pathogens*, 16(3):e1008280, 2020.

[195] Yasutaka Hoshino, Kayo Watanabe, Soji Iida, Shin-ichi Suzuki, Takuji Kudo, Takahisa Kogure, Katsukiyo Yazawa, Jun Ishikawa, Reiner M Kroppenstedt, and Yuzuru Mikami. Nocardia terpenica sp. nov., isolated from japanese patients with nocardiosis. *International Journal of Systematic and Evolutionary Microbiology*, 57(7):1456–1460, 2007.

[196] Ma G Aguilera-Arreola, Martha L Ostria-Hernández, Enrique Albarrán-Fernández, Sara R Juárez-Enriquez, Cristina Majalca-Martínez, Beatríz Rico-Verdín, Enrico A Ruiz, María del Socorro Ruiz-Palma, María R Morales-García, and Araceli Contreras-Rodríguez. Correct identification of Ochrobactrum anthropi from blood culture using 16rRNA sequencing: a first case report in an immunocompromised patient in mexico. *Frontiers in Medicine*, 5:205, 2018.

[197] Peter Kämpfer, Holger C Scholz, Birgit Huber, Enevold Falsen, and Hans-Jürgen Busse. Ochrobactrum haematophilum sp. nov. and ochrobactrum pseudogrignonense sp. nov., isolated from human clinical specimens. *International Journal of Systematic and Evolutionary Microbiology*, 57(11):2513–2518, 2007.

[198] Ahmed Olowo-Okere, Yakubu Kokori Enevene Ibrahim, Busayo Olalekan Olayinka, Joseph Olorunmola Ehinmidu, Yahaya Mohammed, Larbi Zakaria Nabti, Jean-Marc Rolain, and Seydina M Diene. First whole genome sequence of paenalcaligenes suwonensis bearing blavim-5 metallo-β-lactamase: A clinical isolate responsible for acute gastroenteritis. *Infection, Genetics and Evolution*, 85:104513, 2020.

[199] Sanghamitra Padhi, Muktikesh Dash, Rani Sahu, and Pritilata Panda. Urinary tract infection due to paenibacillus alvei in a chronic kidney disease: a rare case report. *Journal of Laboratory Physicians*, 5(2):133, 2013.

[200] Zhongliang Zhu, Jiawen Lu, Ying Chen, and Fang He. Pasteurella canis infection caused by a dog bite leads to osteomyelitis and genomic analysis of the isolate. *Journal of Clinical Laboratory Analysis*, 34(7), 2020.

[201] Andrey Shelenkov, Lyudmila Petrova, Valeria Fomina, Mikhail Zamyatin, Yulia Mikhaylova, and Vasiliy Akimkin. Multidrug-resistant proteus mirabilis strain with cointegrate plasmid. *Microorganisms*, 8(11):1775, 2020.

[202] Dominika Drzewiecka. Significance and roles of proteus spp. bacteria in natural environments. *Microbial Ecology*, 72(4):741–758, 2016.

[203] Karima Warda, Loubna Ait Said, Khalid Zerouli, Khalid Katfy, and Kawtar Zahlane. First report of a providencia stuartii strain coproducing a beta-metalloenzyme NDM-1 type and an oxacillinase type OXA-48. *International Journal of Collaborative Research on Internal Medicine & Public Health*, 10(1):0–0, 2018.

[204] Jeffrey B Lyczak, Carolyn L Cannon, and Gerald B Pier. Establishment of pseudomonas aeruginosa infection: lessons from a versatile opportunist. *Microbes and Infection*, 2(9):1051–1060, 2000.

[205] PATRIC Database. Pseudomonas nitroreducens strain SC-1148-IPA-05, Genome ID 46680.55. `https://www.patricbrc.org/view/Genome/46680.55`.

[206] Brittan S Scales, Robert P Dickson, John J LiPuma, and Gary B Huffnagle. Microbiology, genomics, and clinical significance of the pseudomonas fluorescens species complex, an unappreciated colonizer of humans. *Clinical Microbiology Reviews*, 27(4):927–948, 2014.

[207] Annelise Senkowski. *Characterizing three Pseudomonas milk isolates and interrogating specific similarities to cystic fibrosis clinical isolate, P. lundensis AU1044*. PhD thesis, University of Michigan, 2020.

[208] Qingqing Fang, Yu Feng, Ping Feng, Xiaohui Wang, and Zhiyong Zong. Nosocomial bloodstream infection and the emerging carbapenem-resistant pathogen ralstonia insidiosa. *BMC Infectious Diseases*, 19(1):1–9, 2019.

[209] Shuang Wang, Liuchen Xu, Xiaohui Chi, Yan Li, Zengqiang Kou, Peibin Hou, Hengjie Xie, Zhenwang Bi, and Beiwen Zheng. Emergence of ndm-1-and ctx-m-3-producing raoultella ornithinolytica in human gut microbiota. *Frontiers in microbiology*, 10:2678, 2019.

[210] Piseth Seng, Boushab Mohamed Boushab, Fanny Romain, Frédérique Gouriet, Nicolas Bruder, Claude Martin, Franck Paganelli, Emmanuelle Bernit, Yves Patrice Le Treut, Pascal Thomas, et al. Emerging role of raoultella ornithinolytica in human infections: a series of cases and review of the literature. *International Journal of Infectious Diseases*, 45:65–71, 2016.

[211] F Aujoulat, H Marchandin, I Zorgniotti, A Masnou, and E Jumas-Bilak. Rhizobium pusense is the main human pathogen in the genus agrobacterium/rhizobium. *Clinical Microbiology and Infection*, 21(5):472–e1, 2015.

[212] C Léopold Kurz, Sophie Chauvet, Emmanuel Andrès, Marianne Aurouze, Isabelle Vallet, Gérard PF Michel, Mitch Uh, Jean Celli, Alain Filloux, Sophie de Bentzmann, et al. Virulence factors of the human opportunistic pathogen serratia marcescens identified by in vivo screening. *The EMBO Journal*, 22(7):1451–1460, 2003.

[213] Anna Lavecchia, Matteo Chiara, Caterina De Virgilio, Caterina Manzari, Rosa Monno, Armando De Carlo, Carlo Pazzani, David Horner, Graziano Pesole, and Antonio Placido. Staphylococcus arlettae genomics: novel insights on candidate antibiotic resistance and virulence genes in an emerging opportunistic pathogen. *Microorganisms*, 7(11):580, 2019.

[214] Steven YC Tong, Joshua S Davis, Emily Eichenberger, Thomas L Holland, and Vance G Fowler. Staphylococcus aureus infections: epidemiology, pathophysiology, clinical manifestations, and management. *Clinical Microbiology Reviews*, 28(3):603–661, 2015.

[215] PATRIC Database. Staphylococcus aureus strain SA15KEN strain not applicable, Genome ID 1280.23630. `https://www.patricbrc.org/view/Genome/1280.23630`.

[216] David Cameron, Jhih-Hang Jiang, Karl Hassan, Liam Elbourne, Kellie Tuck, Ian Paulsen, and Anton Peleg. Insights on virulence from the complete genome of staphylococcus capitis. *Frontiers in Microbiology*, 6:980, 2015.

[217] Jorunn Pauline Cavanagh, Maria Pain, Fatemeh Askarian, Jack-Ansgar Bruun, Ilona Urbarova, Sun Nyunt Wai, Frank Schmidt, and Mona Johannessen. Comparative exoproteome profiling of an invasive and a commensal staphylococcus haemolyticus isolate. *Journal of Proteomics*, 197:106–114, 2019.

[218] PATRIC Database. Stenotrophomonas indicatrix strain AS012656, Genome ID 2045451.27. `https://www.patricbrc.org/view/Genome/2045451.27`.

[219] Mareike Weber, Wiebke Schünemann, Janina Fuß, Peter Kämpfer, and André Lipski. Stenotrophomonas lactitubi sp. nov. and stenotrophomonas indicatrix sp. nov., isolated from surfaces with food contact. *International Journal of Systematic and Evolutionary Microbiology*, 68(6):1830–1838, 2018.

[220] V Kalidasan, Narcisse Joseph, Suresh Kumar, Rukman Awang Hamat, and Vasantha Kumari Neela. Iron and virulence in stenotrophomonas maltophilia: all we know so far. *Frontiers in Cellular and Infection Microbiology*, 8:401, 2018.

[221] Miki Matsue, Kohei Ogura, Hironori Sugiyama, Tohru Miyoshi-Akiyama, Yukiko Takemori-Sakai, Yasunori Iwata, Takashi Wada, and Shigefumi Okamoto. Pathogenicity characterization of prevalent-type streptococcus dysgalactiae subsp. equisimilis strains. *Frontiers in microbiology*, 11:97, 2020.

[222] Kirsi Aaltonen, Ravi Kant, Marjut Eklund, Mirja Raunio-Saarnisto, Lars Paulin, Olli Vapalahti, Thomas Grönthal, Merja Rantala, and Tarja Sironen. Streptococcus halichoeri: Comparative genomics of an emerging pathogen. *International Journal of Genomics*, 2020, 2020.

[223] Stephanie W Lo, Rebecca A Gladstone, Andries J Van Tonder, John A Lees, Mignon Du Plessis, Rachel Benisty, Noga Givon-Lavi, Paulina A Hawkins, Jennifer E Cornick, Brenda Kwambana-Adams, et al. Pneumococcal lineages associated with serotype replacement and antibiotic resistance in childhood invasive pneumococcal disease in the post-pcv13 era: an international whole-genome sequencing study. *The Lancet Infectious Diseases*, 19(7):759–769, 2019.

[224] Jeffrey N Weiser, Daniela M Ferreira, and James C Paton. Streptococcus pneumoniae: transmission, colonization and invasion. *Nature Reviews Microbiology*, 16(6):355–367, 2018.

[225] Lucia Gonzales-Siles, Roger Karlsson, Patrik Schmidt, Francisco Salvà-Serra, Daniel Jaén-Luchoro, Susann Skovbjerg, Edward RB Moore, and Margarita Gomila. A pangenome approach for discerning species-unique gene markers for identifications of streptococcus pneumoniae and streptococcus pseudopneumoniae. *Frontiers in Cellular and Infection Microbiology*, 10:222, 2020.

[226] Muge Aydin Tufan, Kart-Koseoglu Hamide, Ersozlu-Bozkirli Duygu, Azap Ozlem, Tufan Kadir, and Yucel Ahmet Eftal. Spondylodiscitis and endocarditis caused by s. vestibularis. *Brazilian Journal of Infectious Diseases*, 14(4):377–379, 2010.

[227] PATRIC Database. Streptococcus vestibularis strain as012761, Genome ID 1343.51. `https://www.patricbrc.org/view/Genome/1343.51`.

[228] Lv Wang, Claus T Christophersen, Michael J Sorich, Jacobus P Gerber, Manya T Angley, and Michael A Conlon. Increased abundance of sutterella spp. and ruminococcus torques in feces of children with autism spectrum disorder. *Molecular Autism*, 4(1):42, 2013.

[229] PATRIC Database. Sutterella wadsworthensis strain 809h, Genome ID 40545.1254. `https://www.patricbrc.org/view/Genome/40545.1254`.

[230] Di Zhao, Shangong Wu, Wenwen Feng, Ivan Jakovlić, Ngoc Tuan Tran, and Fan Xiong. Adhesion and colonization properties of potentially probiotic bacillus paralicheniformis strain fa6 isolated from grass carp intestine. *Fisheries Science*, 86(1):153–161, 2020.

[231] PATRIC Database. Bacillus paralicheniformis strain 6-1, Genome ID 1648923.136. `https://www.patricbrc.org/view/Genome/1648923.136`.

[232] Fouad MF Elshaghabee, Namita Rokana, Rohini D Gulhane, Chetan Sharma, and Harsh Panwar. Bacillus as potential probiotics: status, concerns, and future perspectives. *Frontiers in Microbiology*, 8:1490, 2017.

[233] PATRIC Database. Bacillus subtilis strain 8-1, Genome ID 1423.1028. `https://www.patricbrc.org/view/Genome/1423.1028`.

[234] PATRIC Database. Bacillus velezensis strain Marseille-Q1230, Genome ID 492670.508. `https://www.patricbrc.org/view/Genome/492670.508`.

[235] Oleg N Reva, Dirk ZH Swanevelder, Liberata A Mwita, Aneth David Mwakilili, Dillon Muzondiwa, Monique Joubert, Wai Yin Chan, Stefanie Lutz, Christian H Ahrens, Lylia V Avdeeva, et al. Genetic, epigenetic and phenotypic diversity of four bacillus velezensis strains used for plant protection or as probiotics. *Frontiers in Microbiology*, 10:2610, 2019.

[236] PATRIC Database. Bacteroides ovatus strain F11, Genome ID 28116.1355. `https://www.patricbrc.org/view/Genome/28116.1355`.

[237] Huizi Tan, Zhiming Yu, Chen Wang, Qingsong Zhang, Jianxin Zhao, Hao Zhang, Qixiao Zhai, and Wei Chen. Pilot safety evaluation of a novel strain of bacteroides ovatus. *Frontiers in Genetics*, 9:539, 2018.

[238] PATRIC Database. Bacteroides uniformis strain A23, Genome ID 820.4961. `https://www.patricbrc.org/view/Genome/820.4961`.

[239] Alfonso Benítez-Páez, Eva M Gómez del Pulgar, and Yolanda Sanz. The glycolytic versatility of bacteroides uniformis cect 7771 and its genome response to oligo and polysaccharides. *Frontiers in Cellular and Infection Microbiology*, 7:383, 2017.

[240] M Poyet, M Groussin, SM Gibbons, J Avila-Pacheco, X Jiang, SM Kearney, AR Perrotta, B Berdy, S Zhao, TD Lieberman, et al. A library of human gut bacterial isolates paired with longitudinal multiomics data enables mechanistic microbiome research. *Nature Medicine*, 25(9):1442–1452, 2019.

[241] Philippe Ulsemer, Kawe Toutounian, Jens Schmidt, Uwe Karsten, and Steffen Goletz. Preliminary safety evaluation of a new bacteroides xylanisolvens isolate. *Applied and Environmental Microbiology*, 78(2):528–535, 2012.

[242] Anna Pompei, Lisa Cordisco, Alberto Amaretti, Simona Zanoni, Diego Matteuzzi, and Maddalena Rossi. Folate production by bifidobacteria as a potential probiotic property. *Applied and Environmental Microbiology*, 73(1):179–185, 2007.

[243] Gabriele Andrea Lugli, Walter Mancino, Christian Milani, Sabrina Duranti, Leonardo Mancabelli, Stefania Napoli, Marta Mangifesta, Alice Viappiani, Rosaria Anzalone, Giulia Longhi, et al. Dissecting the evolutionary development of the species bifidobacterium animalis through comparative genomics analyses. *Applied and environmental microbiology*, 85(7):e02806–18, 2019.

[244] Francesca Turroni, Sabrina Duranti, Francesca Bottacini, Simone Guglielmetti, Douwe Van Sinderen, and Marco Ventura. Bifidobacterium bifidum as an example of a specialized human gut commensal. *Frontiers in Microbiology*, 5:437, 2014.

[245] Erin Simpson, Fatemeh Sanjar, Dennis Wylie, Sowon Park, Hong Koh, Jaewoong Bae, Sun-Young Kook, Seokjin Kim, and Hyun Jung Kim. Draft genome sequences of two strains of bifidobacterium dentium isolated from a crude fecal extract used for fecal microbiota transplantation in the republic of korea. *Microbiology Resource Announcements*, 9(23):e00197–20, 2020.

[246] Chyn Boon Wong, Toshitaka Odamaki, and Jin-zhong Xiao. Beneficial effects of bifidobacterium longum subsp. longum bb536 on human health: Modulation of gut microbiome as the principal action. *Journal of Functional Foods*, 54:506–519, 2019.

[247] Guillaume A Durand, P-E Fournier, Didier Raoult, and Sophie Edouard. 'bittarella massiliensis' gen. nov., sp. nov. isolated by culturomics from the gut of a healthy 28-year-old man. *New Microbes and New Infections*, 16:28–29, 2017.

[248] Guillaume A Durand, Thao Pham, Sokhna Ndongo, Sory Ibrahima Traore, Grégory Dubourg, Jean-Christophe Lagier, Caroline Michelle, Nicholas Armstrong, Pierre-Edouard Fournier, Didier

Raoult, et al. Blautia massiliensis sp. nov., isolated from a fresh human fecal sample and emended description of the genus blautia. *Anaerobe*, 43:47–55, 2017.

[249] Diane Hatziioanou, Cristina Gherghisan-Filip, Gerhard Saalbach, Nikki Horn, Udo Wegmann, Sylvia H Duncan, Harry J Flint, Melinda J Mayer, and Arjan Narbad. Discovery of a novel lantibiotic nisin o from blautia obeum a2-162, isolated from the human gastrointestinal tract. *Microbiology*, 163(9):1292, 2017.

[250] PATRIC Database. Blautia sp. BIOML-A1, Genome ID 2584624.3. `https://www.patricbrc.org/view/Genome/2584624.3`.

[251] Chengxu Liu, Sydney M Finegold, Yuli Song, and Paul A Lawson. Reclassification of clostridium coccoides, ruminococcus hansenii, ruminococcus hydrogenotrophicus, ruminococcus luti, ruminococcus productus and ruminococcus schinkii as blautia coccoides gen. nov., comb. nov., blautia hansenii comb. nov., blautia hydrogenotrophica comb. nov., blautia luti comb. nov., blautia producta comb. nov., blautia schinkii comb. nov. and description of blautia wexlerae sp. nov., isolated from human faeces. *International Journal of Systematic and Evolutionary Microbiology*, 58(8):1896–1902, 2008.

[252] PATRIC Database. Butyricicoccus sp. BIOML-A1, Genome ID 2584625.3. `https://www.patricbrc.org/view/Genome/2584625.3`.

[253] PATRIC Database. Catenibacterium sp. BIOML-A1, Genome ID 2584626.3. `https://www.patricbrc.org/view/Genome/2584626.3`.

[254] Zhen Shi and Hans P Blaschek. Transcriptional analysis of clostridium beijerinckii ncimb 8052 and the hyper-butanol-producing mutant ba101 during the shift from acidogenesis to solventogenesis. *Applied and Environmental Microbiology*, 74(24):7709–7714, 2008.

[255] PATRIC Database. Coprobacillus sp. BIOML-A1, Genome ID 2584634.3. `https://www.patricbrc.org/view/Genome/2584634.3`.

[256] PATRIC Database. Coprococcus comes strain F22, Genome ID 410072.542. `https://www.patricbrc.org/view/Genome/410072.542`.

[257] PATRIC Database. Coprococcus sp. BIOML-A1, Genome ID 2584635.3. `https://www.patricbrc.org/view/Genome/2584635.3`.

[258] David Taras, Rainer Simmering, Matthew D Collins, Paul A Lawson, and Michael Blaut. Reclassification of eubacterium formicigenerans holdeman and moore 1974 as dorea formicigenerans gen. nov., comb. nov., and description of dorea longicatena sp. nov., isolated from human faeces. *International Journal of Systematic and Evolutionary Microbiology*, 52(2):423–428, 2002.

[259] PATRIC Database. Dorea sp. BIOML-A1, Genome ID 2584637.3. `https://www.patricbrc.org/view/Genome/2584637.3`.

[260] Gina Paola Rodriguez-Castaño, Matthew R Dorris, Xingbo Liu, Bradley W Bolling, Alejandro Acosta-Gonzalez, and Federico E Rey. Bacteroides thetaiotaomicron starch utilization promotes quercetin degradation and butyrate production by eubacterium ramulus. *Frontiers in Microbiology*, 10:1145, 2019.

[261] Andreas Schwiertz, Gwenaelle Le Blay, and Michael Blaut. Quantification of different eubacteriumspp. in human fecal samples with species-specific 16s rrna-targeted oligonucleotide probes. *Applied and Environmental Microbiology*, 66(1):375–382, 2000.

[262] Mireia Lopez-Siles, Sylvia H Duncan, L Jesús Garcia-Gil, and Margarita Martinez-Medina. Faecalibacterium prausnitzii: from microbiology to diagnostics and prognostics. *The ISME Journal*, 11(4):841–852, 2017.

[263] Leandro Benevides, Sriti Burman, Rebeca Martin, Véronique Robert, Muriel Thomas, Sylvie Miquel, Florian Chain, Harry Sokol, Luis G Bermudez-Humaran, Mark Morrison, et al. New insights into the diversity of the genus faecalibacterium. *Frontiers in Microbiology*, 8:1790, 2017.

[264] Tasuku Ogita, Yoshinari Yamamoto, Ayane Mikami, Suguru Shigemori, Takashi Sato, and Takeshi Shimosato. Oral administration of flavonifractor plautii strongly suppresses th2 immune responses in mice. *Frontiers in Immunology*, 11:379, 2020.

[265] Ashley Toney, Yibo Xian, Jing Shao, Robert Schmaltz, Virginia Chaidez, Soonkyu Chung, and Amanda Ramer-Tait. Introducing gordonibacter urolithinfaciens into gut ecosystems to study its role in mediating the metabolic benefits of dietary polyphenols. *Current Developments in Nutrition*, 4(Supplement_2):1594–1594, 2020.

[266] MN Joshi, AS Pandit, A Sharma, RV Pandya, AK Saxena, and SB Bagatharia. Draft genome sequence of the halophilic bacterium halobacillus sp. strain bab-2008. *Genome Announcements*, 1(1), 2013.

[267] Ajay Kumar Mishra, Jean-Christophe Lagier, Anne Pfleiderer, Thi Thien Nguyen, Aurelia Caputo, Didier Raoult, and Pierre-Edouard Fournier. Non-contiguous finished genome sequence and description of holdemania massiliensis sp. nov. *Standards in Genomic Sciences*, 9(2):395–409, 2013.

[268] Karim Naghmouchi, Yanath Belguesmia, Farida Bendali, Giuseppe Spano, Bruce S Seal, and Djamel Drider. Lactobacillus fermentum: A bacterial species with potential for food preservation and biomedical applications. *Critical Reviews in Food Science and Nutrition*, 60(20):3387–3399, 2020.

[269] Valentina Taverniti and Simone Guglielmetti. Health-promoting properties of lactobacillus helveticus. *Frontiers in Microbiology*, 3:392, 2012.

[270] Ji Hyun Choi, Chang Mo Moon, Tae-Seop Shin, Eun Kyoung Kim, Andrea McDowell, Min-Kyung Jo, Yang Hee Joo, Seong-Eun Kim, Hye-Kyung Jung, Ki-Nam Shim, et al. Lactobacillus paracasei-derived extracellular vesicles attenuate the intestinal inflammatory response by

augmenting the endoplasmic reticulum stress pathway. *Experimental & Molecular Medicine*, 52(3):423–437, 2020.

[271] Nieke Westerik, Remco Kort, Wilbert Sybesma, and Gregor Reid. Lactobacillus rhamnosus probiotic food as a tool for empowerment across the value chain in africa. *Frontiers in Microbiology*, 9:1501, 2018.

[272] Xia Yu, Silja Åvall-Jääskeläinen, Joanna Koort, Agneta Lindholm, Johanna Rintahaka, Ingemar von Ossowski, Airi Palva, and Ulla Hynönen. A comparative characterization of different host-sourced lactobacillus ruminis strains and their adhesive, inhibitory, and immunomodulating functions. *Frontiers in Microbiology*, 8:657, 2017.

[273] Thomas Clavel, Ramona Lippman, Françoise Gavini, Joël Doré, and Michael Blaut. Clostridium saccharogumia sp. nov. and lactonifactor longoviformis gen. nov., sp. nov., two novel human faecal bacteria involved in the conversion of the dietary phytoestrogen secoisolariciresinol diglucoside. *Systematic and Applied Microbiology*, 30(1):16–26, 2007.

[274] Sudarshan Anand Shetty, Nachiket Prakash Marathe, Vikram Lanjekar, Dilip Ranade, and Yogesh S Shouche. Comparative genome analysis of megasphaera sp. reveals niche specialization and its potential role in the human gut. *PloS One*, 8(11):e79353, 2013.

[275] Kaisa Hiippala, Gonçalo Barreto, Claudia Burrello, Angelica Diaz-Basabe, Maiju Suutarinen, Veera Kainulainen, Jolene R Bowers, Darrin Lemmer, David M Engelthaler, Kari K Eklund, et al. Novel odoribacter splanchnicus strain and its outer membrane vesicles exert immunoregulatory effects in vitro. *Frontiers in Microbiology*, 11:2906, 2020.

[276] Kai Wang, Mingfang Liao, Nan Zhou, Li Bao, Ke Ma, Zhongyong Zheng, Yujing Wang, Chang Liu, Wenzhao Wang, Jun Wang, et al. Parabacteroides distasonis alleviates obesity and metabolic dysfunctions via production of succinate and secondary bile acids. *Cell Reports*, 26(1):222–235, 2019.

[277] Tsung-Ru Wu, Chuan-Sheng Lin, Chih-Jung Chang, Tzu-Lung Lin, Jan Martel, Yun-Fei Ko, David M Ojcius, Chia-Chen Lu, John D Young, and Hsin-Chih Lai. Gut commensal parabacteroides goldsteinii plays a predominant role in the anti-obesity effects of polysaccharides isolated from hirsutella sinensis. *Gut*, 68(2):248–262, 2019.

[278] Mitsuo Sakamoto and Yoshimi Benno. Reclassification of bacteroides distasonis, bacteroides goldsteinii and bacteroides merdae as parabacteroides distasonis gen. nov., comb. nov., parabacteroides goldsteinii comb. nov. and parabacteroides merdae comb. nov. *International Journal of Systematic and Evolutionary Microbiology*, 56(7):1599–1605, 2006.

[279] Tingting Ju, Ji Yoon Kong, Paul Stothard, and Benjamin P Willing. Defining the role of parasutterella, a previously uncharacterized member of the core gut microbiota. *The ISME Journal*, 13(6):1520–1534, 2019.

[280] Feifan Wu, Xianfeng Guo, Jiachun Zhang, Min Zhang, Zihao Ou, and Yongzheng Peng. Phascolarctobacteriumáfaecium abundant colonization in human gastrointestinal tract. *Experimental and Therapeutic Medicine*, 14(4):3122–3126, 2017.

[281] PATRIC Database. Piscibacillus halophilus strain Marseille-Q1613, Genome ID 571933.10. `https://www.patricbrc.org/view/Genome/571933.10`.

[282] D Ricaboni, M Mailhe, A Benezech, C Andrieu, P-E Fournier, and Didier Raoult. 'pseudoflavonifractor phocaeensis' gen. nov., sp. nov., isolated from human left colon. *New Microbes and New Infections*, 17:15–17, 2017.

[283] Sylvia H Duncan, Rustam I Aminov, Karen P Scott, Petra Louis, Thaddeus B Stanton, and Harry J Flint. Proposal of roseburia faecis sp. nov., roseburia hominis sp. nov. and roseburia inulinivorans sp. nov., based on isolates from human faeces. *International Journal of Systematic and Evolutionary Microbiology*, 56(10):2437–2441, 2006.

[284] Sabina Leanti La Rosa, Maria Louise Leth, Leszek Michalak, Morten Ejby Hansen, Nicholas A Pudlo, Robert Glowacki, Gabriel Pereira, Christopher T Workman, Magnus Ø Arntzen, Phillip B Pope, et al. The human gut firmicute roseburia intestinalis is a primary degrader of dietary β-mannans. *Nature Communications*, 10(1):1–14, 2019.

[285] Xiaolei Ze, Yonit Ben David, Jenny A Laverde-Gomez, Bareket Dassa, Paul O Sheridan, Sylvia H Duncan, Petra Louis, Bernard Henrissat, Nathalie Juge, Nicole M Koropatkin, et al. Unique organization of extracellular amylases into amylosomes in the resistant starch-utilizing human colonic firmicutes bacterium ruminococcus bromii. *MBio*, 6(5):e01058–15, 2015.

[286] Min-Soo Kim, Seong Woon Roh, and Jin-Woo Bae. Ruminococcus faecis sp. nov., isolated from human faeces. *The Journal of Microbiology*, 49(3):487–491, 2011.

[287] Andrei N Shkoporov, Andrei V Chaplin, Victoria A Shcherbakova, Natalia E Suzina, Lyudmila I Kafarskaia, Vladimir K Bozhenko, and Boris A Efimov. Ruthenibacterium lactatiformans gen. nov., sp. nov., an anaerobic, lactate-producing member of the family ruminococcaceae isolated from human faeces. *International Journal of Systematic and Evolutionary Microbiology*, 66:3041–3049, 2016.

[288] Maria Pain, Runa Wolden, Daniel Jaén-Luchoro, Francisco Salvà-Serra, Beatriz Piñeiro Iglesias, Roger Karlsson, Claus Klingenberg, and Jorunn Pauline Cavanagh. Staphylococcus borealis sp. nov., isolated from human skin and blood. *International Journal of Systematic and Evolutionary Microbiology*, 70(12):6067–6078, 2020.

[289] Ghalia Kaci, Denise Goudercourt, Véronique Dennin, Bruno Pot, Joel Dore, S Dusko Ehrlich, Pierre Renault, Hervé M Blottière, Catherine Daniel, and Christine Delorme. Anti-inflammatory properties of streptococcus salivarius, a commensal bacterium of the oral cavity and digestive tract. *Applied and Environmental Microbiology*, 80(3):928–934, 2014.

[290] Kim Holmstrøm, Matthew D Collins, Trine Møller, Enevold Falsen, and Paul A Lawson. Subdoligranulum variabile gen. nov., sp. nov. from human feces. *Anaerobe*, 10(3):197–203, 2004.

[291] Abigail L Glascock, Nicole R Jimenez, Sam Boundy, Vishal N Koparde, J Paul Brooks, David J Edwards, Jerome F Strauss III, Kimberly K Jefferson, Myrna G Serrano, Gregory A Buck, et al. Unique roles of vaginal megasphaera phylotypes in reproductive health. *Microbial Genomics*, 7(12):000526, 2021.

[292] PATRIC Database. Veillonellaceae bacterium M2-8, Genome ID 2093855.3. `https://www.patricbrc.org/view/Genome/2093855.3`.

[293] Wing Sun Faith Chung, Marjolein Meijerink, Birgitte Zeuner, Jesper Holck, Petra Louis, Anne S Meyer, Jerry M Wells, Harry J Flint, and Sylvia H Duncan. Prebiotic potential of pectin and pectic oligosaccharides to promote anti-inflammatory commensal bacteria in the human colon. *FEMS Microbiology Ecology*, 93(11):fix127, 2017.

[294] Jaime A Rosero, Jirˇí Killer, Hana Sechovcová, Jakub Mrázek, Oldrˇich Benada, Katerˇina Fliegerová, Jaroslav Havlík, and Jan Kopečnỳ. Reclassification of eubacterium rectale (hauduroy et al. 1937) prévot 1938 in a new genus agathobacter gen. nov. as agathobacter rectalis comb. nov., and description of agathobacter ruminis sp. nov., isolated from the rumen contents of sheep and cows. *International Journal of Systematic and Evolutionary Microbiology*, 66(2):768–773, 2016.

[295] Amadou Hamidou Togo, Awa Diop, Fadi Bittar, Marie Maraninchi, René Valero, Nicholas Armstrong, Grégory Dubourg, Noémie Labas, Magali Richez, Jeremy Delerce, et al. Description of mediterraneibacter massiliensis, gen. nov., sp. nov., a new genus isolated from the gut microbiota of an obese patient and reclassification of ruminococcus faecis, ruminococcus lactaris, ruminococcus torques, ruminococcus gnavus and clostridium glycyrrhizinilyticum as mediterraneibacter faecis comb. nov., mediterraneibacter lactaris comb. nov., mediterraneibacter torques comb. nov., mediterraneibacter gnavus comb. nov. and mediterraneibacter glycyrrhizinilyticus comb. nov. *Antonie van Leeuwenhoek*, 111(11):2107–2128, 2018.

[296] Bianca J Parker, Pamela A Wearsch, Alida Veloo, and Alex Rodriguez-Palacios. The genus alistipes: gut bacteria with emerging implications to inflammation, cancer, and mental health. *Frontiers in immunology*, 11:906, 2020.

[297] Kui Zhu, Christina S Hölzel, Yifang Cui, Ricarda Mayer, Yang Wang, Richard Dietrich, Andrea Didier, Rupert Bassitta, Erwin Märtlbauer, and Shuangyang Ding. Probiotic bacillus cereus strains, a potential risk for public health in china. *Frontiers in Microbiology*, 7:718, 2016.

[298] PATRIC Database. Bacillus cereus strain 2-1, Genome ID 1396.2563. `https://www.patricbrc.org/view/Genome/1396.2563`.

[299] Isabella Princess, T Natarajan, and Siddhartha Ghosh. When good bacteria behave badly: a case report of bacillus clausii sepsis in an immunocompetant adult. *Access Microbiology*, 2(4):e000097, 2020.

[300] Roibeard F Thornton, Elizabeth C Murphy, Todd F Kagawa, Paul W O'Toole, and Jakki C Cooney. The effect of environmental conditions on expression of bacteroides fragilis and bacteroides thetaiotaomicron c10 protease genes. *BMC Microbiology*, 12(1):1–11, 2012.

[301] PATRIC Database. Bacteroides thetaiotaomicron strain F9-2, Genome ID 818.1282. `https://www.patricbrc.org/view/Genome/818.1282`.

[302] Pßraic Ë CuÝv, Tomas De Wouters, Rabina Giri, Stanislas Mondot, Wendy J Smith, HervÚ M BlottiŔre, Jakob Begun, and Mark Morrison. The gut bacterium and pathobiont bacteroides vulgatus activates nf-κb in a human gut epithelial cell line in a strain and growth phase dependent manner. *Anaerobe*, 47:209–217, 2017.

[303] PJ Perez-Chaparro, C Gonçalves, LC Figueiredo, M Faveri, E Lobão, N Tamashiro, P Duarte, and M Feres. Newly identified pathogens associated with periodontitis: a systematic review. *Journal of Dental Research*, 93(9):846–858, 2014.

[304] Luigi Nibali, Vanessa Sousa, Mehmet Davrandi, David Spratt, Qumasha Alyahya, Jose Dopico, and Nikos Donos. Differences in the periodontal microbiome of successfully treated and persistent aggressive periodontitis. *Journal of Clinical Periodontology*, 47(8):980–990, 2020.

[305] Anne Jolivet-Gougeon, Jean-Louis Sixou, Zohreh Tamanai-Shacoori, and Martine Bonnaure-Mallet. Antimicrobial treatment of capnocytophaga infections. *International Journal of Antimicrobial Agents*, 29(4):367–373, 2007.

[306] V Garcia, C Abat, V Moal, and J-M Rolain. Citrobacter amalonaticus human urinary tract infections, marseille, france. *New Microbes and New Infections*, 11:1–5, 2016.

[307] N Cassir, S Benamar, and B La Scola. Clostridium butyricum: from beneficial to a new emerging pathogen. *Clinical Microbiology and Infection*, 22(1):37–45, 2016.

[308] F Morio, H Jean-Pierre, L Dubreuil, E Jumas-Bilak, L Calvet, G Mercier, R Devine, and H Marchandin. Antimicrobial susceptibilities and clinical sources of dialister species. *Antimicrobial Agents and Chemotherapy*, 51(12):4498–4501, 2007.

[309] Bradley J Gardiner, AY Tai, Despina Kotsanas, Michelle J Francis, Sally A Roberts, Susan A Ballard, Ralph K Junckerstorff, and Tony M Korman. Clinical and microbiological characteristics of eggerthella lenta bacteremia. *Journal of Clinical Microbiology*, 53(2):626–635, 2015.

[310] Armand Paauw, Martien PM Caspers, Maurine A Leverstein-van Hall, Frank HJ Schuren, Roy C Montijn, Jan Verhoef, and Ad C Fluit. Identification of resistance and virulence factors in an epidemic enterobacter hormaechei outbreak strain. *Microbiology*, 155(5):1478–1488, 2009.

[311] Michael S Gilmore, Don B Clewell, Yasuyoshi Ike, and Nathan Shankar. Enterococci: From commensals to leading causes of drug resistant infection [internet]. 2014.

[312] Byung-Han Ryu, Jeongmin Hong, Jiwon Jung, Min Jae Kim, Heungsup Sung, Mi-Na Kim, Yong Pil Chong, Sung-Han Kim, Sang-Oh Lee, Yang Soo Kim, et al. Clinical characteristics and treatment outcomes of enterococcus durans bacteremia: a 20-year experience in a tertiary care hospital. *European Journal of Clinical Microbiology & Infectious Diseases*, 38(9):1743–1751, 2019.

[313] Zixin Peng, Menghan Li, Wei Wang, Hongtao Liu, Séamus Fanning, Yujie Hu, Jianzhong Zhang, and Fengqin Li. Genomic insights into the pathogenicity and environmental adaptability of enterococcus hirae r17 isolated from pork offered for retail sale. *Microbiologyopen*, 6(6):e00514, 2017.

[314] Tomomi Higashide, Mami Takahashi, Akira Kobayashi, Shinji Ohkubo, Mayumi Sakurai, Yutaka Shirao, Toshihiro Tamura, and Kazuhisa Sugiyama. Endophthalmitis caused by enterococcus mundtii. *Journal of Clinical Microbiology*, 43(3):1475–1476, 2005.

[315] Bettina Sigrid Freyaldenhoven, Georg Schlieper, Rudolf Lütticken, and Ralf Réné Reinert. Enterococcus raffinosus infection in an immunosuppressed patient: case report and review of the literature. *Journal of Infection*, 51(3):e121–e124, 2005.

[316] Ariane Neumann, Lars Björck, and Inga-Maria Frick. Finegoldia magna, an anaerobic gram-positive bacterium of the normal human microbiota, induces inflammation by activating neutrophils. *Frontiers in Microbiology*, 11:65, 2020.

[317] Holger Brüggemann, Anders Jensen, Seven Nazipi, Hüsnü Aslan, Rikke Louise Meyer, Anja Poehlein, Elzbieta Brzuszkiewicz, Munir A Al-Zeer, Volker Brinkmann, and Bo Söderquist. Pan-genome analysis of the genus finegoldia identifies two distinct clades, strain-specific heterogeneity, and putative virulence factors. *Scientific Reports*, 8(1):1–11, 2018.

[318] Yali Cao, Min Qiao, Zhigang Tian, Yan Yu, Baohua Xu, Wansheng Lao, Xuguo Ma, and Wenge Li. Comparative analyses of subgingival microbiome in chronic periodontitis patients with and without iga nephropathy by high throughput 16s rrna sequencing. *Cellular Physiology and Biochemistry*, 47(2):774–783, 2018.

[319] Patrick CY Woo, Jade LL Teng, Kay KM Lam, WS Cindy, Kit-Wah Leung, Anthony WS Leung, Susanna KP Lau, and Kwok-Yung Yuen. First report of gordonibacter pamelaeae bacteremia. *Journal of Clinical Microbiology*, 48(1):319–322, 2010.

[320] Chun Yang, Di Wang, Qi Zhou, and Jiancheng Xu. Bacteremia due to vancomycin-resistant leuconostoc lactis in a patient with pneumonia and abdominal infection. *The American Journal of the Medical Sciences*, 349(3):282–283, 2015.

[321] Joon Yong Kim, Yeon Bee Kim, Hye Seon Song, Won-Hyong Chung, Changsu Lee, Seung Woo Ahn, Se Hee Lee, Min Young Jung, Tae-Woon Kim, Young-Do Nam, et al. Genomic analysis of a pathogenic bacterium, paeniclostridium sordellii cba7122 containing the highest number of rrna operons, isolated from a human stool sample. *Frontiers in Pharmacology*, 8:840, 2017.

[322] Patrizia Posteraro, Flavio De Maio, Giulia Menchinelli, Ivana Palucci, Federica Maria Errico, Mariantonietta Carbone, Maurizio Sanguinetti, Antonio Gasbarrini, and Brunella Posteraro. First bloodstream infection caused by prevotella copri in a heart failure elderly patient with prevotella-dominated gut microbiota: a case report. *Gut Pathogens*, 11(1):44, 2019.

[323] Zachary D Wallen, Mary Appah, Marissa N Dean, Cheryl L Sesler, Stewart A Factor, Eric Molho, Cyrus P Zabetian, David G Standaert, and Haydeh Payami. Characterizing dysbiosis of gut microbiome in pd: Evidence for overabundance of opportunistic pathogens. *npj Parkinson's Disease*, 6(1):1–12, 2020.

[324] Michael Buhl and Matthias Marschal. Prevotella vespertina sp. nov., isolated from an abscess of a hospital patient. *International Journal of Systematic and Evolutionary Microbiology*, 70(8):4576–4582, 2020.

[325] PATRIC Database. Prevotella sp. strain P-A-F_MAG_00002, Genome ID 59823.699. https://www.patricbrc.org/view/Genome/59823.699.

[326] Michael Otto. Staphylococcus epidermidis — the 'accidental' pathogen. *Nature Reviews Microbiology*, 7(8):555–567, 2009.

[327] PATRIC Database. Staphylococcus epidermidis strain JH, Genome ID 1282.3805. https://www.patricbrc.org/view/Genome/1282.3805.

[328] Vanessa N Raabe and Andi L Shane. Group b streptococcus (streptococcus agalactiae). *Microbiology spectrum*, 7(2):7–2, 2019.

[329] Qiurong Chen, Guojun Wu, Hui Chen, Hui Li, Shuo Li, Chenhong Zhang, Xiaoyan Pang, Linghua Wang, Liping Zhao, and Jian Shen. Quantification of human oral and fecal streptococcus parasanguinis by use of quantitative real-time pcr targeting the groel gene. *Frontiers in Microbiology*, 10:2910, 2019.

[330] Wirginia Krzyściak, KK Pluskwa, Anna Jurczak, and Dorota Kościelniak. The pathogenicity of the streptococcus genus. *European Journal of Clinical Microbiology & Infectious Diseases*, 32(11):1361–1376, 2013.

[331] Fernando Cobo, Virginia Pérez-Carrasco, José A García-Salcedo, and José María Navarro-Marí. Bacteremia caused by veillonella dispar in an oncological patient. *Anaerobe*, 66:102285, 2020.

[332] Steven Knapp, Clint Brodal, John Peterson, Fengxia Qi, Jens Kreth, and Justin Merritt. Natural competence is common among clinical isolates of veillonella parvula and is useful for genetic

manipulation of this key member of the oral microbiome. *Frontiers in Cellular and Infection Microbiology*, 7:139, 2017.

[333] J-H Chia, Y Feng, L-H Su, T-L Wu, C-L Chen, Y-H Liang, and C-H Chiu. Clostridium innocuum is a significant vancomycin-resistant pathogen for extraintestinal clostridial infection. *Clinical Microbiology and Infection*, 23(8):560–566, 2017.
